# The Loewner Energy via the Renormalised Energy of Moving Frames

**DOI:** 10.1007/s00205-024-01957-1

**Published:** 2024-02-12

**Authors:** Alexis Michelat, Yilin Wang

**Affiliations:** 1 EPFL B, Station 8, CH-1015 Lausanne, Switzerland; 2 Institut des Hautes Études Scientifiques, Bures-sur-Yvette, France

**Keywords:** 53C42, 30C35

## Abstract

We obtain a new formula for the Loewner energy of Jordan curves on the sphere, which is a Kähler potential for the essentially unique Kähler metric on the Weil–Petersson universal Teichmüller space, as the renormalised energy of moving frames on the two domains of the sphere delimited by the given curve.

## Introduction

### Background on Weil–Petersson Quasicircles

In [43, 58], the second author and S. Rohde introduced the Möbius-invariant Loewner energy to measure the roundness of Jordan curves on the Riemann sphere $\mathbb {C}\cup \{\infty \}$ using the Loewner transform [35]. The original motivation comes from the probabilistic theory of Schramm–Loewner evolutions, see, e.g., [60] for an overview. The second author proved in [59] that the Loewner energy is proportional to the *universal Liouville action* introduced by L. A. Takhtajan and L.-P. Teo [53]. In particular, the class of finite energy curves corresponds exactly to the *Weil–Petersson* class of quasicircles which has already been extensively studied by both physicists and mathematicians since the eighties, see, *e.g.*, [6, 10, 18, 22, 24, 30, 39, 40, 45, 48, 49, 53, 55, 56, 61], and is still an active research area. See the introduction of [6] (see also the companion papers [7, 8] for more on this topic) for a summary and a list of equivalent definitions of very different nature.

In this article, we sometimes view Jordan curves as curves on $S^2 \subset \mathbb {R}^3$ and give new characterisations of the Loewner energy in terms of the moving frames on $S^2$. Note that in this article, $S^2$ refers to the sphere of radius 1 centred at the origin in $\mathbb {R}^3$ equipped with the induced round metric $g_0$ from its embedding into $\mathbb {R}^3$. Therefore, $S^2$ is isometric to $\widehat{\mathbb {C}}=\mathbb {C}\cup \left\{ \infty \right\}$ endowed with the metric

$$\begin{aligned} g_{\widehat{\mathbb {C}}}=\frac{4|dz|^2}{(1+|z|^2)^2} \end{aligned}$$

by the stereographic projection. To distinguish the two setups, we will let $\gamma$ denote a Jordan curve in $\widehat{\mathbb {C}}$ and let $\Gamma$ denote a Jordan curve in $S^2$. Let us first list a few equivalent definitions of Weil–Petersson quasicircles that are relevant to this work.

#### Theorem 1.1

(Cui, [18], Tahktajan-Teo, [53], Shen, [49], Bishop, [6]) Let $\gamma \subset \mathbb C$ be a Jordan curve, $\Omega$ be the bounded connected component of $\mathbb {C}\setminus \gamma$, and let $f:\mathbb {D}\rightarrow \Omega$ and $g:\mathbb {C}\setminus \overline{\mathbb {D}}\rightarrow \mathbb {C}\setminus \overline{\Omega }$ be biholomorphic maps such that $g (\infty ) = \infty$. The following conditions are equivalent:

1. There exists a quasiconformal extension of *g* to $\mathbb {C}$ such that the Beltrami coefficient $\mu = \dfrac{\partial _{\overline{z}} g}{\partial _z g} :\mathbb D \rightarrow \mathbb D$ of $g|_{\mathbb D}$ satisfies $\displaystyle \int _{\mathbb {D}}|\mu (z)|^2\frac{|dz|^2}{(1-|z|^2)^2}<\infty$.
2. $$\displaystyle \int _{\mathbb {D}}|\nabla \log |f'(z)||^2|dz|^2=\int _{\mathbb {D}}\left| \frac{f''(z)}{f'(z)}\right| ^2|dz|^2<\infty $$.
3. $$\displaystyle \int _{\mathbb {C}\setminus \overline{\mathbb {D}}}\left| \frac{g''(z)}{g'(z)}\right| ^2|dz|^2<\infty $$.
4. The *(conformal) welding function* $\varphi =g^{-1}\circ f|_{S^1}$ satisfies $\log \varphi '$ belongs to the Sobolev space $H^{1/2}(S^1)$.
5. The curve $\gamma$ is chord-arc and the unit tangent $\tau :\gamma \rightarrow S^1$ belongs to $H^{1/2}(\gamma )$.
6. Every minimal surface $\Sigma \subset \mathbb {H}^3 \simeq \mathbb C \times \mathbb R_{+}^{*}$ with asymptotic boundary $\gamma$ has finite renormalised area, *i.e.*,
  1.1 $$\begin{aligned} \mathcal{R}\mathcal{A}(\Sigma )&= \lim \limits _{\varepsilon \rightarrow 0}\left( \textrm{Area}(\Sigma _\varepsilon )-\textrm{Length}(\partial \Sigma _\varepsilon )\right) \nonumber \\&=-2\pi \chi (\Sigma )-\int _{\Sigma }|\mathring{A}|^2d\textrm{vol}_{\Sigma }>-\infty , \end{aligned}$$

where for all $\varepsilon >0$, $\Sigma _\varepsilon =\Sigma \cap \left\{ (z,t): t>\varepsilon \right\}$ and $\partial \Sigma _\varepsilon =\Sigma \cap \left\{ (z,t): t=\varepsilon \right\}$.

If $\gamma$ satisfies any of those conditions, $\gamma$ is called a *Weil–Petersson quasicircle*.

The equivalence of (1) and (2) are due to G. Cui, and independently to Takhtajan and Teo who proved the equivalences (1), (2), (3). In (4), the continuous extension of *f*, *g* to $S^1$ is well-defined by a classical theorem of Carathéodory [13]. The equivalence between (1) and (4) is proved by Y. Shen. The second condition is perhaps the simplest one since it corresponds to the condition $\log |f'|\in W^{1,2}(\mathbb {D})$, the Sobolev space of functions with squared-integrable weak derivatives.

For (5), we recall that a Jordan curve is *chord-arc* if there exists $K<\infty$ such that for all $x,y\in \gamma$, we have $\ell (x,y)\le K|x-y|$, where $\ell (x,y)$ is the length of the shortest arc joining *x* to *y*. We mention that Weil–Petersson quasicircles are not only chord-arc but even *asymptotically smooth*, namely, the ratio $\ell (x,y)/|x-y|$ tends to 1 as *x* tends to *y*. These curves are not necessarily $C^1$, for they allow certain types of infinite spirals; see Section 6.1 for an explicit construction of such spirals (also [6, 43]). Recall that for any Jordan chord-arc curve $\gamma$, a function $u:\gamma \rightarrow \mathbb {C}$ belongs to the Sobolev space $H^{1/2}(\gamma )$ if and only if

1.2 $$\begin{aligned} \int _{\gamma }\int _{\gamma }\left| \frac{u(z)-u(w)}{z-w}\right| ^2|dz||dw|<\infty , \end{aligned}$$

where |*dz*| is the arc-length measure.

The equivalence between (1) and (5) was proven by Y. Shen and L. Wu ([52]; see also [28, 49–51]), and also by C. Bishop [6]. The last characterisation (6) due to Bishop [6] using the notion of renormalised area was first investigated for Willmore surfaces by S. Alexakis and R. Mazzeo [1, 2] which has strong motivations arising from string theory [27]. The integral of the squared trace-free second fundamental form $\mathring{A}$ in (6) is *the Willmore energy of* $\Sigma$ which is of particular interest for being conformally invariant. Amongst the important previous contribution that inspired this work, we should mention Epstein’s work [20, 21].

Not only can we characterise this class of curves qualitatively, as listed above, but there is an important quantity associated with each element of the class. Indeed, after appropriate normalisation, the class of Weil–Petersson quasicircles can be identified with the Weil–Petersson universal Teichmüller space $T_0(1)$ via conformal welding. Takhtajan and Teo [53] showed that $T_0(1)$ carries an essentially unique homogeneous Kähler metric and introduced the *universal Liouville action* $S_1$. They showed that $S_1$ is a Kähler potential on $T_0(1)$ which is of critical importance for the Kähler geometry. We take an analytic instead of a Teichmüller theoretic viewpoint, so we will consider $S_1$ as defined for Weil–Petersson quasicircles instead of their welding functions. Explicitly, for a Weil–Petersson quasicircle $\gamma$,

1.3 $$\begin{aligned} S_1 (\gamma )&=\int _{\mathbb {D}}\left| \frac{f''(z)}{f'(z)}\right| ^2|dz|^2+\int _{\mathbb {C}\setminus \overline{\mathbb {D}}}\left| \frac{g''(z)}{g'(z)}\right| ^2|dz|^2\nonumber \\&\quad +4\pi \log |f'(0)|-4\pi \log |g'(\infty )|. \end{aligned}$$

#### Theorem 1.2

(Y. Wang, [59]) A Jordan curve $\gamma$ has finite Loewner energy $I^L(\gamma )$ if and only if $\gamma$ is a Weil–Petersson quasicircle. Furthermore, we have

1.4 $$\begin{aligned} I^L(\gamma )=\frac{1}{\pi }S_1(\gamma ). \end{aligned}$$

We will therefore use interchangeably the terms “Jordan curve of finite Loewner energy”; “Weil–Petersson quasicircle”; or simply “Weil–Petersson curve”. As we did not define explicitly the Loewner energy $I^L(\gamma )$, readers may consider (1.4) as its definition. It may not be obvious from the expression of $S_1$ that it is invariant under Möbius transformations, such as the inversion ${\mathfrak {i}}: z\mapsto 1/z$, however, it would follow directly from the definition using Loewner transform in [43]. Provided that $\gamma$ separates 0 from $\infty$, we may choose the biholomorphic functions *f* and *g* as in Theorem 1.1 and assume further that $f(0)=0$. Applying the invariance of the Loewner energy under ${\mathfrak {i}}$, we get

1.5 $$\begin{aligned} I^L(\gamma )&= I^L({\mathfrak {i}}(\gamma ))\nonumber \\&=\frac{1}{\pi }\int _{\mathbb {D}}\left| \frac{f''(z)}{f'(z)}-2\frac{f'(z)}{f(z)}+\frac{2}{z}\right| ^2|dz|^2\nonumber \\&\quad +\frac{1}{\pi }\int _{\mathbb {C}\setminus \overline{\mathbb {D}}}\left| \frac{g''(z)}{g'(z)}-2\frac{g'(z)}{g(z)}+\frac{2}{z}\right| ^2|dz|^2+4\log |f'(0)|-4\log |g'(\infty )|. \end{aligned}$$

Before closing this first introductory section on the Loewner energy, let us mention two recent works of the second author [12, 14]. In the first article in collaboration with M. Carfagnini, the authors show that up to a universal constant involving the central charge of $\textrm{SLE}_{\kappa }$, for all $\kappa \le 4$, the Loewner energy is the Onsager–Machlup functional for the $\textrm{SLE}_{\kappa }$ loop measure on the space of simple closed curves. In the second article in collaboration with M. Bridgeman, K. Bromberg, and F. Vargas-Pallete, there are two fundamental results. The first one asserts that for any Weil–Petersson quasicircle, the signed volume between the two Epstein surfaces of the hyperbolic space $\mathbb {H}^3$ is finite. The second theorem shows a new identity for the Loewner energy in terms of a renormalised volume involving this sign volume and the integral of the mean curvature if $\gamma$ is a $C^{5,\alpha }$ regular curve. In general, the Loewner energy bounds from above this renormalised volume. Such a result is reminiscent of the previous equivalence shown by C. Bishop with the renormalised area of minimal surfaces of $\mathbb {H}^3$, but it has the advantage of giving a quantitative inequality.

### Moving Frames and the Ginzburg–Landau Equations

Moving frames, first introduced by Darboux in the late 19th century to study curves and surfaces, were later generalised by Élie Cartan and permit to reformulate astutely a wide class of differential-geometric problems. One now classical such use of this theory is found in the work of F. Hélein on harmonic maps [29], where the moving frames pave the way towards new regularity results. We give more details about this theory in Section 2.

In [34], P. Laurain and R. Petrides suggest a new approach to relate the Loewner energy to the renormalised energy of moving frames using the Ginzburg–Landau energy in a minimal regularity setting (which is of independent interest). Although the Ginzburg–Landau energy is normally used to construct harmonic maps with values into $S^1$ under topological constraints where no smooth solutions exist [5], it should be seen more generally as a way to construct (singular) moving frames on surfaces. Through this approach, one may hope to link quantitatively the Loewner energy and the Willmore energy that can also be written in terms of moving frames [37].

Let $\Omega \subset \mathbb {C}$ be a simply connected domain, and $\gamma =\partial \Omega$. In [34], it was shown that the Bethuel–Brezis–Hélein [5] analysis carries on for general chord-arc curves and $H^{1/2}$ boundary data. Using this delicate analysis [34], they obtained the following result, which is the most relevant to our present article:

#### Theorem 1.3

(Laurain–Petrides, [34], Theorem 0.2, Theorem 0.3) Let $\Omega \subset \mathbb {C}$ be a bounded simply connected domain such that $\gamma =\partial \Omega$ is a Weil–Petersson quasicircle. Then, there exists a harmonic map $\vec {u}:\Omega \setminus \left\{ p\right\} \rightarrow S^1$ with boundary data $\tau :\Gamma \rightarrow S^1$ which is the unit tangent vector of $\partial \Omega =\Gamma$. Let $\vec {v}=-i\,\vec {u}$ and $\omega =\langle \vec {u},d\vec {v}\,\rangle$, then there exists a harmonic function $\mu :\Omega \rightarrow \mathbb {R}$ such that $\omega =*\,d\left( G_{\Omega }+\mu \right)$, and a conformal map $f:\mathbb {D}\rightarrow \Omega$ such that $f(0) = p$, and

1.6 $$\begin{aligned} \left\{ \begin{aligned} \frac{1}{r}\partial _{\theta }f&=e^{\mu \circ f}\vec {u}\circ f\\ \partial _{r}f&=e^{\mu \circ f}\vec {v}\circ f. \end{aligned}\right. \end{aligned}$$

Furthermore, we have

1.7 $$\begin{aligned} \int _{\Omega }|\omega -*\,dG_{\Omega }|^2dx=\int _{\Omega }|\nabla \mu |^2\textrm{d}x=\int _{\mathbb {D}}\left| \frac{f''(z)}{f'(z)}\right| ^2|dz|^2, \end{aligned}$$

where $G_{\Omega }$ is a Green’s function with Dirichlet boundary condition on $\partial \Omega$.

The other main result of [34] is to identify the renormalised energy in the sense of Bethuel–Brezis–Hélein as an explicit term involving (1.7).

#### Remark 1.4

The harmonic function $\mu$ is explicitly given by $\mu =\log |\partial _{r}f|\circ f^{-1}=\log \left| \frac{1}{r}\partial _{\theta }f\right| \circ f^{-1}=\log |f'|\circ f^{-1}$. The last identities follow from the conformality of *f*. We note that in [34], the point *p* is a special point such that any biholomorphic map *f* with $f(0) = p$ maximizes $|f'(0)|$ amongst all biholomorphic maps $\mathbb D \rightarrow \Omega$. This property is equivalent to the fact that limits of minimisers of the Ginzburg–Landau functional minimise the Bethuel–Brezis–Hélein renormalised energy; refer to Section 2.2 for more details.

#### Remark 1.5

In the statement of this theorem, *d* stands for the standard exterior differential (for the flat metric), while $*$ is the Hodge operator [33, Section 3.3 p. 131] acting on 1-forms.

We see that the frame energy (1.7) coincides with the first term in (1.3). To obtain the second half of the Loewner energy involving

1.8 $$\begin{aligned} \int _{\mathbb {C}\setminus \overline{\mathbb {D}}}\left| \frac{g''(z)}{g'(z)}\right| ^2|dz|^2, \end{aligned}$$

we cannot easily use the Ginzburg–Landau equation to construct the moving frames since that would force us to work on the non-compact domain $\mathbb {C}\setminus \overline{\Omega }$. Using the inversion ${\mathfrak {i}}$ will not suffice either. If we choose the biholomorphic map $\widetilde{g}:\mathbb {D}\rightarrow {\mathfrak {i}}(\mathbb {C}\setminus \overline{\Omega })$ so that $\widetilde{g}={\mathfrak {i}}\circ g\circ {\mathfrak {i}}$, we have

$$\begin{aligned} \int _{\mathbb {D}}\left| \frac{\widetilde{g}''(z)}{\widetilde{g}'(z)}\right| ^2|dz|^2=\int _{\mathbb {C}\setminus \overline{\mathbb {D}}}\left| \frac{g''(z)}{g'(z)}-2\frac{g'(z)}{g(z)}+\frac{2}{z}\right| ^2|dz|^2, \end{aligned}$$

which is in general different from (1.8). To overcome this technicality, we work directly on $S^2$ to obtain a formula of the Loewner energy in terms of moving frames.

### Main Results

#### Theorem A

Let $\Gamma \subset S^2 \subset \mathbb R^3$ be a Weil–Petersson quasicircle, $\Omega _1,\Omega _2\subset S^2$ be the two disjoint open connected components of $S^2\setminus \Gamma$. Fix some $j=1,2$. Then, for any $p_j\in \Omega _j$, there exists harmonic moving frames $(\vec {u}_j,\vec {v}_j):\Omega _j\setminus \left\{ p_j\right\} \rightarrow U\Omega _j\times U\Omega _j$ such that the Cartan form $\omega _j=\langle \vec {u}_j,d\vec {v}_j\rangle$ admits the decomposition

1.9 $$\begin{aligned} \omega _j=*\,d\left( G_{\Omega _j}+\mu _j\right) , \end{aligned}$$

where $G_{\Omega _j}:\Omega _j\setminus \left\{ p_j\right\} \rightarrow \mathbb {R}$ is the Green’s function of the Laplacian $\Delta _{g_0}$ on $\Omega _j$ with Dirichlet boundary condition, and $\mu _j\in C^{\infty }(\Omega _j)$ satisfies

1.10 $$\begin{aligned} \left\{ \begin{aligned} -\Delta _{g_0}\mu _j&=1\qquad{} & {} \text {in}\;\,\Omega _j\\ \partial _{\nu }\mu _j&=k_{g_0}-\partial _{\nu }G_{\Omega _j}\qquad{} & {} \text {on}\;\,\partial \Omega _j, \end{aligned} \right. \end{aligned}$$

where $k_{g_0}$ is the geodesic curvature on $\Gamma =\partial \Omega _j$. Define the functional $\mathscr {E}$ *(*that we call the renormalised energy associated to the frames $(\vec {u}_1,\vec {v}_1)$ and $(\vec {u}_2,\vec {v}_2)$*)* by

**Fig. 1:**
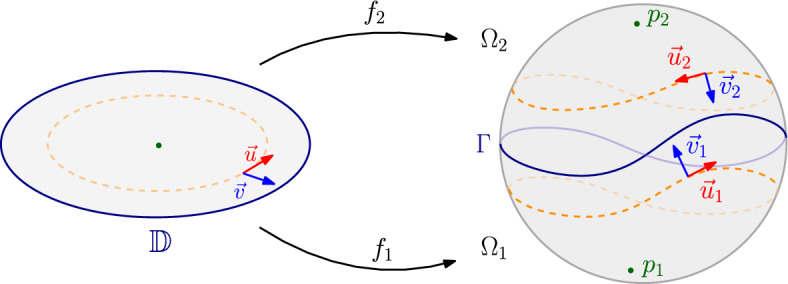
Harmonic moving frames on the sphere associated to a Weil–Petersson quasicircle

1.11 $$\begin{aligned} \mathscr {E}(\Gamma )&=\int _{\Omega _1}|d\mu _1|^2_{g_0}d\textrm{vol}_{g_0}+\int _{\Omega _2}|d\mu _2|^2_{g_0}d\textrm{vol}_{g_0}\nonumber \\&\quad +2\int _{\Omega _1}G_{\Omega _1}K_{g_0}d\textrm{vol}_{g_0}+2\int _{\Omega _2}G_{\Omega _2}K_{g_0}d\textrm{vol}_{g_0}+4\pi , \end{aligned}$$

where $K_{g_0}=1$ is the Gauss curvature of the metric $g_0$. Then there exists conformal maps $f_1:\mathbb {D}\rightarrow \Omega _1$ and (Fig. 1) $f_2:\mathbb {D}\rightarrow \Omega _2$ such that $f_1(0)=p_1$, $f_2(0)=p_2$ and

1.12 $$\begin{aligned} I^L(\Gamma )=\frac{1}{\pi }\mathscr {E}(\Gamma )+4\log |\nabla f_1(0)|+4\log |\nabla f_2(0)|-12\log (2)=\frac{1}{\pi }\mathscr {E}_0(\Gamma ). \end{aligned}$$

Notice that in the theorem, we define the functional

1.13 $$\begin{aligned} \mathscr {E}_0(\Gamma )&=\mathscr {E}(\Gamma )+4\pi \log |\nabla f_1(0)|+4\pi \log |\nabla f_2(0)|-12\pi \log (2)\nonumber \\&=\int _{\Omega _1}|d\mu _1|^2_{g_0}d\textrm{vol}_{g_0}+\int _{\Omega _2}|d\mu _2|^2_{g_0}d\textrm{vol}_{g_0}\nonumber \\&\quad +2\int _{\Omega _1}G_{\Omega _1}K_{g_0}d\textrm{vol}_{g_0}+2\int _{\Omega _2}G_{\Omega _2}K_{g_0}d\textrm{vol}_{g_0}\nonumber \\&\quad +4\pi \log |\nabla f_1(0)|+4\pi \log |\nabla f_2(0)|+4\pi -12\pi \log (2); \end{aligned}$$

that is, the quantity that appears in the Ginzburg–Landau type renormalised energy. Refer to Section 2.2 for more details.

#### Remark 1.10

1. As previously, *d* is the exterior derivative on $S^2$ and $*$ is the Hodge operator associated to the round metric $g_0$ on $S^2$. For more details on *d*, refer to Section 2.1.
2. In the theorem above, we wrote $U\Omega _j$ ($j=1,2$) for the unit tangent bundle. The function $\mu _j$, explicitly given by
  1.14 $$\begin{aligned} \mu _j=\frac{1}{2}\log \left( \frac{|\nabla f_j|^2}{2}\right) =\log |\nabla f_j|-\frac{1}{2}\log (2){,} \end{aligned}$$
  correspond to the conformal parameter of the conformal maps $f_1,f_2:\mathbb {D}\rightarrow S^2\subset \mathbb {R}^3$.
3. The constant term $4\pi$ in the definition of $\mathscr {E}$ is arranged so that $\mathscr {E}(S^1)=0$ (see Remark 3.8). Furthermore, the name renormalised energy is justified by the following identity
  1.15 $$\begin{aligned} \mathscr {E}(\Gamma )&=\int _{\Omega _1}\left( |d\vec {u}_1|^2_{g_0}+|d\vec {v}_1|_{g_0}^2-2|dG_{\Omega _1}|^2_{g_0}\right) d\textrm{vol}_{g_0}\nonumber \\&\quad +\int _{\Omega _2}\left( |d\vec {u}_2|^2_{g_0}+|d\vec {v}_2|_{g_0}^2-2|dG_{\Omega _2}|^2_{g_0}\right) d\textrm{vol}_{g_0}, \end{aligned}$$
  where no constant term is involved.
4. The solution (1.10) to the Dirichlet problem is unique, and so are the moving frames once the singularities $(p_1,p_2)\in \Omega _1\times \Omega _2$ are fixed. See Theorem 4.3 and 4.6. Notice that the geodesic curvature is understood in the distributional sense here (see Section 6.1 from the appendix for more details). For a definition of harmonic vector fields, refer to Lemma 4.1.

This theorem corresponds to Theorem 3.6 in the article for smooth curves and to Theorem 5.5 for general Weil–Petersson quasicircles. The general case follows essentially from the following result which can also be viewed as a restatement of Theorem A without any mention of moving frames:

#### Theorem B

(See Theorem 5.5) Let $\Gamma \subset S^2$ be a Weil–Petersson quasicircle and $\Omega _1,\Omega _2\subset S^2\setminus \Gamma$ be the two connected components of $S^2\setminus \Gamma$. For all conformal maps $f_1:\mathbb {D}\rightarrow \Omega _1$ and $f_2:\mathbb {D}\rightarrow \Omega _2$, we have

1.16 $$\begin{aligned} I^L(\Gamma )&=\frac{1}{\pi }\sum _{j=1}^{2}\bigg ( \int _{\mathbb {D}}|\nabla \log |\nabla f_j||^2|dz|^2+\int _{\mathbb {D}}\log |z||\nabla f_j|^2|dz|^2 \nonumber \\ {}&\quad +\text {Area}(\Omega _j) +4\pi \log |\nabla f_j(0)|\bigg ) -12\log (2) \end{aligned}$$

## Technical Background

Due to the variety of notions used in the article, we review some basic results related to the geometry of surfaces, harmonic maps, and moving frames.

### Zeta-Regularised Determinant of Elliptic Operators

Since the notion plays a central role in the proof of the main theorem, we remind basic notions related to determinants of the Laplacian, that would hold true for more general elliptic operators (provided that the spectrum is discrete and satisfies a Weyl Law). Let $(M^2,g)$ be a compact Riemannian surface with (smooth) boundary. Then, the Laplacian $\Delta _g$ is locally defined by

$$\begin{aligned} \Delta _g=\frac{1}{\sqrt{\det (g)}}\sum _{i,j=1}^{2}\partial _{x_i}\left( \sqrt{\det (g)}\partial _{x_j}\left( \,\cdot \,\right) \right) , \end{aligned}$$

if $g_{i,j}=g(\partial _{x_i},\partial _{x_j})$ are the coefficients of the metric, $\det (g)=g_{1,1}g_{2,2}-g_{1,2}^2$, and

2.1 $$\begin{aligned} g^{i,j}=(-1)^{i+j}\frac{g_{i+1,j+1}}{\det (g)} \end{aligned}$$

are the coefficient of the inverse matrix of $\left\{ g_{i,j}\right\} _{1\le i,j\le 2}\in \textrm{GL}_2(\mathbb {R})$ (notice that we used $\mathbb {Z}_2$ indices in formula (2.1)). If $d\textrm{vol}_g=\sqrt{\det (g)}\, dx_1\wedge dx_2$ is the associated volume form and $\langle \,\cdot \,,\,\cdot \,\rangle =\langle \,\cdot \,,\,\cdot \,\rangle _g$ is the scalar product associated to *g*, we define

$$\begin{aligned} L^2(M^2,d\textrm{vol}_g)=\left\{ u:M^2\rightarrow \mathbb {R}, \int _{M^2}|u|^2d\textrm{vol}_g<\infty \right\} , \end{aligned}$$

and the Dirichlet energy is defined for all smooth function $u:M^2\rightarrow \mathbb {R}$ by

$$\begin{aligned} E(u)=\frac{1}{2}\int _{M^2}|du|_g^2d\textrm{vol}_g, \end{aligned}$$

where, locally, we have

2.2 $$\begin{aligned} |du|_g^2=\langle du,du\rangle _g=\sum _{i,j=1}^2g^{i,j}\partial _{x_i}u\cdot \partial _{x_j}u. \end{aligned}$$

On can show that *E* extends to the Sobolev space

$$\begin{aligned} W^{1,2}(M^2)=\mathscr {D}'(M^2)\cap \left\{ u:u\in L^2(M^2,d\textrm{vol}_g), E(u)<\infty \right\} , \end{aligned}$$

that is a Hilbert space equipped with the norm

$$\begin{aligned} \Vert u\Vert _{\textrm{W}^{1,2}(M^2)}=\sqrt{\frac{1}{2}\int _{M^2}|u|^2d\text {vol}_g+E(u,g)}=\sqrt{\frac{1}{2}\int _{M^2}\left( |du|_g^2+|u|^2\right) d\text {vol}_g}. \end{aligned}$$

Notice that $W^{1,2}(M^2)$ does not depend on *g* by smoothness of this metric (and neither does $L^2(M^2,d\textrm{vol}_g)$). Here $\mathscr {D}'(M^2)$ stands for the classical space of distributions [46]. We also define

$$\begin{aligned} W^{1,2}_0(M^2)=\overline{C^{\infty }_c(M^2)}^{W^{1,2}} \end{aligned}$$

as the closure of compactly supported smooth function in the $W^{1,2}$ topology, which is the space of functions that “vanish on the boundary”—this informal interpretation can be made precise thanks to trace theory. Integrating by parts, one easily checks that for all $u\in W^{1,2}(M^2)$ and $\varphi \in W^{1,2}_0(M^2)$, the following identity is verified:

2.3 $$\begin{aligned} \int _{M^2}\langle du,d\varphi \rangle _gd\textrm{vol}_g=-\int _{M^2}\varphi \,\Delta _gu\,d\textrm{vol}_g. \end{aligned}$$

We say that $\lambda \in \mathbb {R}$ is a (Dirichlet) eigenvalue of $-\Delta _g$ if there exists an *eigenfunction* $u\in W^{1,2}_0(M^2)\setminus \left\{ 0\right\}$ such that

2.4 $$\begin{aligned} -\Delta _gu=\lambda \, u\qquad \text {in}\;\, M^2. \end{aligned}$$

By standard elliptic regularity, any eigenfunction *u* belongs to $C^{\infty }(M^2)$. Due to (2.3), we deduce that all eigenvalues are positive. Furthermore, the classical spectral theorem shows that the set of eigenvalues is discrete, and if we write the eigenvalues as an increasing sequence (counted with multiplicity) $\left\{ \lambda _k=\lambda _k(M^2,g)\right\} _{k\in \mathbb {N}}$, where

$$\begin{aligned} 0<\lambda _1(M^2,g)\le \lambda _2(M^2,g)\le \cdots \le \lambda _k(M^2,g)\rightarrow \infty , \end{aligned}$$

the Weyl law shows that

2.5 $$\begin{aligned} \lambda _k\underset{k\rightarrow \infty }{\simeq } \frac{4\pi }{\textrm{Vol}_g(M^2)}k. \end{aligned}$$

In particular, we can define the Minakshisundaram–Pleijel [36]; see also [3, Chapitre III, E.]) zeta function of $\Delta _g$ as

$$\begin{aligned} \zeta _{\Delta _g}(s)=\sum _{k=1}^{\infty }\frac{1}{\lambda _k^s} \end{aligned}$$

and (2.5) shows that $\zeta _{\Delta _g}(s)$ is holomorphic on $\mathbb {C}\cap \left\{ s:\textrm{Re}\,(s)>1\right\}$. In fact, the zeta function can be defined on any compact Riemannian manifold $(N^d,h)$ (with or without boundary) by the same formula, and it converges if $\textrm{Re}\,(s)>d/2$. For example, in the case of the circle $S^1$, the spectrum of the Laplacian is $\left\{ n^2\right\} _{n\in \mathbb {N}}$, and since each non-zero eigenvalue has multiplicity 2 (correspond to the two eigenfunctions $\cos (n\,\cdot \,)$ and $\sin (n\,\cdot \,)$), we get

2.6 $$\begin{aligned} \zeta _{\partial _{\theta }^2}(s)=2\sum _{n=1}^{\infty }\frac{1}{(n^2)^s}=2\,\zeta (2\,s), \end{aligned}$$

and we recover the standard zeta function of Riemann. As the classical zeta function, $\zeta _{\Delta _g}$ extends meromorphically to $\mathbb {C}$, and one can show that it is in fact holomorphic in a neighbourhood of $s=0$ [15, 16]. This is where the regularity of the boundary is needed to rewrite the zeta function using a Mellin transform and the precise asymptotic behaviour of the heat kernel at $t\rightarrow 0$. Therefore, one can define the (zeta-regularised) determinant of the Laplacian $\Delta _g$ as

2.7 $$\begin{aligned} \det \nolimits _{\hspace{-1.42262pt}\zeta }\left( -\Delta _{M^2,g}\right) =\exp \left( -\zeta _{\Delta _g}'(0)\right) . \end{aligned}$$

Notice that for all $s\in \mathbb {C}$ such that $\textrm{Re}\,(s)>1$

$$\begin{aligned} \zeta _{\Delta _g}'(s)=-\sum _{k=1}^{\infty }\frac{\log (\lambda _k)}{\lambda _k^s}. \end{aligned}$$

Therefore, we have the formal identity

$$\begin{aligned} \det \nolimits _{\hspace{-1.42262pt}\zeta }\left( -\Delta _{M^2,g}\right)&=\exp \left( -\zeta '_{\Delta _g}(0)\right) =\exp \left( \sum _{k=1}^{\infty }\frac{\log \left( \lambda _k\right) }{\lambda _k^s}\right) _{|s=0}\\&=\exp \left( \sum _{k=1}^{\infty }\log \left( \lambda _k\right) \right) =\prod _{k=1}^{\infty }\lambda _k, \end{aligned}$$

which is reminiscent of the Euler formula for the negative powers of the Riemann zeta function, and justifies the name of determinant of the Laplacian. For example, using the identity

$$\begin{aligned} \zeta '(0)=-\frac{1}{2}\log (2\pi ), \end{aligned}$$

we obtain by (2.6) the explicit value

$$\begin{aligned} \det \nolimits _{\hspace{-1.42262pt}\zeta }\left( -\Delta _{S^1,\partial _{\theta }^2}\right) =\exp \left( -4\,\zeta '(0)\right) =(2\pi )^2. \end{aligned}$$

In the case of $S^2$, the positive eigenvalues are given by $\left\{ \lambda _n=n(n+1)\right\} _{n\ge 1}$ and $\lambda _n$ has multiplicity $2n+1$ for all $n\ge 1$, which gives the formula

$$\begin{aligned} \zeta _{\Delta _{S^2}}(s)=\sum _{n=1}^{\infty }\frac{2n+1}{(n(n+1))^s}, \end{aligned}$$

that is indeed valid for all $s\in \mathbb {C}$ such that $\textrm{Re}\,(s)>1$. Let us mention that the values of determinants on the round spheres have been explicitly computed [42, 54]. For example, we have

$$\begin{aligned} \det \nolimits _{\hspace{-1.42262pt}\zeta }\left( -\Delta _{S^2}\right) =\Gamma _2\left( \frac{1}{2}\right) ^{\frac{8}{3}}2^{\frac{1}{9}}e^{\frac{1}{2}}=\exp \left( \frac{1}{2}-4\,\zeta '(-1)\right) =A^4e^{\frac{1}{6}}=3.195311\cdots , \end{aligned}$$

where $\Gamma _2$ is the Double Gamma function. Here, we used the identity

$$\begin{aligned} \zeta '(-1)=\frac{1}{12}-\log (A), \end{aligned}$$

where $A=1.282427\cdots$ is the Glaisher–Kinkelin constant [25, 26].

Notice that if $\varphi :(M^2,g)\rightarrow (N^2,h)$ is an isometry ($\varphi ^{*}h=g$), we have $\lambda _k(M^2,g)=\lambda _k(N^2,h)$ for all $k\ge 1$, which implies in particular that

2.8 $$\begin{aligned} \det \nolimits _{\hspace{-1.42262pt}\zeta }\left( -\Delta _{M^2,g}\right) =\det \nolimits _{\hspace{-1.42262pt}\zeta }\left( -\Delta _{N^2,h}\right) . \end{aligned}$$

For example, if $\pi :S^2\setminus \left\{ N\right\} \rightarrow \mathbb {C}$ is the stereographic projection and $\Omega \subset S^2\setminus \left\{ N\right\}$ is an open subset with smooth boundary $\partial \Omega \subset S^2\setminus \left\{ N\right\}$, we have

2.9 $$\begin{aligned} \det \nolimits _{\hspace{-1.42262pt}\zeta }\left( -\Delta _{\Omega ,g_0}\right) =\det \nolimits _{\hspace{-1.42262pt}\zeta }\left( -\Delta _{\pi (\Omega ),g_{\widehat{\mathbb {C}}}}\right) , \end{aligned}$$

where $g_0$ is the round metric on $S^2\subset \mathbb {R}^3$ and

$$\begin{aligned} g_{\widehat{\mathbb {C}}}=\frac{4|dz|^2}{(1+|z|^2)^2}. \end{aligned}$$

We will repeatedly use formulae (2.8) and (2.9) in Section 3 to transform determinants on the sphere into determinants on planar domains for which computations are easier. From the computational aspect, it appears that evaluating explicitly the Loewner energy of a given curve is quite challenging, and the formula expressing the Loewner energy as zeta-regularised determinants may indicate why. Let us conclude this section by listing important additional references, some of which were useful to write this section [23, 38, 41, 44, 47].

### Harmonic Maps and Renormalised Energy of Moving Frames

If $(M^2,g)$ is a smooth Riemannian surface and $N^n$ is a closed Riemannian manifold (that we assume to be isometrically embedded into $\mathbb {R}^d$), we say that $u:M^2\rightarrow N^n$ is a weak harmonic map provided that it satisfies in the distributional sense the equation

2.10 $$\begin{aligned} -\Delta _gu=A_u(\nabla u,\nabla u), \end{aligned}$$

where *A* is the second fundamental form of the embedding $\iota :N^n\hookrightarrow \mathbb {R}^d$ ([29, Lemme (1.2.4)]), and $\nabla$ is the Levi-Civita connection on $N^n$. Explicitly, if $x\in N^n$ there exists an open neighbourhood $U\subset N^n$ of *x* and smooth maps $(v_{n+1},\cdots ,v_d):U\rightarrow (\mathbb {R}^d)^{d-n}$ such that for all $y\in U$, $(v_{n+1}(y),\cdots ,v_{d}(y))$ is an orthonormal base of $(T_yN^n)^{\perp }$. Then, for all $(X,Y)\in T_yN^n\times T_yN^n$, we have

$$\begin{aligned} A_y(X,Y)=\left( \nabla _{X}Y\right) ^{\perp }=\sum _{i=n+1}^{d}\langle X,\nabla _{Y}v_i\rangle v_i. \end{aligned}$$

Therefore, (2.10) can be locally rewritten as

2.11 $$\begin{aligned} -\Delta _g u=\sum _{i,j=1}^2g^{i,j}A_u\left( \frac{\partial u}{\partial x_i},\frac{\partial u}{\partial x_j}\right) , \end{aligned}$$

where $\left\{ g^{i,j}\right\} _{1\le i,j\le 2}$ are defined in (2.1). Let us also remind that the second fundamental form *A* satisfies the following symmetry property: for all $y\in N^n$ and for all $X,Y\in T_yN^n$, $A_x(X,Y)=A_x(Y,X)$.

In the specific case where $N^n=S^{d-1}\subset \mathbb {R}^d$, it is easy to show that (2.10) is equivalent to

2.12 $$\begin{aligned} -\Delta _gu=|du|_g^2u, \end{aligned}$$

where $|du|_g^2$ was defined in (2.2). In this article, we will be concerned with the construction of unitary harmonic *vector fields* on $S^2$ with prescribed singularities, that should be seen as immediate generalisations of harmonic maps with values into $S^1$ for planar domains of $\mathbb {C}$. Let us recall the most relevant aspects of the classical Ginzburg–Landau theory. Let $\Omega \subset \mathbb {C}$ be a domain with smooth boundary (the theory makes sense for quasi-disks, but it would change little to the presentation). Due to degree reasons, if $h\in H^{1/2}(\partial \Omega ,S^1)$ has non-zero degree, it does not admit any extension in $W^{1,2}(\Omega ,S^1)$. In other words, we have

$$\begin{aligned} W^{1,2}_h(\Omega ,S^1)=W^{1,2}(\Omega ,S^1)\cap \left\{ u:u=h\;\,\text {on}\;\, \partial \Omega \right\} =\varnothing . \end{aligned}$$

Let us check this fact. One can define the degree of an $H^{1/2}$ map $h:\partial \Omega \rightarrow S^1$ as

$$\begin{aligned} \deg (h)=\frac{1}{2\pi }\int _{\partial \Omega }h\times dh=\frac{1}{2\pi }\int _{\partial \Omega }h_1\,dh_2-h_2\,dh_1. \end{aligned}$$

Notice that this integral makes sense (in the distributional sense) thanks to the $H^{1/2}/H^{-1/2}$ duality. The fact that for an $H^{1/2}$ map, the degree is an integer is due to L. Boutet de Monvel and O. Gabber [19]. If *h* admits an extension $u\in W^{1,2}(\Omega ,S^1)$, we, get by Stokes theorem,

2.13 $$\begin{aligned} \deg (h)=\frac{1}{2\pi }\int _{\Omega }d\left( u_1\,du_2-u_2\,du_1\right) =\frac{1}{\pi }\int _{\Omega }du_1\wedge du_2. \end{aligned}$$

However, since $|u|^2=1$, we have $\langle u,du\rangle =0$. Therefore, taking the wedge product of this equation, we find that

2.14 $$\begin{aligned} \left\{ \begin{aligned} 0&=(u_1\,du_1+u_2\,du_2)\wedge du_2=u_1\,du_1\wedge du_2\\ 0&=du_1\wedge (u_1\,du_1+u_2\,du_2)=u_2\,du_1\wedge du_2. \end{aligned}\right. \end{aligned}$$

Using once more that $|u|^2=1$, we deduce that $du_1\wedge du_2=0$, which finally implies by (2.13) that $\deg (h)=0$.

The Ginzburg–Landau theory consists in constructing harmonic $S^1$-valued maps with non-zero degree prescribed boundary values. The idea is to minimise the functional

$$\begin{aligned} E_{\varepsilon }(u)=\frac{1}{2}\int _{\Omega }|du|_g^2d\textrm{vol}_g+\frac{1}{4\varepsilon ^2}\int _{\Omega }(1-|u|^2)^2dx \end{aligned}$$

amongst maps $u:\Omega \rightarrow \mathbb {C}$ such that $u=h$ on $\partial \Omega$. By standard minimisation of $E_{\varepsilon }$, one finds a minimiser $u_{\varepsilon }:\Omega \rightarrow \mathbb {C}$ satisfying the system of equations

$$\begin{aligned} \left\{ \begin{aligned} -\Delta u_{\varepsilon }&=\frac{1}{\varepsilon ^2}(1-|u_{\varepsilon }|^2)u_{\varepsilon }\qquad \text {in}\;\,\Omega \\ u_{\varepsilon }&=h\qquad \text {on}\;\,\partial \Omega . \end{aligned}\right. \end{aligned}$$

By the previous analysis, we know that $\left\{ E_{\varepsilon }(u_{\varepsilon })\right\} _{\varepsilon >0}$ is unbounded as $\varepsilon \rightarrow 0$, lest a subsequence converge to a $W^{1,2}$ map that extends *h* (which is impossible by the previous discussion). The main difficulty of the theory is to extract a limit of $u_{\varepsilon }$ as $\varepsilon \rightarrow 0$, since we know that the limit cannot be regular. Furthermore, on can prove that if $\deg (h)=d\ge 1$, then

$$\begin{aligned} E_{\varepsilon }(u_{\varepsilon })\simeq \pi \,d\,\log \left( \frac{1}{\varepsilon }\right) . \end{aligned}$$

A remarkable feature of the minimisers of the Ginzburg–Landau functional is that one can extract a *renormalised energy* from the fine asymptotic behaviour of $E_{\varepsilon }(u_{\varepsilon })$. Before stating the main theorem, let us define the notion of renormalised energy according to Bethuel–Brezis–Hélein. Let $p_1,\cdots ,p_d\in \Omega$ be *d* distinct points. Define, for all $\varepsilon >0$ small enough,

$$\begin{aligned} \Omega _{\varepsilon }=\Omega \setminus \bigcup _{j=1}^{d}\overline{B}_{\varepsilon }(p_j), \end{aligned}$$

and let

$$\begin{aligned} \mathscr {E}_{\varepsilon }&=W^{1,2}(\Omega _{\varepsilon })\cap \left\{ v:v=h\;\, \text {on}\;\,\partial \Omega \;\,\text {and}\;\,\deg \left( v,\partial B_{\varepsilon }(p_j)\right) =1\;\, \text {for all}\;\,1\le j\le d\right\} . \end{aligned}$$

F. Bethuel, H. Brezis, and F. Hélein showed ([5, Theorem I.7]) that if $v_{\varepsilon }$ minimises the Dirichlet energy *E* on $\mathscr {E}_{\varepsilon }$, then, as $\varepsilon \rightarrow 0$, the following limit exists and is finite

2.15 $$\begin{aligned} W_h(p_1,\cdots ,p_d)=\lim _{\varepsilon \rightarrow 0}\left( \frac{1}{2}\int _{\Omega _{\varepsilon }}|\nabla v_{\varepsilon }|^2dx-\pi \,d\,\log \left( \frac{1}{\varepsilon }\right) \right) . \end{aligned}$$

This quantity is called the renormalised energy associated to *h* and $\left\{ p_1,\cdots ,p_d\right\}$ in the sense of Bethuel–Brezis–Hélein.

#### Theorem 2.1

(Bethuel–Brezis–Hélein [4, 5]) Let $\Omega \subset \mathbb {C}$ be a simply connected domain with smooth boundary $\gamma =\partial \Omega$, and $h\in C^{\infty }(\partial \Omega ,S^1)$ be a map of degree $\deg (h)=d\ge 1$. Then, there exists a sequence $\left\{ \varepsilon _n\right\} _{n\in \mathbb {N}}\subset (0,\infty )$ such that $\varepsilon _n\underset{n\rightarrow \infty }{\longrightarrow }0$ and *d* distinct points $p_1,\cdots ,p_d\in \Omega$ such that

2.16 $$\begin{aligned} \left\{ \begin{aligned}&u_{\varepsilon _n}\underset{n\rightarrow \infty }{\longrightarrow } u_{0}\quad \text {in}\;\,C^k_{\textrm{loc}}(\Omega \setminus \left\{ p_1,\cdots ,p_d\right\} )\qquad{} & {} \text {for all}\;\, k\in \mathbb {N}\\&u_{\varepsilon _n}\underset{n\rightarrow \infty }{\longrightarrow } u_{0}\quad \text {in}\;\, C^{1,\alpha }(\Omega )\qquad{} & {} \text {for all}\;\, \alpha <1. \end{aligned}\right. \end{aligned}$$

where $u_{0}\in W^{1,2}_{\textrm{loc}}(\Omega \setminus \left\{ p_1,\cdots ,p_d\right\} ,S^1)$ is a harmonic map with degree 1 singularities. More precisely, for all $1\le j\le d$, there exists $\theta _j\in \mathbb {R}$ such that

2.17 $$\begin{aligned} u_{0}(z)=e^{i\,\theta _j}\frac{z-p_j}{|z-p_j|}+O(|z-p_j|^2). \end{aligned}$$

Furthermore, the following limit exists

$$\begin{aligned} \lim _{\varepsilon \rightarrow 0}\left( E_{\varepsilon }(u_{\varepsilon })-\pi \,d\log \left( \frac{1}{\varepsilon }\right) \right) \end{aligned}$$

and there exists a universal constant $\gamma \in \mathbb {R}$ such that

2.18 $$\begin{aligned} \lim _{\varepsilon \rightarrow 0}\left( E_{\varepsilon }(u_{\varepsilon })-\pi \,d\log \left( \frac{1}{\varepsilon }\right) \right) =W_h(p_1,\cdots ,p_d)+d\,\gamma , \end{aligned}$$

where $W_h(p_1,\cdots ,p_d)$ is the *renormalised energy* associated to $\left\{ p_1,\cdots ,p_d\right\}$ and *h*, and the configuration $W_h(p_1,\cdots ,p_d)$ minimises $W_h$ amongst sets of distinct points $\left\{ q_1,\cdots ,q_d\right\} \subset \Omega$.

We see that $u_{0}$ fails to be in $W^{1,2}$ due to a singularity in

$$\begin{aligned} \frac{z}{|z|}\notin W^{1,2}(\mathbb {D}) \end{aligned}$$

near $p_j$ ($1\le j\le d$) according to (2.17). This terminology justifies the expression renormalised energy in the article, since we show that taking the sum of the Bethuel–Brezis–Hélein renormalised energies on each simply connected domain defined by a simple curve $\Gamma$ (with boundary conditions given by the unit tangent or normal) yields (up to a constant term) the Loewner energy of $\Gamma \subset S^2$. Using the special form of $u_{0}$ given by Laurain–Petrides’ result Theorem 1.3 (refer to [34] for detailed argument), let us show how to identify Bethuel–Brezis–Hélein renormalised energy in the case of a boundary data given by the unit tangent (that has degree 1) as the “first half”—up to a $2\pi \log |f'(0)|$ term—of the Universal Liouville action $S_1$ (see (1.3)), where we write

$$\begin{aligned} S_1(\gamma )&=\int _{\mathbb {D}}\left| \frac{f''(z)}{f'(z)}\right| ^2|dz|^2+\int _{\mathbb {C}\setminus \overline{\mathbb {D}}}\left| \frac{g''(z)}{g'(z)}\right| ^2|dz|^2\\ {}&\quad +4\pi \log |f'(0)|-4\pi \log |g'(\infty )|. \end{aligned}$$

Let $\Omega \subset \mathbb {C}$ be a simply connected domain and assume that $\gamma =\partial \Omega$ is a simple curve of finite Loewner energy. Let $\tau =h:\partial \Omega \rightarrow S^1$ be the unit tangent of $\partial \Omega$, and $\left\{ u_{\varepsilon }\right\} _{\varepsilon >0}$ be a sequence of minimisers of $E_{\varepsilon }$. Then, thanks to [34], as in [5, Section III.1], if $g(\theta )=e^{i\theta }$, define, for all $\varepsilon >0$,

$$\begin{aligned} I(\delta ,\varepsilon )=\inf _{w\in W^{1,2}_g(B(0,\delta ),\mathbb {R}^2)}\left\{ \frac{1}{2}\int _{B(0,\delta )}|\nabla w|^2dx+\frac{1}{4\varepsilon ^2}\int _{B(0,\delta )}(1-|w|^2)^2dx \right\} . \end{aligned}$$

Notice that we have the important invariance

2.19 $$\begin{aligned} I(\delta ,\varepsilon )=I\left( 1,\frac{\varepsilon }{\delta }\right) , \end{aligned}$$

which permits us to define, for all $\varepsilon >0$,

2.20 $$\begin{aligned} I(\varepsilon )=I(1,\varepsilon ). \end{aligned}$$

By [5], we know that

$$\begin{aligned} (0,\infty )&\rightarrow \mathbb {R}\\&t\mapsto I(\varepsilon )-\pi \log \left( \frac{1}{\varepsilon }\right) \end{aligned}$$

is a decreasing function, which implies in particular that the following limit exists and is finite:

$$\begin{aligned} \gamma =\lim \limits _{\varepsilon \rightarrow 0}\left( I(\varepsilon )-\pi \log \left( \frac{1}{\varepsilon }\right) \right) <\infty . \end{aligned}$$

This is the constant $\gamma$ appearing in (2.18). Furthermore, by Theorem IX.3 of [5] (quoted above in Theorem 2.1; see equation (2.15)), we have

2.21 $$\begin{aligned}&E_{\varepsilon }(u_{\varepsilon })=\pi \log \left( \frac{1}{\varepsilon }\right) +W_{\tau }(p_1)+\gamma +o_{\varepsilon }(1), \end{aligned}$$

where $W_{\tau }(p_1)$ is the renormalised energy defined in (2.15). Up to a translation of $\Omega$, we can assume that $p_1=0$. We actually only need to know that the limit exists since we will use another expression of the renormalised energy. Thanks to [5, I.8, VI.2 and VII.1], we have

2.22 $$\begin{aligned} \frac{1}{2}\int _{\Omega \setminus B(0,\varepsilon )}|\nabla u_0|^2dx=\pi \log \left( \frac{1}{\varepsilon }\right) +W_{\tau }(0)+O(\varepsilon ^2). \end{aligned}$$

For all $p\in \Omega$, let $G_p:\Omega \rightarrow \mathbb {R}$ be the Green’s function of the Laplacian on $\Omega$ such that

$$\begin{aligned} \left\{ \begin{aligned}\Delta G_p&=2\pi \delta _{p}\qquad {}{} & {} \text{ in }\;\, \Omega \\ G_p&=0\qquad {}{} & {} \text{ on }\;\,\partial \Omega . \end{aligned} \right. \end{aligned}$$

Now, we know by (2.17) that for $\delta >0$ small enough, we have the expansion

$$\begin{aligned} u_0(z)=\frac{z}{|z|}e^{iH(z)}\qquad \text{ for } \text{ all }\;\, z\in B(0,\delta ), \end{aligned}$$

where $H:B(0,\delta )\rightarrow \mathbb {R}$ is a smooth harmonic function. In particular, we deduce that

$$\begin{aligned} |\nabla u_0(z)|^2=\frac{1}{|z|^2}+O\left( \frac{1}{|z|}\right) . \end{aligned}$$

Therefore, we have the estimate

2.23 $$\begin{aligned} |\nabla u_0|^2-|\nabla G_0|^2=O\left( \frac{1}{|z|}\right) \in L^1(\Omega ). \end{aligned}$$

Now, let $G_{\mathbb {D}}=\log |x|$ be the Dirichlet Green’s function of the unit disk $\mathbb {D}\subset \mathbb {C}$, and $f:\mathbb {D}\rightarrow \Omega$ be a conformal map such that $f(0)=0$. By conformal invariance of the Dirichlet energy, we have

$$\begin{aligned} \left\{ \begin{aligned} \Delta (G_{\mathbb {D}}\circ f^{-1})&=2\pi \delta _{0}\qquad \text {in}\;\,{} & {} \Omega \\ G_0\circ f^{-1}&=0\qquad \text {on}\;\,{} & {} \partial \Omega , \end{aligned} \right. \end{aligned}$$

which shows that $G_0=G_{\mathbb {D}}\circ f^{-1}$. Now, for all $\lambda >0$, we have for all $c\in \mathbb {R}$ and $\delta >0$ small enough

2.24 $$\begin{aligned}&\int _{B(0,1)\setminus \overline{B}(0,\lambda \,\delta (1+c\, \delta ))}|\nabla \log |x||^2dx=-2\pi \log (\lambda \delta (1+c\, \delta ))\nonumber \\&\quad =2\pi \log \left( \frac{1}{ \delta }\right) -2\pi \log (\lambda )+O(\delta ). \end{aligned}$$

In particular, since we have the Taylor expansion $f(z)=f'(0)z+O(|z|^2)$, we deduce that there exist $c>0$ such that

$$\begin{aligned}&B(0,1)\setminus \overline{B}\left( 0,|f'(0)|^{-1}\delta (1-c\ \delta )\right) \subset f^{-1}\left( \Omega \setminus \overline{B(0,\delta )}\right) \\&\quad \subset B(0,1)\setminus \overline{B}(0,|f'(0)|^{-1}\delta (1+c\,\delta )) \end{aligned}$$

and by (2.24) that

$$\begin{aligned} \frac{1}{2}\int _{B(0,1)\setminus f^{-1}(\overline{B}(0, \delta ))}|\nabla G_{\mathbb {D}}|^2dx=\pi \log \left( \frac{1}{ \delta }\right) +\pi \log |f'(0)|+O( \delta ). \end{aligned}$$

Using the conformal invariance of the Dirichlet energy, we finally deduce that

2.25 $$\begin{aligned} \frac{1}{2}\int _{\Omega \setminus \overline{B}(0, \delta )}|\nabla G_0|^2dx=\pi \log \left( \frac{1}{ \delta }\right) +\pi \log |f'(0)|+O( \delta ). \end{aligned}$$

Finally, by (2.22), (2.23) and (2.25), we deduce that

2.26 $$\begin{aligned} W_{\tau }(0)&=\lim \limits _{ \delta \rightarrow 0}\left\{ \frac{1}{2}\int _{\Omega \setminus B(0, \delta )}\left( |\nabla u_0|^2-|\nabla G_0)|^2\right) dx\right\} +\pi \log |f'(0)|\nonumber \\&=\frac{1}{2}\int _{\Omega }\left( |\nabla \vec {u}_0|^2-|\nabla G_0|^2\right) dx+\pi \log |f'(0)|. \end{aligned}$$

Likewise, if $\chi :\partial \Omega \rightarrow S^1$ is the unit normal, since the minimiser $v_{\varepsilon }$ of $E_{\varepsilon }$ for the Dirichlet boundary data $\chi$ is a constant rotation by $-\pi /2$ of $u_{\varepsilon }$, we deduce that

2.27 $$\begin{aligned} E_{\varepsilon }(u_{\varepsilon })+E_{\varepsilon }(v_{\varepsilon })&=2\pi \log \left( \frac{1}{\varepsilon }\right) +\frac{1}{2}\int _{\Omega }\left( |\nabla u_0|^2+|\nabla v_0|^2-2|\nabla G_0|^2\right) dx\nonumber \\&\quad +2\pi \log |f'(0)|+2\gamma . \end{aligned}$$

Now, recall that by Theorem 1.3, we have

2.28 $$\begin{aligned} \frac{1}{2}\int _{\Omega }\left( |\nabla u_0|^2+|\nabla v_0|^2-2|\nabla G_0|^2\right) dx&= \int _{\Omega }|\omega -*\, dG_{\Omega }|^2dx\nonumber \\&=\int _{\mathbb {D}}\left| \frac{f''(z)}{f'(z)}\right| ^2|dz|^2. \end{aligned}$$

Notice that in this identity, $G_{\Omega }=G_0$. Finally, we deduce by (2.27) and (2.28), we have

2.29 $$\begin{aligned} E_{\varepsilon }(u_{\varepsilon })+E_{\varepsilon }(v_{\varepsilon })&=2\pi \log \left( \frac{1}{\varepsilon }\right) +\int _{\mathbb {D}}\left| \frac{f''(z)}{f'(z)}\right| ^2|dz|^2\nonumber \\&\quad +2\pi \log |f'(0)|+2\gamma +o_{\varepsilon }(1). \end{aligned}$$

Taking the limit as $\varepsilon \rightarrow 0$, we find the identity

2.30 $$\begin{aligned} \int _{\mathbb {D}}\left| \frac{f''(z)}{f'(z)}\right| ^2|dz|^2+2\pi \log |f'(0)|&=\lim _{\varepsilon \rightarrow 0}\left( E_{\varepsilon }(u_{\varepsilon })+E_{\varepsilon }(v_{\varepsilon })-2\pi \log \left( \frac{1}{\varepsilon }\right) \right) -2\gamma , \end{aligned}$$

which indeed corresponds to two terms in (1.3) (up to a $2\pi \log |f'(0)|$ factor). Now, comparing this formula with (1.15) and (2.29), one may justify the terminology renormalised energy used in the present article. The major difference with [34] is that we do not use the Ginzburg–Landau equation to construct the relevant moving frames but construct them via complex analysis methods. The main difficulty is to show that the new formula for the Loewner energy holds true for Weil–Petersson curves, which forces us to make a detour via another functional that possesses the right compactness properties.

Here, we see that the first two terms of the expression of the Loewner energy are almost equal to (up to this $2\pi \log |f'(0)|$ factor mentioned above) the renormalised energy of the moving frame $(u_0,v_0):\Omega \rightarrow S^1\times S^1$, where by moving frame, we simply mean that $\langle u_0,v_0\rangle =0$ identically. In this article, we also consider moving frames on the sphere. In this case if $\Omega \subset S^2$, we say that a couple of unit *vector fields* $(u_0,v_0):\Omega \rightarrow S^2\times S^2$ is a moving frame if $\langle u_0,v_0\rangle =0$ identically, where $\langle \,\cdot \,,\,\cdot \,\rangle$ is the restriction of the scalar product of $\mathbb {R}^3$ on $S^2$. In general, moving frames are sections of the unit bundle of a given Riemannian manifold, but we will not need any general definition in this article (refer to [29, Chapitre 4] for more details).

## Moving Frame Energy via Zeta-Regularised Determinants for Smooth Curves

The following expression of the Loewner energy will prove to be crucial in this section:

### Theorem 3.1

(Y. Wang [59]) Let $\alpha \in C^\infty (S^2, \mathbb {R})$, $g=e^{2\alpha }g_0$ be a metric conformally equivalent to the spherical metric $g_0$ of $S^2$, and $\Gamma \subset S^2$ be a simple smooth curve. Let $\Omega _1,\Omega _2\subset S^2$ be the two disjoint open connected components of $S^2\setminus \Gamma$. Then we have

3.1 $$\begin{aligned} I^L(\Gamma )=12\,\log \frac{\det _{\zeta }(-\Delta _{S^2_-,g})\det _{\zeta }(-\Delta _{S^2_+,g})}{\det _{\zeta }(-\Delta _{\Omega _1,g})\det _{\zeta }(-\Delta _{\Omega _2,g})}, \end{aligned}$$

where $S_-^2$ *(*resp., $S^2_+$*)* is the southern hemisphere *(*resp., the northern hemisphere*)*.

We now use the formula (3.1) expressing the Loewner energy in terms of zeta-regularised determinants to link the Loewner energy to the renormalised energy of moving frames on $S^2$. First, let $g_0=g_{S^2}$ be the standard round metric on $S^2$. Let $\Gamma \subset S^2$ be a simple *smooth*^1^ curve, and let $\Omega _1,\Omega _2\subset S^2$ the two disjoint open connected components of $S^2\setminus \Gamma$. Since we are working on a curved manifold, we cannot directly use the result of [34] to construct moving frames with the Ginzburg–Landau method. However, we will construct them directly in Section 4 (see Lemma 4.1 and Theorem 4.3). Therefore, let us assume that $(\vec {u}_1,\vec {v}_1):\Omega _1\setminus \left\{ p_1\right\} \rightarrow US^2\times US^2$ are harmonic vector fields such that $\vec {u}_1=\tau$ on $\partial \Omega _1=\Gamma$ (where $\tau$ is the unit tangent on $\Gamma$), and the 1-form $\omega =\langle \vec {u}_{1},d\vec {v}_{1}\rangle$ satisfies

3.2 $$\begin{aligned} \omega =*\,d\,\left( G_{\Omega _1}+\mu _1\right) \qquad \text {in}\;\, \mathscr {D}'(\Omega _1) \end{aligned}$$

where $G_{\Omega _{1}}:\Omega _1\setminus \left\{ p_1\right\} \rightarrow \mathbb {R}$ is the Green’s function for the Laplacian on $\Omega _1\setminus \left\{ p_1\right\}$ with Dirichlet boundary condition. Namely, $G_{\Omega _1}$ satisfies

3.3 $$\begin{aligned} \left\{ \begin{aligned} \Delta _{g_0}G_{\Omega _1}&=2\pi \delta _{p_1}\qquad{} & {} \text {in}\;\,\mathscr {D}'(\Omega _1)\\ G_{\Omega _1}&=0\qquad{} & {} \text {on}\;\,\partial \Omega _1, \end{aligned} \right. \end{aligned}$$

and $\mu _1:\Omega _1\rightarrow \mathbb {R}$ is a smooth function satisfying

3.4 $$\begin{aligned} \left\{ \begin{aligned} -\Delta _{g_0}\mu _1&=1\qquad{} & {} \text {in}\;\,\Omega _1\\ \partial _{\nu }\mu _1&=k_{g_0}-\partial _{\nu }G_{\Omega _1}\qquad{} & {} \text {on}\;\,\partial \Omega _1. \end{aligned} \right. \end{aligned}$$

where $k_{g_0}$ is the geodesic curvature with respect to the round metric $g_0$, and the normal derivative is taken with respect to the $g_0$.

To fix notations, we recall the following result:

### Theorem 3.2

([32], see also, [31, 57]) Let $\Omega \subset \mathbb {C}$ *(*resp., $\Omega \subset S^2$*)* be a bounded simply connected domain such that $\partial \Omega$ is chord-arc, and let $g_0$ be the flat metric on $\Omega$ *(*resp., $g_0$ be the round metric on $S^2$*)*. Then for all $p\in \Omega$, there exists a unique Green’s function $G_{\Omega ,p}\in C^{\infty }(\Omega \setminus \left\{ p\right\} ,\mathbb {R})$ with Dirichlet boundary condition. Furthermore, for every $h\in H^{1/2}(\partial \Omega ,\mathbb {R})$, there exists a unique function $u\in W^{1,2}(\Omega ,\mathbb {R})$ such that

$$\begin{aligned} \left\{ \begin{aligned} \Delta _{g_0}u&=0\qquad{} & {} \text {in}\;\,\Omega \\ u&=h\qquad{} & {} \text {on}\;\,\partial \Omega . \end{aligned}\right. \end{aligned}$$

Whenever it is clear from context, we will write $G_{\Omega _1}$ for $G_{\Omega _1,p_1}$.

### Remark 3.3

The existence of a Green’s function follows from its conformal invariance and the uniformisation theorem. Indeed, if $\Omega$ is a Jordan domain, and $f:\mathbb {D}\rightarrow \Omega$ is a biholomorphic map such that $f(0)=p$, and $G_{\mathbb {D},0}=\log |z|$, then $G_{\Omega ,p}=G_{\mathbb {D},0}\circ f^{-1}$. We assume that $\partial \Omega$ is chord-arc so that the trace theorems apply as in [32, 57]. The passage from $\mathbb {C}$ to $S^2$ is easy using a stereographic projection and the conformal invariance of Green’s functions.

Now, following Proposition 5.1 of [34], it is not hard to see that their proof using the Froebenius theorem also works for domains of the sphere, and we get a conformal diffeomorphism ${\varphi }:(-\infty ,0)\times \partial B(0,\rho )\rightarrow \Omega _1\setminus \left\{ p_1\right\}$ for some $\rho > 0$ such that

$$\begin{aligned} \partial _{s}{\varphi }(s,\theta )&=e^{G_{\Omega _1}\circ {\varphi }+\mu _1\circ {\varphi }}\vec {v}_{1}\circ {\varphi }\\ \partial _{\theta }{\varphi }(s,\theta )&=e^{G_{\Omega _1}\circ {\varphi }+\mu _1\circ {\varphi }}\vec {u}_{1}\circ {\varphi }. \end{aligned}$$

Notice that the Proposition 5.1 of [34] gives a privileged $p_1\in \Omega _1$, but we will show in Theorem 4.3 that $p_1$ can be taken arbitrarily (see also Theorem 5.5). However, the proof works for an arbitrary harmonic moving frame whose Cartan form admits an expansion as in (3.2) where $\mu _1$ solves (3.4). Since $\mu _1$ is defined up to an additive constant, we can assume that $\rho =1$ in the following. We define the conformal map $f_1:\mathbb {D}\rightarrow \Omega _1$ using the polar coordinates by

$$\begin{aligned} f_1(r,\theta )={\varphi }(\log (r),\theta ), \end{aligned}$$

we can continuously extend $f_1$ at $z=0$ such that $f_1(0)=p_1$.

Now we relate $\mu _1$ to $f_1$. Since $f_{1}$ is conformal, the function $G=G_{\Omega _1}\circ f_{1}:\mathbb {D}\setminus \left\{ 0\right\} \rightarrow \mathbb {R}$ is harmonic on $\mathbb {D}\setminus \left\{ 0\right\}$, satisfies $G=0$ on $\partial \mathbb {D}$, so by (3.3), we deduce that

$$\begin{aligned} G=G_{\mathbb {D},{0}}=\log |z|. \end{aligned}$$

Therefore, we have

3.5 $$\begin{aligned} \left\{ \begin{aligned} \partial _r f_1&=\frac{1}{r}\partial _{s}{\varphi }(\log (r),\theta )=\frac{1}{r}e^{\log (r)+\mu _1\circ f_1}{\vec {v}}_1\circ f_1=e^{\mu _1\circ f_1}\vec {v}_1\circ f_1\\ \frac{1}{r}\partial _{\theta }f_1&=\frac{1}{r}\partial _{\theta }{\varphi }(\log (r),\theta )=e^{\mu _1\circ f_1}\vec {u}_1\circ f_1. \end{aligned}\right. \end{aligned}$$

Since $|\vec {u}_1|=|\vec {v}_1|=1$, and $\langle \vec {u}_{1},\vec {v}_{1}\rangle =0$, we deduce that

$$\begin{aligned}&|\partial _rf_1|^2=\frac{1}{r^2}\left| \partial _{\theta }f_1\right| ^2=e^{2\mu _1\circ f_1}\\&\langle \partial _rf_1,\partial _{\theta }f_1\rangle =0, \end{aligned}$$

which shows that the conformal parameter of *f* is

$$\begin{aligned} \frac{1}{2}|\nabla f_1|^2 = {\frac{1}{2} \left( |\partial _rf_1|^2+ \frac{1}{r^2}\left| \partial _{\theta }f_1\right| ^2\right) }=e^{2\mu _1\circ f_1}, \end{aligned}$$

which implies that

3.6 $$\begin{aligned} \mu _1=\log |\nabla f_1|\circ f_1^{-1}-\frac{1}{2}\log (2). \end{aligned}$$

In particular, we have

3.7 $$\begin{aligned} \mu _1(p_1)=\log |\nabla f_1(0)|-\frac{1}{2}\log (2), \end{aligned}$$

where $p_1\in \Omega _1$ is the singularity of the moving frame $(\vec {u}_1,\vec {v}_1):\Omega _1\setminus \left\{ p_1\right\} \rightarrow US^2\times U S^2$.

We can relate the change of metric by $f_1$ to $\mu _1$ as follows. If $\iota :\Omega _1\subset S^2\hookrightarrow \mathbb {R}^3$ is the inclusion map, we have ${{g_{0}}_{|\Omega _1}} =\iota ^{*}g_{\mathbb {R}^3}$.

As $f_{1}$ is conformal, we have

3.8 $$\begin{aligned} f_1^{*}{g_{0}}_{|\Omega _1}&=f_1^{*}\iota ^{*}g_{\mathbb {R}^3}=(\iota \circ f_1)^{*}g_{\mathbb {R}^3}= {\frac{1}{2}}|\nabla f_1(z)|^2|dz|^2=e^{2\mu _1\circ f_1(z)}|dz|^2\nonumber \\&=e^{2\mu _1\circ f_1(z)-2\psi (z )}\frac{4|dz|^2}{(1+|z|^2)^2}\nonumber \\&=e^{2\mu _1\circ f_1-2\psi }(({\pi ^{-1}})^{*}g_{0})|_{\mathbb {D}}, \end{aligned}$$

where

$$\begin{aligned} \psi (z)=\log \left( \frac{2}{1+|z|^2}\right) , \end{aligned}$$

and $\pi ^{-1}:\mathbb {C}\rightarrow S^2\setminus \left\{ N\right\}$ is the inverse stereographic projection. Writing for simplicity

$$\begin{aligned} g_{\mathbb {D}}=\frac{4|dz|^2}{(1+|z|^2)^2}=e^{2\psi (z)}|dz|^2 = (\pi ^{-1})^*g_0|_{\mathbb D}, \end{aligned}$$

we deduce by (3.8) that

$$\begin{aligned} {g_{0}}_{|\Omega _1}=(f_1\circ f_1^{-1})^{*}(g_{0|\Omega _1})=(f_1^{-1})^{*}f_1^{*}{g_{0}}_{|\Omega _1}= e^{2\mu _1-2\psi \circ f_1^{-1}}(f_1^{-1})^{*}(g_{\mathbb {D}}), \end{aligned}$$

so that (by an abuse of notation for the last identity)

3.9 $$\begin{aligned} (f_1^{-1})^{*}(g_{\mathbb {D}})=e^{-2\mu _{1}+2\psi \circ f_1^{-1}}{g_{0}}_{|\Omega _1}=e^{2\alpha _1}{g_{0}}_{|\Omega _{1}} \end{aligned}$$

where

$$\begin{aligned} \alpha _1(z)=-\mu _{1}(z)+\psi {\circ } f_1^{-1}(z). \end{aligned}$$

### Remark 3.4

To summarize, the above discussion shows that the moving frame $(\vec {u}_1, \vec {v}_1)$ satisfying the boundary condition $\vec {u}_1 = \tau$ on $\Gamma$, (3.2), and (3.4) is tightly related to a conformal map $f_1 : \mathbb D \rightarrow \Omega _1$ using Froebenius theorem as in [34], in the way that the moving frame satisfies (3.5). However, we can start directly with any conformal map $f_1$ and (3.5) gives a moving frame $(\vec {u}_1, \vec {v}_1)$ which satisfies (3.2) and (3.4). This is the approach we take in Section 4 which allows us to relax the regularity assumption of $\partial \Omega _1=\Gamma$.

### Definition 3.5

Define the open subsets $S_+^2,S^2_-\subset S^2$ by

$$\begin{aligned} S_+^2&=S^2\cap \left\{ (x,y,z) {\in \mathbb R^3}:z>0\right\} \\ S_-^2&=S^2\cap \left\{ (x,y,z) {\in \mathbb R^3}:z<0\right\} . \end{aligned}$$

### Theorem 3.6

Let $\Gamma \subset S^2$ be a smooth Jordan curve, and let $\Omega _1,\Omega _2\subset S^2$ the two disjoint open connected components of $S^2\setminus \Gamma$. Fix some $j=1,2$. Then, for all $p_j\in \Omega _j$ and for all harmonic moving frames $(\vec {u}_j,\vec {v}_j):\Omega _j\setminus \left\{ p_j\right\} \rightarrow U\Omega _j\times U\Omega _j$, assume that the Cartan form $\omega _j=\langle \vec {u}_j,d\vec {v}_j\rangle$ admits the decomposition

$$\begin{aligned} \omega _j=*\,d\left( G_{\Omega _j}+\mu _j\right) , \end{aligned}$$

where $G_{\Omega _j}:\Omega _j\setminus \left\{ p_j\right\} \rightarrow \mathbb {R}$ is the Green’s function of the Laplacian $\Delta _{g_0}$ on $\Omega _j$ with Dirichlet boundary condition, and $\mu _j\in C^{\infty }(\Omega _j)$ satisfies

3.10 $$\begin{aligned} \left\{ \begin{aligned} -\Delta _{g_0}\mu _j&=1\qquad{} & {} \text {in}\;\,\Omega _j\\ \partial _{\nu }\mu _j&=k_{g_0}-\partial _{\nu }G_{\Omega _j}\qquad{} & {} \text {on}\;\,\partial \Omega _j, \end{aligned} \right. \end{aligned}$$

where $k_{g_0}$ is the geodesic curvature on $\Gamma =\partial \Omega _j$. Define the functional $\mathscr {E}$ *(*that we call the renormalised energy associated to the frames $(\vec {u}_1,\vec {v}_1)$ and $(\vec {u}_2,\vec {v}_2)$*)* by

$$\begin{aligned} \mathscr {E}(\Gamma )&=\int _{\Omega _1}|d\mu _1|^2_{g_0}d\textrm{vol}_{g_0}+\int _{\Omega _2}|d\mu _2|^2_{g_0}d\textrm{vol}_{g_0}+2\int _{\Omega _1}G_{\Omega _1}K_{g_0}d\textrm{vol}_{g_0}\\&\quad +2\int _{\Omega _2}G_{\Omega _2}K_{g_0}d\textrm{vol}_{g_0}+4\pi . \end{aligned}$$

Then, there exists conformal maps $f_1:\mathbb {D}\rightarrow \Omega _1$ and $f_2:\mathbb {D}\rightarrow \Omega _2$ such that $f_1(0)=p_1$, $f_2(0)=p_2$ and

$$\begin{aligned} I^L(\Gamma )=\frac{1}{\pi }\mathscr {E}(\Gamma )+4\log |\nabla f_1(0)|+4\log |\nabla f_2(0)|-12\log (2)=\frac{1}{\pi }\mathscr {E}_0(\Gamma ). \end{aligned}$$

### Remark 3.7

If ${W}_j$ ($j=1,2$) is the renormalised energy in the sense of Bethuel–Brezis–Hélein (see Section 2.2 in the planar case, especially equations (2.15) and (2.30)) associated to the moving frame $(\vec {u}_j,\vec {v}_j)$—or more precisely, to its boundary data—(see [5] and [34]), we have

$$\begin{aligned} {W}_1+{W}_2=\mathscr {E}(\Gamma )+2\pi \log |\nabla f_1(0)|+2\pi \log |\nabla f_2(0)|, \end{aligned}$$

since $$\omega _1-*\,dG_{\Omega _1}=*\, d\mu _1$$.

### Proof of Theorem 3.6

If $\pi ^{-1}:\mathbb {C}\rightarrow S^2\setminus \left\{ N\right\}$ is the inverse stereographic projection,

$$\begin{aligned} g_{\mathbb {D}}=\frac{4|dz|^2}{(1+|z|^2)^2}=e^{2\psi (z)}|dz|^2, \end{aligned}$$

and $S^2_-$ is the southern hemisphere, we deduce that

3.11 $$\begin{aligned} \det \nolimits _{\hspace{-1.42262pt}\zeta }(-\Delta _{S^2_-,g_0})&=\det \nolimits _{\hspace{-1.42262pt}\zeta }(-\Delta _{\mathbb {D},\pi ^{*}g_0})=\det \nolimits _{\hspace{-1.42262pt}\zeta }(-\Delta _{\mathbb {D},g_{\mathbb {D}}}) =\det \nolimits _{\hspace{-1.42262pt}\zeta }(-\Delta _{\Omega _1,\varphi ^{*}g_{\mathbb {D}}})\nonumber \\&=\det \nolimits _{\hspace{-1.42262pt}\zeta }(-\Delta _{\Omega _1,g_{1}}), \end{aligned}$$

and by the Alvarez–Polyakov formula (see (1.17) of [41]) and (3.9), we have

3.12 $$\begin{aligned}&\log \det \nolimits _{\hspace{-1.42262pt}\zeta }(-\Delta _{S^2_-,g_0})-\log \det \nolimits _{\hspace{-1.42262pt}\zeta }(-\Delta _{\Omega _1,g_0})\nonumber \\ {}&\quad =\log \det \nolimits _{\hspace{-1.42262pt}\zeta }(-\Delta _{\Omega _1,g_1})-\log \det \nolimits _{\hspace{-1.42262pt}\zeta }(-\Delta _{\Omega _1,g_0})\nonumber \\ {}&\quad =-\frac{1}{12\pi }\bigg \{\int _{\Omega _1}|d\alpha _1|_{g_0}^2d\text {vol}_{g_0}+2\int _{\Omega _1}K_{g_0}\alpha _1\, d\text {vol}_{g_0}\nonumber \\ {}&\qquad +2\int _{\Gamma }k_{g_0}\alpha _1\, d\mathscr {H}^1_{g_0}+3\int _{\Gamma }\partial _{\nu }\alpha _1\, d\mathscr {H}^1_{g_0}\bigg \}\nonumber \\ {}&\quad =-\frac{1}{12\pi }\bigg \{\int _{\Omega _1}|d(-\mu _1+\theta _1)|_{g_0}^2d\text {vol}_{g_0}+2\int _{\Omega _1}K_{g_0}(-\mu _1+\theta _1)d\text {vol}_{g_0}\nonumber \\ {}&\qquad +2\int _{\Gamma }k_{g_0}(-\mu _1+\theta _1)d\mathscr {H}^1_{g_0} +3\int _{\Gamma }\partial _{\nu }(-\mu _1+\theta _1)d\mathscr {H}^1_{g_0}\bigg \}. \end{aligned}$$

Notice that the first identity follows from the conformal invariance of the zeta-regularised determinants which implies identity (3.11). If we choose $\Gamma$ to be oriented as $\partial \Omega _1$, and

$$\begin{aligned} \theta _1=\psi \circ f^{-1}_1. \end{aligned}$$

Therefore, using subscripts with evident notations, we deduce by Theorem 3.1 with $g=g_0$ and formula (3.12) (see the proof of Theorem 7.3 from [59] for more details) applied twice that

3.13 $$\begin{aligned}&-\pi \,I^L(\Gamma )=-12\pi \,\log \frac{\det _{\zeta }(-\Delta _{S^2_-,g_0})\det _{\zeta }(-\Delta _{S^2_+,g_0})}{\det _{\zeta }(-\Delta _{\Omega _1,g_0})\det _{\zeta }(-\Delta _{\Omega _2,g_0})}\nonumber \\ {}&\quad =-12\pi \,\log \frac{\det _{\zeta }(-\Delta _{\Omega _1,g_1})\det _{\zeta }(-\Delta _{\Omega _2,g_2})}{\det _{\zeta }(-\Delta _{\Omega _1,g_0})\det _{\zeta }(-\Delta _{\Omega _2,g_0})}\nonumber \\ {}&\quad =\int _{\Omega _1}|d(-\mu _1+\theta _1)|_{g_0}^2d\text {vol}_{g_0}+2\int _{\Omega _1}K_{g_0}(-\mu _1+\theta _1)d\text {vol}_{g_0}\nonumber \\ {}&\qquad +2\int _{\partial \Omega _1}k_{g_0}(-\mu _1+\theta _1)d\mathscr {H}^1_{g_0} +3\int _{\partial \Omega _1}\partial _{\nu }(-\mu _1+\theta _1)d\mathscr {H}^1_{g_0}\nonumber \\ {}&\qquad +\int _{\Omega _2}|d(-\mu _2+\theta _2)|_{g_0}^2d\text {vol}_{g_0}+2\int _{\Omega _2}K_{g_0}(-\mu _2+\theta _2)d\text {vol}_{g_0}\nonumber \\ {}&\qquad +2\int _{\partial \Omega _2}k_{g_0}(-\mu _2+\theta _2)d\mathscr {H}^1_{g_0} +3\int _{\partial \Omega _2}\partial _{\nu }(-\mu _2+\theta _2)d\mathscr {H}^1_{g_0}. \end{aligned}$$

Notice that provided that $\Gamma$ is given with the same orientation of $\partial \Omega _1$, we have

3.14 $$\begin{aligned}&2\int _{\partial \Omega _1}k_{g_0}(-\mu _1+\theta _1)d\mathscr {H}^1_{g_0}+2\int _{\partial \Omega _2}k_{g_0}(-\mu _2+\theta _2)d\mathscr {H}^1_{g_0}\nonumber \\&\quad =2\int _{\Gamma }k_{g_0}(-\mu _1+\theta _1+\mu _2-\theta _2)\mathscr {H}^1_{g_0}. \end{aligned}$$

Since $K_{g_0}=1=-\Delta _{g_0}\mu _1$ on $\Omega _1$, we have

3.15 $$\begin{aligned} \int _{\Omega _1}K_{g_0}(-\mu _1)d\textrm{vol}_{g_0}&=\int _{\Omega _1}\mu _1\,\Delta _{g_0}\mu _1d\textrm{vol}_{g_0}\nonumber \\&=-\int _{\Omega _1}|d\mu _1|_{g_0}^2d\textrm{vol}_{g_0}+\int _{\Gamma }\mu _1\partial _{\nu }\mu _1d\mathscr {H}^1_{g_0}\nonumber \\&=-\int _{\Omega _1}|d\mu _1|_{g_0}^2d\textrm{vol}_{g_0}+\int _{\Gamma }\left( k_{g_0}-\partial _{\nu }G_{\Omega _1}\right) \mu _1d\mathscr {H}^1_{g_0} \end{aligned}$$

by (3.4). Therefore, we deduce that

3.16 $$\begin{aligned}&2\int _{\Omega _1}K_{g_0}(-\mu _1+\theta _1)d\textrm{vol}_{g_0} +2\int _{\partial \Omega _1}k_{g_0}(-\mu _1+\theta _1)d\mathscr {H}^1_{g_0}\nonumber \\&\quad =-2\int _{\Omega _1}|d\mu _1|_{g_0}^2d\textrm{vol}_{g_0}-2\int _{\Gamma }\partial _{\nu }G_{\Omega _1}\mu _1d\mathscr {H}^1_{g_0}+2\int _{\Omega _1}\theta _1 d\textrm{vol}_{g_0}\nonumber \\&\qquad +2\int _{\Gamma }k_{g_0}\theta _1d\mathscr {H}^1_{g_0}. \end{aligned}$$

Now, we have

3.17 $$\begin{aligned}&\int _{\Omega _1}|d(-\mu _1+\theta _1)|_{g_0}^2d\textrm{vol}_{g_0}=\int _{\Omega _1}|d(-\mu _1+\psi \circ f_1^{-1})|^2_{g_0}d\textrm{vol}_{g_0}\nonumber \\&\quad =\int _{\Omega _1}|d\mu _1|_{g_0}^2d\textrm{vol}_{g_0}-2\int _{\Omega _1}\langle d\mu _1,d(\psi \circ f_1^{-1})\rangle _{g_0}d\textrm{vol}_{g_0}\nonumber \\&\qquad +\int _{\Omega _1}|d(\psi \circ f_1^{-1})|_{g_0}^2d\textrm{vol}_{g_0}. \end{aligned}$$

Since $-\Delta _{g_0}\mu _1=1$, we deduce that

3.18 $$\begin{aligned} -2\int _{\Omega _1}\langle d\mu _1,d(\psi \circ f^{-1})\rangle _{g_0}d\textrm{vol}_{g_0}&=-2\int _{\Omega _1}\langle d\mu _1,d\theta _1\rangle _{g_0}d\textrm{vol}_{g_0}\nonumber \\&=2\int _{\Omega _1}\theta _1\Delta _{g_0}\mu _1d\textrm{vol}_{g_0}-2\int _{\Gamma }\theta _1\partial _{\nu }\mu _1d\mathscr {H}^1_{g_0}\nonumber \\&=-2\int _{\Omega _1}\theta _1d\textrm{vol}_{g_0}-2\int _{\Gamma }k_{g_0}\theta _1d\mathscr {H}^1_{g_0}\nonumber \\&\quad +2\int _{\Gamma }\partial _{\nu }G_{\Omega _1}\theta _1d\mathscr {H}^1_{g_0}. \end{aligned}$$

Now, by conformal invariance of the Dirichlet energy, we have

$$\begin{aligned} \int _{\Omega _1}|d(\psi \circ f^{-1})|^2_{g_0}d\text {vol}_{g_0}=\int _{\mathbb {D}}|\nabla \psi |^2|dz|^2. \end{aligned}$$

Since $\psi (z)=\log (2)-\log (1+|z|^2)$ and $\psi$ is real, we have

3.19 $$\begin{aligned} \int _{\mathbb {D}}|\nabla \psi |^2|dz|^2&=4\int _{\mathbb {D}}|\partial _{z}\psi |^2|dz|^2=4\int _{\mathbb {D}}\frac{|z|^2|dz|^2}{(1+|z|^2)^2}=4\pi \log (2)-2\pi . \end{aligned}$$

Therefore, we get, from (3.16), (3.17), (3.18) and (3.19),3.20Now, since $\theta _1=\psi \circ f^{-1}$, and $\psi (z)=0$ for all $z\in S^1$, we have $\theta _1=0$ on $\Gamma$. Therefore, we have

3.21 $$\begin{aligned} 2\int _{\Gamma }\partial _{\nu }G_{\Omega _1}(-\mu _1+\theta _1)d\mathscr {H}^1_{g_0}&=-2\int _{\Gamma }\partial _{\nu }G_{\Omega _1}\mu _1d\mathscr {H}^1_{g_0}. \end{aligned}$$

Now, since $-\Delta _{g_0}\mu _1=1$ and $K_{g_0}=1$, we have

3.22 $$\begin{aligned} \int _{\Omega _1}G_{\Omega _1}K_{g_0}d\textrm{vol}_{g_0}&=-\int _{\Omega _1}G_{\Omega _1}\Delta _{g_0}\mu _1d\textrm{vol}_{g_0}\nonumber \\&=-\int _{\Omega _1}\mu _1\Delta _{g_0}G_{\Omega _1}d\textrm{vol}_{g_0}-\int _{\Omega _1}\left( G_{\Omega _1}\partial _{\nu }\mu _1-\mu _1\partial _{\nu }G_{\Omega _1}\right) d\mathscr {H}^1_{g_0}\nonumber \\&=-2\pi \mu _1(p_1)+\int _{\partial \Omega _1}\mu _1\partial _{\nu }G_{\Omega _1}d\mathscr {H}^1_{g_0}, \end{aligned}$$

where we used the Dirichlet condition $G_{\Omega _1}=0$ on $\partial \Omega _1=\Gamma$. Therefore, (3.21) and (3.22) imply that

3.23 $$\begin{aligned} 2\int _{\Gamma }\partial _{\nu }G_{\Omega _1}(-\mu _1+\theta _1)d\mathscr {H}^1_{g_0}=-2\int _{\Omega _1}G_{\Omega _1}K_{g_0}d\textrm{vol}_{g_0}-4\pi \mu _1(p_1). \end{aligned}$$

Gathering (3.20) and (3.23) yields

3.24 $$\begin{aligned}&\int _{\Omega _1}|d(-\mu _1+\theta _1)|_{g_0}^2d\textrm{vol}_{g_0}+2\int _{\Omega _1}K_{g_0}(-\mu _1+\theta _1)d\textrm{vol}_{g_0}\nonumber \\&\qquad +2\int _{\partial \Omega _1}k_{g_0}(-\mu _1+\theta _1)d\mathscr {H}^1_{g_0}\nonumber \\&\quad =-\int _{\Omega _1}|d\mu _1|_{g_0}^2d\textrm{vol}_{g_0}-2\int _{\Omega _1}G_{\Omega _1}K_{g_0}d\textrm{vol}_{g_0}-4\pi \mu _1(p_1)+4\pi \log (2)-2\pi . \end{aligned}$$

We also have

3.25 $$\begin{aligned} \int _{\partial \Omega _1}\partial _{\nu }\left( -\mu _1+\theta _1\right) d\mathscr {H}^1_{g_0}+\int _{\partial \Omega _2}\partial _{\nu }\left( -\mu _2+\theta _2\right) d\mathscr {H}^1_{g_0}=0. \end{aligned}$$

Indeed, we have by the boundary conditions (3.10)

3.26 $$\begin{aligned} \int _{\partial \Omega _1}\partial _{\nu }\mu _1d\mathscr {H}^1_{g_0}&=\int _{\Gamma }k_{g_0}d\mathscr {H}^1_{g_0}-\int _{\partial \Omega }\partial _{\nu }G_{\Omega _j}d\mathscr {H}^1_{g_0}\nonumber \\ {}&=\int _{\Gamma }k_{g_0}d\mathscr {H}^1_{g_0}-\int _{\Omega _1}\Delta _{g_0}G_{\Omega _1}d\text {vol}_{g_0}\nonumber \\ {}&=\int _{\Gamma }k_{g_0}d\text {vol}_{g_0}-2\pi ;\nonumber \\ \int _{\partial \Omega _2}\partial _{\nu }\mu _2d\mathscr {H}^1_{g_0}&=-\int _{\Gamma }k_{g_0}d\text {vol}_{g_0}-2\pi . \end{aligned}$$

We also have by the conformal invariance of the Dirichlet energy

3.27 $$\begin{aligned} \int _{\partial \Omega _1}\partial _{\nu }\theta _1d\mathscr {H}^1_{g_0}=\int _{\Omega _1}\Delta _{g_0}\theta _1d\textrm{vol}_{g_0}=\int _{\mathbb {D}}\Delta \psi |dz|^2=\int _{S^1}\partial _{\nu }\psi \,d\mathscr {H}^1=-2\pi . \end{aligned}$$

Therefore, we finally, get by (3.26) and (3.27),

$$\begin{aligned}&\int _{\partial \Omega _1}\partial _{\nu }\left( -\mu _1+\theta _1\right) d\mathscr {H}^1_{g_0}+\int _{\partial \Omega _2}\partial _{\nu }\left( -\mu _2+\theta _2\right) d\mathscr {H}^1_{g_0}\\&\quad =-\left( \int _{\Gamma }k_{g_0}d\textrm{vol}_{g_0}-2\pi \right) -2\pi -\left( -\int _{\Gamma }k_{g_0}d\textrm{vol}_{g_0}-2\pi \right) -2\pi =0, \end{aligned}$$

which proves (3.25).

Finally, we deduce, by (3.13), (3.24) and (3.25), that

3.28 $$\begin{aligned}&-\pi \,I^L(\Gamma )=-\int _{\Omega _1}|d\mu _1|_{g_0}^2d\textrm{vol}_{g_0}-2\int _{\Omega _1}G_{\Omega _1}K_{g_0}d\textrm{vol}_{g_0}\nonumber \\&\qquad -4\pi \mu _1(p_1)+4\pi \log (2)-2\pi \nonumber \\&\qquad -\int _{\Omega _2}|d\mu _2|_{g_0}^2d\textrm{vol}_{g_0}-2\int _{\Omega _1}G_{\Omega _2}K_{g_0}d\textrm{vol}_{g_0}-4\pi \mu _2(p_2)+4\pi \log (2)-2\pi \nonumber \\&\quad =-\int _{\Omega _1}|d\mu _1|_{g_0}^2d\textrm{vol}_{g_0}-\int _{\Omega _2}|d\mu _2|_{g_0}^2d\textrm{vol}_{g_0}-2\int _{\Omega _1}G_{\Omega _1}K_{g_0}d\textrm{vol}_{g_0}\nonumber \\&\qquad -2\int _{\Omega _1}G_{\Omega _2}K_{g_0}d\textrm{vol}_{g_0}\nonumber \\&\qquad -4\pi \mu _1(p_1)-4\pi \mu _2(p_2)+8\pi \log (2)-4\pi . \end{aligned}$$

Recalling the identity (3.7), we finally deduce that

3.29 $$\begin{aligned} \pi \,I^L(\Gamma )&=\int _{\Omega _1}|d\mu _1|_{g_0}^2d\textrm{vol}_{g_0}+\int _{\Omega _2}|d\mu _2|_{g_0}^2d\textrm{vol}_{g_0}+2\int _{\Omega _1}G_{\Omega _1}K_{g_0}d\textrm{vol}_{g_0}\nonumber \\&\quad +2\int _{\Omega _1}G_{\Omega _2}K_{g_0}d\textrm{vol}_{g_0}+4\pi \nonumber \\&\quad +4\pi \log |\nabla f_1(0)|+4\pi \log |\nabla f_2(0)|-12\pi \log (2). \end{aligned}$$

Now we introduce the functional

3.30 $$\begin{aligned} \mathscr {E}(\Gamma )&=\int _{\Omega _1}|d\mu _1|^2_{g_0}d\textrm{vol}_{g_0}+\int _{\Omega _2}|d\mu _2|^2_{g_0}d\textrm{vol}_{g_0}+2\int _{\Omega _1}G_{\Omega _1}K_{g_0}d\textrm{vol}_{g_0}\nonumber \\&\quad +2\int _{\Omega _2}G_{\Omega _2}K_{g_0}d\textrm{vol}_{g_0}+4\pi . \end{aligned}$$

We deduce that

3.31 $$\begin{aligned} I^L(\Gamma )=\frac{1}{\pi }\mathscr {E}(\Gamma )+4\log |\nabla f_1(0)|+4\log |\nabla f_2(0)|-12\log (2). \end{aligned}$$

This concludes the proof of the theorem. $\quad \square$

### Remark 3.8

We check that equality (3.31) holds for the equator $S^1$. Using the definition (1.3) with the conformal maps *f*, *g* being the identity maps, we see that $I^L(S^1)$ vanishes.

Let us first check that

$$\begin{aligned} \mathscr {E}(S^1)=0 \end{aligned}$$

with the marked points $p_1 = S = (0,0,-1)$ and $p_2 = N = (0,0,1)$. This identity justifies the term $4\pi$ in the definition of $\mathscr {E}$ as we remarked earlier.

For this, since $K_{g_0}=1$ on $\Omega _1 = S_-^2$, after making a stereographic projection $\pi : S^2 \setminus \{N \} \rightarrow \mathbb C$ sending *S* to 0, we find that

3.32 $$\begin{aligned} \int _{S^2_{-}} G_{S^2_{-}}K_{g_0}d\textrm{vol}_{g_0}&=\int _{\mathbb {D}}G_{\mathbb {D}}(z)\frac{4|dz|^2}{(1+|z|^2)^2}=\int _{\mathbb {D}}\frac{4\log |z|}{(1+|z|^2)^2}|dz|^2\nonumber \\&=8\pi \int _{0}^{1}\frac{r\log r}{(1+r^2)^2}dr\nonumber \\&=8\pi \left[ -\frac{1}{2(1+r^2)}\log (r)+\frac{1}{2}\log (r)-\frac{1}{4}\log (1+r^2)\right] _0^1\nonumber \\&=-2\pi \log (2). \end{aligned}$$

We take $f_1 = \pi ^{-1}|_{\mathbb D}$ which is consistent with $f_1(0) = p_1 = S$. By (3.4) we have $\partial _\nu \mu = -1$ on $S^1$ and $- \Delta _{g_0}\mu =1$ in $S^2_-$ which translates to

$$\begin{aligned} -\Delta \mu (z) =\frac{4}{(1+|z|^2)^2} \qquad \text {in}\;\,\mathbb {D}. \end{aligned}$$

We deduce by a direct verification that $\mu (z)=-\log (1+|z|^2)$. (This is easy to guess since by (3.6), $\mu$ can be computed from the conformal factor of $f_1$.) Therefore, we have by the conformal invariance of the Dirichlet energy

3.33 $$\begin{aligned} \int _{S^2_{-}}\left| \omega -*dG_{S^2_{-}}\right| ^2_{g_0}d\textrm{vol}_{g_0}&=\int _{S^2_{-}}|d\mu |_{g_0}^2d\textrm{vol}_{g_0}\nonumber \\&=\int _{\mathbb {D}}|\nabla \mu (x)|^2dx=\int _{\mathbb {D}}\left| \frac{2x}{1+|x|^2}\right| ^2\hbox {d}x \nonumber \\&=8\pi \int _{0}^1\frac{r^3}{(1+r^2)^2}\hbox {d}r\nonumber \\&=8\pi \int _{0}^1\left( \frac{r}{1+r^2}-\frac{r}{(1+r^2)^2}\right) \hbox {d}r\nonumber \\&=8\pi \left[ \frac{1}{2}\log (1+r^2)+\frac{1}{2}\frac{1}{1+r^2}\right] _0^1\nonumber \\&=8\pi \left( \frac{1}{2}\log (2)-\frac{1}{4}\right) =4\pi \log (2)-2\pi . \end{aligned}$$

Finally, by (3.32) and (3.33), we have

$$\begin{aligned}&\int _{S^2_{-}}|\omega -*\, dG_{S^2_{-}}|_{g_0}^2d\textrm{vol}_{g_0}+2\int _{S^2_{-}}G_{S^2_{-}}\,K_{g_0}d\textrm{vol}_{g_0}+2\pi \\&\quad =\left( 4\pi \log (2)-2\pi \right) -4\pi \log (2)+2\pi =0. \end{aligned}$$

Applying the same computation to $\Omega _2 = S^2_+$ with $f_2 = - f_1 : \mathbb D \rightarrow S^2_+$ which is consistent with the choice $p_2 = N = f_2 (0)$, we obtain the claimed identity $\mathscr {E}(S^1)=0$.

Now we show that

3.34 $$\begin{aligned} 4\log |\nabla f_1(0)|+4\log |\nabla f_2(0)| -12\log (2) =0. \end{aligned}$$

Since the inverse stereographic projection is given by

$$\begin{aligned} f_1 (z) = \pi ^{-1}(z )=\left( \frac{2\,\textrm{Re}\,(z)}{1+|z|^2},\frac{2\,\textrm{Im}\,(z)}{1+|z|^2},\frac{-1+|z|^2}{1+|z|^2}\right) , \end{aligned}$$

we compute directly that

$$\begin{aligned} |\nabla f_1(0)| = |\nabla f_2(0)| =2\sqrt{2} \end{aligned}$$

which concludes the proof of (3.34) and shows the identity (3.31) for the circle $S^1$.

## Construction of Harmonic Moving Frames for Weil–Petersson Curves

In the previous section, we showed that in the case of smooth curves, the Loewner energy was equal to a renormalised Dirichlet energy of a specific harmonic moving frame. In this section, we will directly construct harmonic moving frames satisfying appropriate boundary conditions for arbitrary Weil–Petersson quasicircles. In the next section, we will show that Theorem 3.6 holds for non-smooth curves.

Before stating the main theorem of this section, we recall an easy lemma on harmonic vector fields.

### Lemma 4.1

Let $\Sigma \subset \mathbb {R}^3$ be a smooth surface, $\vec {n}:\Sigma \rightarrow S^2$ its unit normal, and $g=\iota ^{*}g_{\mathbb {R}^3}$ be the induced metric. Assume that $\vec {u}:\Sigma \rightarrow S^2$ is a smooth critical point of the Dirichlet energy amongst $S^2$-valued maps such that $\langle \vec u,\vec {n}\hspace{0.1em}\rangle =0$. Then $\vec {u}$ satisfies the following Euler-Lagrange equation:

4.1 $$\begin{aligned} -\Delta _g\vec {u}=|d\vec {u}\hspace{0.1em}|_g^2\vec {u}+\left( 2\langle d\vec {u},d\vec {n}\hspace{0.1em}\rangle _g+\langle \vec {u},\Delta _g\vec {n}\hspace{0.1em}\rangle \right) \vec {n}, \end{aligned}$$

and we say that $\vec {u}$ is a harmonic vector field. If $U\Sigma$ is the unitary tangent bundle of $\Sigma$, and $(\vec {u},\vec {v}):\Sigma \rightarrow U\Sigma \rightarrow U\Sigma \subset S^2\times S^2$ are harmonic vector fields such that $\langle \vec {u},\vec {v}\hspace{0.1em}\rangle =0$, we see that $(\vec {u},\vec {v})$ is a harmonic moving frame.

### Proof

We proceed as in Lemme (1.4.10) of [29] , taking variations $\vec {X}$ that also satisfy $\langle \vec {X},\vec {n}\hspace{0.1em}\rangle =0$. $\quad \square$

### Remark 4.2

On can also recover the equation as in [29, Exemple (1.2.7)]. Indeed, if $\vec {u}:\Sigma \rightarrow S^2$ were a critical point of the Dirichlet energy, we would only get the standard harmonic map equation (2.12). However, due to the constraint that $\vec {u}$ is a vector field, there is an additional normal component. Using that $T_{\vec {u}(x)}^{\perp }S^2=\mathbb {R}\,\vec {u}(x)$, we deduce that there exists $\lambda _1,\lambda _2\in \mathbb {R}$ such that

4.2 $$\begin{aligned} \Delta _g\vec {u}=\lambda _1\,\vec {u}+\lambda _2\,\vec {n}. \end{aligned}$$

Since $|\vec {u}\hspace{0.1em}|^2=1$, taking the Laplacian of this equation, we deduce that $\langle \Delta _g\vec {u},\vec {u}\hspace{0.1em}\rangle =-|d\vec {u}\hspace{0.1em}|_g^2$. Likewise, as $\vec {u}$ is a vector field, we have

$$\begin{aligned} 0=\Delta _g\left( \langle \vec {u},\vec {n}\hspace{0.1em}\rangle \right) =\langle \Delta _g\vec {u},\vec {n}\hspace{0.1em}\rangle +2\langle d\vec {u},d\vec {n}\hspace{0.1em}\rangle _g+\langle \vec {u},\Delta _g\vec {n}\hspace{0.1em}\rangle . \end{aligned}$$

Therefore, using once more that $\langle \vec {u},\vec {n}\hspace{0.1em}\rangle =0$, taking the product of (4.2) shows that

$$\begin{aligned} \left\{ \begin{aligned} \lambda _1&=\langle \Delta _g\vec {u},\vec {u}\rangle =-|d\vec {u}\hspace{0.1em}|_g^2\\ \lambda _2&=\langle \Delta _g\vec {u},\vec {n}\hspace{0.1em}\rangle =-\left( 2\langle d\vec {u},d\vec {n}\hspace{0.1em}\rangle _g+\langle \vec {u},\Delta _g\vec {n}\hspace{0.1em}\rangle \right) \end{aligned}\right. \end{aligned}$$

and finally, (4.2) is equivalent to

4.3 $$\begin{aligned} -\Delta _g\vec {u}=|d\vec {u}\hspace{0.1em}|_g^2\vec {u}+\left( \langle d\vec {u},d\vec {n}\hspace{0.1em}\rangle _g+\langle \vec {u},\Delta _g\vec {n}\hspace{0.1em}\rangle \right) \vec {n}. \end{aligned}$$

The following result is the same as Theorem 3.6, but for a general Weil–Petersson quasicircle:

### Theorem 4.3

Let $\Gamma \subset S^2$ a Weil–Petersson quasicircle and let $\Omega _1,\Omega _2$ be the two open connected components of $S^2\setminus \Gamma$. For $j=1,2$ and for all $p_j\in \Omega _j$, there exists a harmonic moving frame $(\vec {u}_j,\vec {v}_j):\Omega _j\setminus \left\{ p_j\right\} \rightarrow U\Omega _j\times U\Omega _j$ such that the Cartan form $\omega _j=\langle \vec {u}_j,d\vec {v}_j\rangle$ admits the decomposition

4.4 $$\begin{aligned} \omega _j=*\,d\left( G_{\Omega _j}+\mu _j\right) , \end{aligned}$$

where $G_{\Omega _j}=G_{\Omega _j,p_j}:\Omega _j\setminus \left\{ p_j\right\} \rightarrow \mathbb {R}$ is the Green’s function of the Laplacian $\Delta _{g_0}$ on $\Omega _j$ with Dirichlet boundary condition and singularity $p_j\in \Omega _j$, and $\mu _j\in C^{\infty }(\Omega _j)$ satisfies

4.5 $$\begin{aligned} \left\{ \begin{aligned} -\Delta _{g_0}\mu _j&=1\qquad{} & {} \text {in}\;\,\Omega _j\\ \partial _{\nu }\mu _j&=k_{g_0}-\partial _{\nu }G_{\Omega _j}\qquad{} & {} \text {on}\;\,\partial \Omega _j, \end{aligned} \right. \end{aligned}$$

where $k_{g_0}$ is the geodesic curvature on $\Gamma =\partial \Omega _j$.

### Remark 4.4

The Neumann condition for $\mu _j$ ($1\le j\le 2$) is understood in the sense of distributions, since the geodesic curvature is only in $H^{-1/2}(\Gamma )$ in general (see the appendix for more details).

### Proof

Rather than using the moving frame that comes from a Ginzburg–Landau type minimisation as in [34]—that would have had to be carried in the geometric setting of domains of $S^2$—we directly use the uniformisation theorem and the geometric formula of [59] (that does not require any regularity on the curve $\Gamma$) to construct the relevant moving frame. We now construct the moving frame on $\Omega _1$. The construction for $\Omega _2$ is similar.

**Step 1.** Definition of $(\vec {u}_{1},\vec {v}_{1})$ and $\mu _{1}$.

Let $\pi :S^2\setminus \left\{ N\right\} \rightarrow \mathbb {C}$ be the standard stereographic projection and assume without loss of generality that $N\in \Omega _2$. Let $\Omega =\pi (\Omega _1)\subset \mathbb {C}$ be the image domain and $\gamma =\pi (\Gamma )\subset \mathbb {C}$ be the image curve. Thanks to the uniformisation theorem, there exists a univalent holomorphic map $f:\mathbb {D}\rightarrow \Omega$ such that $f(0) = \pi (p_1)$.

Now, let $f_1=\pi ^{-1}\circ f:\mathbb {D}\rightarrow \Omega _1$. Notice that $f_1 (0) = p_1$. Explicitly, we have

$$\begin{aligned} f_1(z)=\pi ^{-1}(f(z))=\left( \frac{2\,\textrm{Re}\,(f(z))}{1+|f(z)|^2},\frac{2\,\textrm{Im}\,(f(z))}{1+|f(z)|^2},\frac{-1+|f(z)|^2}{1+|f(z)|^2}\right) . \end{aligned}$$

A direct computation show that

$$\begin{aligned} \partial _{z}f_1=f'\left( \frac{(1-\overline{f}^2)}{(1+|f|^2)^2},\frac{-i(1+\overline{f}^2)}{(1+|f|^2)^2},\frac{2\overline{f}}{(1+|f|^2)^2}\right) . \end{aligned}$$

Now, by analogy with the construction in Section 3 (see also [34], Proposition 5.1), define $\mu _1:\Omega _1\rightarrow \mathbb {R}$ and $\vec {u}_1:\Omega _1\rightarrow U S^2$ and $\vec {v}_1:\Omega _1\rightarrow U S^2$ by

$$\begin{aligned} \left\{ \begin{aligned} \partial _{r}f_1&=e^{\mu _1\circ f_1}\vec {v}_1\circ f_1\\ \frac{1}{r}\partial _{\theta }f_1&=e^{\mu _1\circ f_1}\vec {u}_1\circ f_1. \end{aligned}\right. \end{aligned}$$

Then, we have from direct computations

$$\begin{aligned} e^{2\mu _1\circ f_1}=|\partial _{r}f_1|^2=\frac{1}{r^2}|\partial _{\theta }f_1|^2=2|\partial _{z}f_1|^2=\frac{4|f'(z)|^2}{(1+|f(z)|^2)^2}. \end{aligned}$$

Therefore, we deduce if $\mu =\mu _1\circ f_1$ that

4.6 $$\begin{aligned} \mu (z)=\log |f'(z)|-\log \left( 1+|f(z)|^2\right) +\log (2). \end{aligned}$$

Since $\partial _{z}=\dfrac{1}{2}(\partial _{x}-i\,\partial _{y})$, we have

$$\begin{aligned} \left\{ \begin{aligned} \partial _{r}f_1&=\cos (\theta )\partial _{x}f_1+\sin (\theta )\partial _{y}f_1=\textrm{Re}\,\left( \frac{z}{|z|}\right) \textrm{Re}\,(\partial _{z}f_1)-\textrm{Im}\,\left( \frac{z}{|z|}\right) \textrm{Im}\,\left( \partial _{z}f_1\right) \\ \frac{1}{r}\partial _{\theta }f_1&=-\sin (\theta )\partial _{x}f_1+\cos (\theta )\partial _{y}f_1=-\textrm{Im}\,\left( \frac{z}{|z|}\right) \textrm{Re}\,(\partial _{z}f_1)-\textrm{Re}\,\left( \frac{z}{|z|}\right) \textrm{Im}\,\left( \partial _{z}f_1\right) . \end{aligned}\right. \end{aligned}$$

By the elementary identities for all $a,b\in \mathbb {C}$

$$\begin{aligned} \left\{ \begin{aligned} \textrm{Re}\,(a)\textrm{Re}\,(b)+\textrm{Im}\,(a)\textrm{Im}\,(b)&=\textrm{Re}\,(ab)\\ \textrm{Re}\,(a)\textrm{Im}\,(b)+\textrm{Im}\,(a)\textrm{Re}\,(b)&=-\textrm{Im}\,(ab), \end{aligned}\right. \end{aligned}$$

we deduce that

$$\begin{aligned} \left\{ \begin{aligned} \partial _{r}f_1&=\textrm{Re}\,\left( \frac{z}{|z|}\partial _{z}f_1\right) \\&=\textrm{Re}\,\left( \frac{\overline{z}}{|z|}\overline{f'(z)}\left( \frac{(1-f(z)^2)}{(1+|f(z)|^2)^2},\frac{i(1+f(z)^2)}{(1+|f(z)|^2)^2},\frac{2f(z)}{(1+|f(z)|^2)^2}\right) \right) \\ \frac{1}{r}\partial _{\theta }f_1&=-\textrm{Im}\,\left( \frac{z}{|z|}\partial _{z}f_1\right) \\&=\textrm{Im}\,\left( \frac{\overline{z}}{|z|}\overline{f'(z)}\left( \frac{(1-f(z)^2)}{(1+|f(z)|^2)^2},\frac{i(1+f(z)^2)}{(1+|f(z)|^2)^2},\frac{2f(z)}{(1+|f(z)|^2)^2}\right) \right) . \end{aligned}\right. \end{aligned}$$

More generally, if $\varphi :\mathbb {C}\rightarrow \mathbb {C}$ is a smooth complex function, we have

4.7 $$\begin{aligned} \partial _{\theta }\varphi =-\textrm{Im}\,\left( z\right) \left( \partial _{z}+\partial _{\overline{z}}\right) \varphi +\textrm{Re}\,\left( {z}\right) i\left( \partial _{z}-\partial _{\overline{z}}\right) \varphi =i\left( z\,\partial _{z}\varphi -\overline{z}\,\partial _{\overline{z}}\varphi \right) . \end{aligned}$$

Since

$$\begin{aligned} |\partial _{z}f_1|=\frac{1}{r}|\partial _{\theta }f_1|=\frac{2|f'(z)|}{1+|f(z)|^2}, \end{aligned}$$

we deduce that

4.8 $$\begin{aligned} \left\{ \begin{aligned} \vec {v}_1\circ f_1&=\textrm{Re}\,\left( \overline{\frac{zf'(z)}{|z f'(z)|}}\left( \frac{(1-f(z)^2)}{(1+|f(z)|^2)},\frac{i(1+f(z)^2)}{(1+|f(z)|^2)},\frac{2f(z)}{(1+|f(z)|^2)}\right) \right) \\ \vec {u}_1\circ f_1&=\textrm{Im}\,\left( \overline{\frac{zf'(z)}{|z f'(z)|}}\left( \frac{(1-f(z)^2)}{(1+|f(z)|^2)},\frac{i(1+f(z)^2)}{(1+|f(z)|^2)},\frac{2f(z)}{(1+|f(z)|^2)}\right) \right) . \end{aligned}\right. \end{aligned}$$

Notice that

$$\begin{aligned} F(z)=\left( (1-f(z)^2),i(1+f(z)^2),2f(z)\right) \end{aligned}$$

is a holomorphic null vector, *i.e.* $\langle F(z),F(z)\rangle =0$ (notice that here, $\langle \,\cdot \,,\,\cdot \,\rangle$ is the *real* scalar product on $\mathbb {R}^3$, extended by linearity on $\mathbb {C}^3$), so we see directly since $|\vec {u}_1|=|\vec {v}_1|=1$ that

$$\begin{aligned} \langle \vec {u}_1,\vec {v}_1\rangle =0. \end{aligned}$$

**Step 2.** Verification of the system (4.5).

**Part 1.** Equation on $\Omega _1$ for $\mu _1$.

Since $\mu =\mu _1\circ f_1$, the equation $-\Delta _{g_0}\mu _1=1$ is equivalent to

4.9 $$\begin{aligned} -\Delta \mu =e^{2\mu } \end{aligned}$$

Thanks to the explicit expression in (4.6), and by harmonicity of $\log |f'|$, we have

$$\begin{aligned} -\Delta \mu&=4\,\partial _{\overline{z}}\left( \frac{f'(z)\overline{f'(z)}}{1+|f(z)|^2}\right) =4\left( \frac{|f'(z)|^2}{1+|f(z)|^2}-\frac{|f'(z)|^2|f(z)|^2}{(1+|f(z)|^2)^2}\right) \\&=\frac{4|f'(z)|^2}{(1+|f(z)|^2)^2}=e^{2\mu }. \end{aligned}$$

Recalling that

$$\begin{aligned} g_{\widehat{\mathbb {C}}} =\frac{4|dz|^2}{(1+|z|^2)^2}=(\pi ^{-1})^{*}g_{0}, \end{aligned}$$

we deduce that

$$\begin{aligned} \frac{4|f'(z)|^2|dz|^2}{(1+|f(z)|^2)^2}=f^{*} g_{\widehat{\mathbb {C}}} = f^{*}((\pi ^{-1})^{*} g_{0})=(\pi ^{-1}\circ f)^{*} g_{0} = f_1^{*} g_{0}. \end{aligned}$$

Therefore, (4.9) can be rewritten as

$$\begin{aligned} -\Delta _{f_1^{*}g_{0}}(\mu _1\circ f_1)=1 \end{aligned}$$

or by conformal invariance of the Dirichlet energy

4.10 $$\begin{aligned} -\Delta _{g_{0}}\mu _1=1. \end{aligned}$$

**Part 2.** Boundary conditions.

If ${h}:\mathbb {C}\rightarrow \mathbb {R}$ is a smooth function, we have

4.11 $$\begin{aligned} \partial _{\nu }{h}&=\frac{x}{\sqrt{x^2+y^2}}\partial _{x}{h}+\frac{y}{\sqrt{x^2+y^2}}\partial _{y}{h}=\frac{\textrm{Re}\,(z)}{|z|}\left( \partial _{z}+\partial _{\overline{z}}\right) {h} +\frac{\textrm{Im}\,(z)}{|z|}i\left( \partial _{z}-\partial _{\overline{z}}\right) {h}\nonumber \\&=2\,\textrm{Re}\,\left( \frac{z}{|z|}\partial _{z}{h}\right) . \end{aligned}$$

This implies since $\mu (z)=\log |f'(z)|-\log (1+|f(z)|^2)+\log (2)$ by (4.6) that

$$\begin{aligned} \partial _{z}\mu (z)=\frac{1}{2}\frac{f''(z)}{f'(z)}-\frac{f'(z)}{f(z)}\frac{|f(z)|^2}{1+|f(z)|^2}, \end{aligned}$$

and

$$\begin{aligned} \partial _{\nu }\mu =2\,\textrm{Re}\,\left( \frac{z}{|z|}\partial _{z}\mu \right) =\textrm{Re}\,\left( z\frac{f''(z)}{f'(z)}-2z\frac{f'(z)}{f(z)}\frac{|f(z)|^2}{1+|f(z)|^2}\right) \end{aligned}$$

on $\partial \mathbb D$ in the distributional sense. We will comment on it in Remark 5.6. Recall that the geodesic curvature on $\partial \Omega _1$ is given (see [17]) by

4.12 $$\begin{aligned} k_{g_0}=\langle \vec {u}_1,\partial _{\theta }\vec {v}_1\rangle . \end{aligned}$$

From (4.8) it is natural to define

$$\begin{aligned} \varphi (z)=\overline{\frac{zf'(z)}{|z f'(z)|}}\left( \frac{(1-f(z)^2)}{(1+|f(z)|^2)},\frac{i(1+f(z)^2)}{(1+|f(z)|^2)},\frac{2f(z)}{(1+|f(z)|^2)}\right) =\chi (z)\psi (z), \end{aligned}$$

where

4.13 $$\begin{aligned} \left\{ \begin{aligned} \chi (z)&=\frac{\overline{zf'(z)}}{|zf'(z)|}=\exp \left( \frac{1}{2}\log \left( \overline{zf'(z)}\right) -\frac{1}{2}\log \left( zf'(z)\right) \right) \\ \psi (z)&=\left( \frac{1-f(z)^2}{1+|f(z)|^2},\frac{i(1+f(z)^2)}{1+|f(z)|^2},\frac{2f(z)}{1+|f(z)|^2}\right) , \end{aligned}\right. \end{aligned}$$

so that $\vec {v}_1 \circ f_1 = \textrm{Re}\,(\varphi )$ and $\vec {u}_1 \circ f_1 = \textrm{Im}\,(\varphi )$. Then, we compute

4.14 $$\begin{aligned} \left\{ \begin{aligned} \partial _{z}\chi&=-\frac{1}{2}\left( \frac{f''(z)}{f'(z)}+\frac{1}{z}\right) \chi \\ \partial _{\overline{z}}\chi&=\frac{1}{2}\overline{\left( \frac{f''(z)}{f'(z)}+\frac{1}{z}\right) }\chi . \end{aligned}\right. \end{aligned}$$

We also get

4.15 $$\begin{aligned} \left\{ \begin{aligned} \partial _{z}\psi&=-\frac{f'(z)\overline{f(z)}}{1+|f(z)|^2}\psi +\frac{2f'(z)}{1+|f(z)|^2}\left( -f(z),i\,f(z),1\right) \\ \partial _{\overline{z}}\psi&=-\frac{\overline{f'(z)}f(z)}{1+|f(z)|^2}\psi . \end{aligned}\right. \end{aligned}$$

Since $\langle \psi ,\psi \rangle =0$, we have $\langle \partial _{z}\psi ,\psi \rangle =\langle \partial _{\overline{z}}\psi ,\psi \rangle =0$. In particular, we have

4.16 $$\begin{aligned}&\langle (-f(z),i\,f(z),1),\psi \rangle \nonumber \\ {}&=\frac{1}{1+|f(z)|^2}\left\langle (-f(z),i\,f(z),1),(1-f(z)^2,i(1+f(z)^2),2f(z))\right\rangle =0, \end{aligned}$$

while

$$\begin{aligned}&\left\langle (-f(z),i\,f(z),1),\left( 1-\overline{f(z)}^2,-i\left( 1+\overline{f(z)}^2\right) ,2\overline{f(z)}\right) \right\rangle \\&\quad =-f(z)+\overline{f(z)}|f(z)|^2+f(z)+\overline{f(z)}|f(z)|^2+2{\overline{f(z)}} =2\overline{f(z)}(1+|f(z)|^2), \end{aligned}$$

so that

4.17 $$\begin{aligned} \langle (-f(z),i\,f(z),1),{\overline{\psi }}\rangle =2\overline{f(z)}. \end{aligned}$$

Therefore, we deduce, by (4.15), (4.16), and (4.17), that

4.18 $$\begin{aligned} \left\{ \begin{aligned}&\langle \varphi ,\varphi \rangle =\langle \partial _{z}\varphi ,\varphi \rangle =\langle \partial _{\overline{z}}\varphi ,\varphi \rangle =\langle \psi ,\psi \rangle =\langle \partial _{z}\psi ,\psi \rangle =\langle \partial _{\overline{z}}\psi ,\psi \rangle =0\\&\langle \partial _{z}\psi ,\overline{\psi }\rangle = \frac{2f'(z)\overline{f(z)}}{1+|f(z)|^2}\\&\langle \partial _{\overline{z}}\psi ,\overline{\psi }\rangle =-\frac{2\overline{f'(z)}f(z)}{1+|f(z)|^2}. \end{aligned}\right. \end{aligned}$$

The identities (4.14) and (4.18) imply that

$$\begin{aligned} z\,\partial _{z}\varphi -\overline{z}\,\partial _{z}\varphi =-\left( \textrm{Re}\,\left( z\frac{f''(z)}{f'(z)}\right) +1\right) \varphi +\chi (z)\left( z\,\partial _{z}\psi -\overline{z}\,\partial _{\overline{z}}\psi \right) , \end{aligned}$$

and since $|\chi |^2=1$ and $|\psi |^2=2$, we have

$$\begin{aligned}&\langle z\,\partial _{z}\varphi -\overline{z}\,\partial _{\overline{z}}\varphi ,\varphi +\overline{\varphi }\rangle =-2\,\left( \textrm{Re}\,\left( z\frac{f''(z)}{f'(z)}\right) +1\right) \\&\qquad +\chi (z)\left( z\left( \chi (z)\langle \partial _{z}\psi ,\psi \rangle +\overline{\chi (z)}\langle \partial _{z}\psi ,\overline{\psi }\rangle \right) \right. \\&\qquad \left. -\overline{z}\left( \chi (z)\langle \partial _{\overline{z}}\varphi ,\varphi \rangle +\overline{\chi (z)}\langle \partial _{\overline{z}}\psi ,\overline{\psi }\rangle \right) \right) \\&\quad =-2\,\left( \textrm{Re}\,\left( z\frac{f''(z)}{f'(z)}\right) +1\right) +2z\frac{f'(z)}{f(z)}\frac{|f(z)|^2}{1+|f(z)|^2}+2\overline{z}\overline{\frac{f'(z)}{f(z)}}\frac{|f(z)|^2}{1+|f(z)|^2}\\&\quad =-2-2\,\textrm{Re}\,\left( z\frac{f''(z)}{f'(z)}-2z\frac{f'(z)}{f(z)}\frac{|f(z)|^2}{1+|f(z)|^2}\right) , \end{aligned}$$

so that

4.19 $$\begin{aligned} k_{g_0}&=\langle \vec {u},\partial _{\theta }\vec {v}\rangle =-\langle \partial _{\theta }\vec {u},\vec {v}\rangle =-\langle \partial _{\theta }\textrm{Im}\,(\varphi ),\textrm{Re}\,(\varphi )\rangle =-\textrm{Im}\,\left( \langle \partial _{\theta }\varphi ,\textrm{Re}\,(\varphi )\rangle \right) \nonumber \\&=-\frac{1}{2}\textrm{Im}\,\left( \langle i\left( z\partial _{z}\varphi -\overline{z}\partial _{\overline{z}}\right) ,\varphi +\overline{\varphi }\rangle \right) =-\frac{1}{2}\textrm{Re}\,\left( \langle z\partial _{z}\varphi -\overline{z}\partial _{\overline{z}}\varphi ,\varphi +\overline{\varphi }\rangle \right) \nonumber \\&=\textrm{Re}\,\left( z\frac{f''(z)}{f'(z)}-2z\frac{f'(z)}{f(z)}\frac{|f(z)|^2}{1+|f(z)|^2}\right) +1=\partial _{\nu }\mu +1=\partial _{\nu }\mu +\partial _{\nu }G_{\mathbb {D}}, \end{aligned}$$

which concludes the proof that $\mu _1$ solves the system (4.5) by the conformal invariance of the Green’s function (we denoted for simplicity $G_{\mathbb {D}}=G_{\mathbb {D},0}=\log |\,\cdot \,|$).

**Step 3.** Verification that $(\vec {u}_1,\vec {v}_1)$ is a harmonic moving frame.

Now, thanks to Lemma 4.1 and (4.1), the maps $\vec {u}_1$ and $\vec {v}_1$ are unit harmonic moving frames if and only if they satisfy in the distributional sense (refer to Theorem 4.6 for the proof of this equivalence) the system (writing $\vec {u}=\vec {u}_1\circ f_1$ and $\vec {v}=\vec {v}_1\circ f_1$ for simplicity)

4.20 $$\begin{aligned} \left\{ \begin{aligned} -\Delta \vec {u}&=|\nabla \vec {u}|^2\vec {u}+\left( 2\langle \nabla \vec {u},\nabla \vec {n}\hspace{0.1em}\rangle +\langle \vec {u},\Delta \vec {n}\hspace{0.1em}\rangle \right) \vec {n}\\ -\Delta \vec {v}&=|\nabla \vec {v}|^2\vec {v}+\left( 2\langle \nabla \vec {v},\nabla \vec {n}\hspace{0.1em}\rangle +\langle \vec {v},\Delta \vec {n}\hspace{0.1em}\rangle \right) \vec {n}, \end{aligned}\right. \end{aligned}$$

where $\vec {n}:\mathbb {D}\rightarrow S^2$ is the same map as $f_1$ but viewed as the Gauss map associated to the branched minimal immersion of the disk from $\mathbb {D}$ into $\mathbb {R}^3$ with Weierstrass data (*f*, *dz*). It is given by

$$\begin{aligned} \vec {n}(z)=\left( \frac{2\,\textrm{Re}\,(f(z))}{1+|f(z)|^2},\frac{2\,\textrm{Im}\,(f(z))}{1+|f(z)|^2},\frac{-1+|f(z)|^2}{1+|f(z)|^2}\right) . \end{aligned}$$

By a direct computation, we see that the Gauss map satisfies the following equations

$$\begin{aligned} |\nabla \vec {n}(z)|^2&=\frac{8|f'(z)|^2}{(1+|f(z)|^2)^2};\\ -\Delta \vec {n}&=|\nabla \vec {n}\hspace{0.1em}|^2\vec {n}. \end{aligned}$$

In particular, the previous equation (4.20) must reduce to

4.21 $$\begin{aligned} \left\{ \begin{aligned} -\Delta \vec {u}&=|\nabla \vec {u}\hspace{0.1em}|^2\vec {u}+2\langle \nabla \vec {u},\nabla \vec {n}\hspace{0.1em}\rangle \vec {n}\\ -\Delta \vec {v}&=|\nabla \vec {v}\hspace{0.1em}|^2\vec {v}+2\langle \nabla \vec {v},\nabla \vec {n}\hspace{0.1em}\rangle \vec {n}. \end{aligned}\right. \end{aligned}$$

However, since $\langle \vec {u},\Delta \vec {n}\hspace{0.1em}\rangle =|\nabla \vec {n}\hspace{0.1em}|^2\langle \vec {u},\vec {n}\hspace{0.1em}\rangle =0$, we deduce that $-\langle \Delta \vec {u},\vec {n}\hspace{0.1em}\rangle =2\langle \nabla \vec {u},\nabla \vec {n}\hspace{0.1em}\rangle$, and since $|\nabla \vec {u}\hspace{0.1em}|^2=1$, we also get (by taking the Laplacian of $|\vec {u}\hspace{0.1em}|^2=1$) that $-\langle \Delta \vec {u},\vec {u}\hspace{0.1em}\rangle =|\nabla \vec {u}\hspace{0.1em}|^2\vec {u}$. Therefore, we need only check that

4.22 $$\begin{aligned} \langle \Delta \vec {u},\vec {v}\,\rangle =\langle \Delta \vec {v},\vec {u}\,\rangle =0 \end{aligned}$$

to show that $\vec {u}$ and $\vec {v}$ satisfy the equations (4.21). Recall from (4.13) that $\vec {u}=\textrm{Re}\,(\varphi )$ and $\vec {v}=\textrm{Im}\,(\varphi )$, we deduce that

$$\begin{aligned} \Delta \vec {u}=\textrm{Re}\,(\Delta \varphi ),\qquad \Delta \vec {v}=\textrm{Im}\,(\Delta \varphi ) \end{aligned}$$

and we have

4.23 $$\begin{aligned} \left\{ \begin{aligned} \langle \textrm{Re}\,(\Delta \varphi ),\textrm{Im}\,(\varphi )\rangle&=\frac{1}{2}\textrm{Im}\,\left( \langle \Delta \varphi ,\varphi \rangle \right) -\frac{1}{2}\textrm{Im}\,(\langle \Delta \varphi ,\overline{\varphi }\rangle )\\ \langle \textrm{Im}\,(\Delta \varphi ),\textrm{Re}\,(\varphi )\rangle&=\frac{1}{2}\textrm{Im}\,\left( \langle \Delta \varphi ,\varphi \rangle \right) +\frac{1}{2}\textrm{Im}\,(\langle \Delta \varphi ,\overline{\varphi }\rangle ). \end{aligned}\right. \end{aligned}$$

Therefore, the equations (4.22) are equivalent to

4.24 $$\begin{aligned} \textrm{Im}\,(\langle \Delta \varphi ,\varphi \rangle )=\textrm{Im}\,(\langle \Delta \varphi ,\overline{\varphi }\rangle )=0. \end{aligned}$$

Using (4.15), (4.16) and (4.18), we get

4.25 $$\begin{aligned}&\langle \Delta \varphi ,\varphi \rangle =-4\langle \partial _{z}\varphi ,\partial _{\overline{z}}\varphi \rangle \nonumber \\ {}&\quad =-4\left\langle -\frac{1}{2}\left( \frac{f''(z)}{f'(z)}+\frac{1}{z}\right) \varphi -\frac{f'(z)\overline{f(z)}}{1+|f(z)|^2}\left( -f(z),i\,f(z),1\right) ,\nonumber \right. \\ {}&\qquad \left. {\left( \frac{1}{2}\overline{\left( \frac{f''(z)}{f'(z)}+\frac{1}{z}\right) }-\frac{\overline{f'(z)}f(z)}{1+|f(z)|^2}\right) \varphi }\right\rangle =0, \end{aligned}$$

which implies in particular that $\textrm{Im}\,\left( \langle \Delta \varphi ,\varphi \rangle \right) =0$. Then, we compute

4.26 $$\begin{aligned} \langle \partial _{\overline{z}}\varphi ,\overline{\varphi }\rangle&=\left\langle \left( \frac{1}{2}\overline{\left( \frac{f''(z)}{f'(z)}+\frac{1}{z}\right) }-\frac{\overline{f'(z)}f(z)}{1+|f(z)|^2}\right) \varphi ,\overline{\varphi }\right\rangle \nonumber \\&=\left( \overline{\left( \frac{f''(z)}{f'(z)}+\frac{1}{z}\right) }-2\frac{\overline{f'(z)}f(z)}{1+|f(z)|^2}\right) . \end{aligned}$$

Therefore, we have

$$\begin{aligned} \frac{1}{4}\langle \Delta \varphi ,\overline{\varphi }\rangle&=\partial _{z}\langle \partial _{\overline{z}}\varphi ,\overline{\varphi }\rangle -\langle \partial _{\overline{z}}\varphi ,\partial _{z}\overline{\varphi }\rangle =\partial _{z}\langle \partial _{\overline{z}}\varphi ,\overline{\varphi }\rangle -|\partial _{\overline{z}}\varphi |^2, \end{aligned}$$

where we used $\partial _{z}\overline{\varphi }=\overline{\partial _{\overline{z}}\varphi }$. By (4.26), we deduce that

$$\begin{aligned} \partial _{z}\langle \partial _{\overline{z}}\varphi ,\overline{\varphi }\rangle =-2\frac{|f'(z)|^2}{1+|f(z)|^2}+2\frac{|f'(z)|^2|f(z)|^2}{(1+|f(z)|^2)^2}=-\frac{2|f'(z)|^2}{(1+|f(z)|^2)^2}, \end{aligned}$$

so that

$$\begin{aligned} \langle \Delta \varphi ,\overline{\varphi }\rangle =-\frac{8|f'(z)|^2}{(1+|f(z)|^2)^2}-4|\partial _{\overline{z}}\varphi |^2\in \mathbb {R}, \end{aligned}$$

which implies that

4.27 $$\begin{aligned} \textrm{Im}\,\left( \langle \Delta \varphi ,\overline{\varphi }\rangle \right) =0. \end{aligned}$$

Therefore, we deduce that (4.24) holds, which implies that $\vec {u}$ and $\vec {v}$ solve the equations (4.21).

**Step 4.** Proof of the decomposition $\omega _1=*\,d(G_{\Omega _1}+\mu _1)$.

Recall that, $\vec {u}=\vec {u}_1$ and $\vec {v}=\vec {v}_1$, and let

$$\begin{aligned} \omega =\langle \vec {u},d\vec {v}\,\rangle =\langle \vec {u},\partial \vec {v}\,\rangle +\langle \vec {u},\overline{\partial }\vec {v}\,\rangle . \end{aligned}$$

Recall that since $*\, dx=dy$ and $*\, dy=-dx$, we have

$$\begin{aligned} *\, dz&=*\left( dx+i\,dy\right) =dy-i\,dx=-i(dx+i\,dy)=-i\,dz\\ *\, d\overline{z}&=i\,d\overline{z}. \end{aligned}$$

Therefore, $\omega =*\,d\left( \mu +G\right)$ (where we write for simplicity $G=G_{\mathbb {D}}=\log |\,\cdot \,|$) if and only if

$$\begin{aligned} \langle \vec {u},\partial \vec {v}\,\rangle +\langle \vec {u},\overline{\partial }\vec {v}\,\rangle =*\left( \partial \left( \mu +G\right) +\overline{\partial }\left( \mu +G\right) \right) =-i\,\partial (\mu +G)+i\,\overline{\partial }(\mu +G), \end{aligned}$$

which is equivalent to the identity

4.28 $$\begin{aligned} \langle \vec {u},\partial \vec {v}\,\rangle =-i\,\partial \left( \mu +G\right) . \end{aligned}$$

We have, by (4.14) and (4.15)

$$\begin{aligned} \partial _{z}\vec {v}&=\partial _{z}\text {Re}\,(\varphi )=\frac{1}{2}\left( \partial _{z}\varphi +\overline{\partial _{\overline{z}}\varphi }\right) =\frac{1}{2}\left( -\frac{1}{2}\left( \frac{f''(z)}{f'(z)}+\frac{1}{z}\right) \varphi -\frac{f'(z)\overline{f(z)}}{1+|f(z)|^2}\varphi \right. \\ {}&\quad \left. +\frac{2f'(z)}{1+|f(z)|^2}\chi (z)(-f(z),i\,f(z),1) +\frac{1}{2}{\left( \frac{f''(z)}{f'(z)}+\frac{1}{z}\right) }\overline{\varphi }-\frac{f'(z)\overline{f(z)}}{1+|f(z)|^2}\overline{\varphi }\right) \\ {}&=-\frac{i}{2}\left( \frac{f''(z)}{f'(z)}+\frac{1}{z}\right) \text {Im}(\varphi )-\frac{1}{2}\frac{f'(z)\overline{f(z)}}{1+|f(z)|^2}\text {Re}(\varphi )\\ {}&\quad +\frac{f'(z)}{1+|f(z)|^2}\chi (z)\left( -f(z),i\,f(z),1\right) . \end{aligned}$$

Therefore, using (4.16), (4.17), (4.18), and $\langle \vec {u},\vec {v}\rangle =0$, we deduce that

$$\begin{aligned} \langle \vec {u},\partial _{z}\vec {v}\,\rangle&=\langle \textrm{Im}\,(\varphi ),\partial _{z}\textrm{Re}\,(\varphi )\rangle =-\frac{i}{2}\left( \frac{f''(z)}{f'(z)}+\frac{1}{z}\right) \\&\quad +\frac{i}{2}\frac{f'(z)}{1+|f(z)|^2}\langle (-f(z),i\,f(z),1),\overline{\psi }\rangle \\&=-\frac{i}{2}\left( \frac{f''(z)}{f'(z)}-\frac{2f'(z)\overline{f(z)}}{1+|f(z)|^2}+\frac{1}{z}\right) , \end{aligned}$$

and this concludes the proof of (4.28) since by (4.6)

$$\begin{aligned} \partial _{z}(\mu (z)+\log |z|)&=\partial _{z}\left( \log |f'(z)|-\log (1+|f(z)|^2)-\frac{1}{2}\log (2)+\log |z|\right) \\&=\frac{1}{2}\left( \frac{f''(z)}{f'(z)}-\frac{2f'(z)\overline{f(z)}}{1+|f(z)|^2}+\frac{1}{z}\right) . \end{aligned}$$

This last identity concludes the proof of the theorem. $\quad \square$

Finally, we will establish the uniqueness of distributional solutions of the system (4.3) with appropriate boundary conditions (4.5). This is the exact analog of Remark I.1 of [5]. First, we need to define explicit maps that yield trivialisations of vector fields on simply connected domains of the sphere. Let $\Omega _1\subset S^2$ be as Theorem 4.3. Using the stereographic projection $\pi : S^2\setminus \left\{ N\right\} \rightarrow \mathbb {C}$, we have one holomorphic chart *z* on $S^2\setminus \left\{ N\right\}$, and for a domain $\Omega _1\subset S^2\setminus \left\{ N\right\}$, it yields a trivialisation $T \Omega _1 \rightarrow \Omega \times \mathbb {C}$, where $\Omega = \pi (\Omega _1)$.

More explicitly, let $X:\Omega \rightarrow \mathbb {R}^3$ be a vector field such that $\langle X,\vec {n}\hspace{0.1em}\rangle =0$, where $\vec {n}:\Omega \rightarrow S^2$ is the unit normal given by

$$\begin{aligned} \pi ^{-1}(z)=\vec {n}(z)=\left( \frac{2\,\textrm{Re}\,(z)}{1+|z|^2},\frac{2\,\textrm{Im}\,(z)}{1+|z|^2},\frac{-1+|z|^2}{1+|z|^2}\right) . \end{aligned}$$

Now, we introduce the function $\psi :\mathbb {C}\rightarrow \mathbb {C}^3$, given by

$$\begin{aligned} \psi (z)=\left( \frac{1-z^2}{1+|z|^2},\frac{i(1+z^2)}{1+|z|^2},\frac{2z}{1+|z|^2}\right) , \end{aligned}$$

and we easily check that

4.29 $$\begin{aligned} \langle \psi ,\psi \rangle =0\qquad |\psi |^2=\langle \psi ,\overline{\psi }\rangle =2. \end{aligned}$$

Therefore, we deduce that $(\vec {u}_1,\vec {u}_2)$ defined as follows is a tangent unit moving frame (orthogonal to $\vec {n}$ )

$$\begin{aligned} \vec {u}_1(z)=\textrm{Re}\,(\psi (z)),\quad \vec {u}_2(z)=\textrm{Im}\,(\psi (z)). \end{aligned}$$

The trivialisation map on $\Omega _1\subset S^2\setminus \left\{ N\right\}$ is then given by

4.30 $$\begin{aligned} T\Omega _{1}&\rightarrow \Omega \times \mathbb {C}\nonumber \\ (z,v)&\mapsto (z,\langle v,\vec {u}_1(z)\rangle +i\,\langle v,\vec {u}_2(z)\rangle ), \end{aligned}$$

while the trivialisation map of sections is given by

4.31 $$\begin{aligned} \Psi _{\Omega _1}:\Gamma (T\Omega _1)&\rightarrow C^{\infty }(\Omega _1,\mathbb {C})\nonumber \\ X&\mapsto \langle X,\vec {u}_1\rangle +i\,\langle X,\vec {u}_2\rangle . \end{aligned}$$

Notice that for all tangent vector field *X*, we have $\langle X,\vec {n}\hspace{0.1em}\rangle =0$, which implies that there exists real functions $\lambda _1,\lambda _2 :\Omega _1\rightarrow \mathbb {R}$ such that

$$\begin{aligned} X=\lambda _1\vec {u}_1+\lambda _2\vec {u}_2. \end{aligned}$$

### Remark 4.5

Using the next Theorem 4.6, it is easy to check that $(\vec {u}_j,\vec {v}_j)$ ($j=1,2$) are harmonic vector fields since by (4.13) and (4.14), we have

$$\begin{aligned} -\Delta \chi =\left| \frac{f''(z)}{f'(z)}+\frac{1}{z}\right| ^2\chi =|\nabla \chi |^2\chi , \end{aligned}$$

*i.e.* $\chi :\mathbb {D}\rightarrow S^1$ is a harmonic map with values into $S^1$.

### Theorem 4.6

Under the conditions of Theorem 4.3, let $\Omega _1^{*}=\Omega _1\setminus \left\{ p_1\right\}$ and $\vec {u}\in W^{1,2}_{\textrm{loc}}(\Omega _1^{*},U\Omega _1)\cap W^{1,1}(\Omega _1,U\Omega _1)$ be a unit vector field in $\Omega _1$, and let $\vec {u}_0=\Psi _{\Omega _1}(\vec {u}):\Omega _1\rightarrow S^1$. Then $\vec {u}$ is a harmonic vector field on $\Omega _1$, *i.e.*

$$\begin{aligned} -\Delta _{g_0}\vec {u}=|d\vec {u}\hspace{0.1em}|_{g_0}^2{\vec {u}}+\left( 2\langle d\vec {u},d\vec {n}\hspace{0.1em}\rangle _{g_0}+\langle \vec {u},\Delta _{g_0}\vec {n}\hspace{0.1em}\rangle \right) \vec {n}\end{aligned}$$

if and only if $\vec {u}_0$ is a harmonic map with values into $S^1$, *i.e.*

$$\begin{aligned} -\Delta _{g_0}\vec {u}_0=|d\vec {u}_0|^2_{g_0}\vec {u}_0. \end{aligned}$$

In particular, for all degree 1 boundary data $h\in H^{1/2}(\partial \Omega _1, S^1)$ and $p\in \Omega _1$, there exists a unique unit vector-field $\vec {u}\in W^{1,2}_{\textrm{loc}}(\Omega _1^{*},U\Omega _1)\cap W^{1,1}(\Omega _1,U\Omega _1)$ such that $\vec {u}=\Psi _{\Omega _1}^{-1}(h)$ on $\partial \Omega _1$ and such that $\vec {u}_0=\Psi _{\Omega _1}(\vec {u})$ satisfies in the distributional sense

$$\begin{aligned} {{\,\textrm{div}\,}}\left( \vec {u}_0\times \nabla \vec {u}_0\right) =0\qquad \text {in}\;\,\mathscr {D}'(\Omega _1). \end{aligned}$$

### Remark 4.7

If $\vec {u}_0:\Omega \rightarrow S^1$, writing locally $\vec {u}_0=e^{i\varphi }$ for some real-valued function $\varphi$, we deduce that $\vec {u}_0$ is harmonic if and only if

$$\begin{aligned} -\Delta \vec {u}_0= \left( |\nabla \varphi |^2-i\,(\Delta \varphi )\right) \vec {u}_0=|\nabla \vec {u}_0|^2\vec {u}_0. \end{aligned}$$

Therefore, $\vec {u}_0$ is harmonic as a map with values into $S^1$ if and only if $\varphi$ is harmonic, *i.e.* $\Delta \varphi =0$.

### Proof of Theorem 4.6

By making a stereographic projection, thanks to the conformal invariance of the harmonic equation, we deduce that for all unit vector-field $\vec {u}\in \Gamma (T\Omega _1^{*})$ is given in $\Omega =\pi _N(\Omega _1)$ as

4.32 $$\begin{aligned} \vec {u}=\lambda _1\,\textrm{Re}\,(\psi )+\lambda _2\,\textrm{Im}\,(\psi ), \end{aligned}$$

where

$$\begin{aligned} \psi (z)=\left( \frac{1-z^2}{1+|z|^2},\frac{i(1+z^2)}{1+|z|^2},\frac{2z}{1+|z|^2}\right) . \end{aligned}$$

Furthermore, we have $\lambda _1^2+\lambda _2^2=1$, which implies that there exists a measurable function $\varphi$ such that $\lambda _1+i\lambda _2=e^{-i\varphi }$. In particular, we can rewrite (4.32) as

$$\begin{aligned} \vec {u}&=\cos (\varphi )\,\textrm{Re}\,(\psi )-\sin (\varphi )\,\textrm{Im}\,(\psi )=\textrm{Re}\,(e^{-i\varphi })\,\textrm{Re}\,(\psi )+\textrm{Im}\,(e^{-i\varphi })\,\textrm{Im}\,(\psi )\\&=\textrm{Re}\,\left( e^{i\varphi }\psi \right) , \end{aligned}$$

where we used the identity $\textrm{Re}\,(a)\,\textrm{Re}\,(b)+\textrm{Im}\,(a)\,\textrm{Im}\,(b)=\textrm{Re}\,(\overline{a}\,b)$ valid for all $a,b\in \mathbb {C}$. If

$$\begin{aligned} \vec {v}=\sin (\varphi )\textrm{Re}\,(\psi )+\cos (\varphi )\textrm{Im}\,(\psi )=\textrm{Im}\,(e^{i\varphi }\psi ), \end{aligned}$$

we immediately have $\langle \vec {u},\vec {v}\hspace{0.1em}\rangle =0$, and since $|\vec {u}\hspace{0.1em}|^2=|\vec {v}\hspace{0.1em}|^2 = 1$, while $-\Delta \vec {n}=|\nabla \vec {n}\hspace{0.1em}|^2\vec {n}$, we get

$$\begin{aligned}&\langle \Delta \vec {u},\vec {u}\hspace{0.1em}\rangle =-|\nabla \vec {u}\hspace{0.1em}|^2\\&\langle \Delta \vec {u},\vec {n}\hspace{0.1em}\rangle =-2\langle \nabla \vec {u},\nabla \vec {n}\hspace{0.1em}\rangle -\langle \vec {u},\Delta \vec {n}\hspace{0.1em}\rangle =-2\langle \nabla \vec {u},\nabla \vec {n}\hspace{0.1em}\rangle , \end{aligned}$$

and similar formulae for $\vec {v}$. Therefore, we deduce that $(\vec {u},\vec {v})$ solves the system

$$\begin{aligned} \left\{ \begin{aligned} -\Delta \vec {u}&=|\nabla \vec {u}\hspace{0.1em}|^2\vec {u}+2\langle \nabla \vec {u},\nabla \vec {n}\hspace{0.1em}\rangle \vec {n}\qquad{} & {} \text {in}\;\,\Omega \\ -\Delta \vec {v}&=|\nabla \vec {v}\hspace{0.1em}|^2\vec {v}+2\langle \nabla \vec {v},\nabla \vec {n}\hspace{0.1em}\rangle \vec {n}\qquad{} & {} \text {in}\;\,\Omega , \end{aligned}\right. \end{aligned}$$

if and only if

$$\begin{aligned} \langle \Delta \vec {u},\vec {v}\hspace{0.1em}\rangle =\langle \Delta \vec {v},\vec {u}\hspace{0.1em}\rangle =0. \end{aligned}$$

Now, we compute

$$\begin{aligned} \Delta \vec {u}&=\textrm{Re}\,\left( (i\,\Delta \varphi -|\nabla \varphi |^2)e^{i\varphi }\psi \right) +2\,\textrm{Re}\,\left( ie^{i\varphi }\nabla \varphi \cdot \nabla \psi \right) +\textrm{Re}\,\left( e^{i\varphi }\Delta \psi \right) \\&=-(\Delta \varphi )\textrm{Im}\,(e^{i\varphi }\psi )-|\nabla \varphi |^2\textrm{Re}\,(e^{i\varphi }\psi )+\textrm{Re}\,\left( e^{i\varphi }\Delta \psi \right) \\&=-(\Delta \varphi )\,\vec {v}-|\nabla \varphi |^2\vec {u}+2\,\textrm{Re}\,\left( ie^{i\varphi }\nabla \varphi \cdot \nabla \psi \right) +\textrm{Re}\,(e^{i\varphi }\Delta \psi )\\ \Delta \vec {v}&=(\Delta \varphi )\,\vec {u}-|\nabla \varphi |^2\vec {u}+2\,\textrm{Im}\,\left( ie^{i\varphi }\nabla \varphi \cdot \nabla \psi \right) +\textrm{Im}\,(e^{i\varphi }\Delta \psi ). \end{aligned}$$

We have since $\langle \nabla \varphi ,\varphi \rangle =0$ the identity

$$\begin{aligned} \left\langle \textrm{Re}\,(ie^{i\varphi }\nabla \varphi \cdot \nabla \psi ),\vec {v}\hspace{0.1em}\right\rangle&=\textrm{Re}\,\left\langle ie^{i\varphi }\nabla \varphi \cdot \nabla \psi ,\frac{e^{i\varphi }\psi -e^{-i\varphi }\overline{\psi }}{2i}\right\rangle \\&=-\frac{1}{2}\textrm{Re}\,\left( \nabla \varphi \cdot \langle \nabla \psi ,\overline{\psi }\rangle \right) \\ \left\langle \textrm{Im}\,(ie^{i\varphi }\nabla \varphi \cdot \nabla \psi ),\vec {u}\right\rangle&=\textrm{Im}\,\left\langle ie^{i\varphi }\nabla \varphi \cdot \nabla \psi ,\frac{e^{i\varphi }\psi +e^{-i\varphi }\overline{\psi }}{2}\right\rangle \\&=\frac{1}{2}\textrm{Re}\,\left( \nabla \varphi \cdot \langle \nabla \psi ,\overline{\psi }\rangle \right) \\ \langle \textrm{Re}\,(e^{i\varphi }\Delta \psi ),\vec {v}\hspace{0.1em}\rangle&=\textrm{Re}\,\left\langle e^{i\varphi }\Delta \psi ,\frac{e^{i\varphi }\psi -e^{-i\varphi }\psi }{2i}\right\rangle \\&=\frac{1}{2}\textrm{Im}\,\left( e^{2i\varphi }\langle \Delta \psi ,\psi \rangle \right) -\frac{1}{2}\textrm{Im}\,\langle \Delta \psi ,\psi \rangle \\ \langle \textrm{Im}\,(e^{i\varphi }\Delta \psi ),\vec {u}\rangle&=\frac{1}{2}\textrm{Im}\,\left( e^{2i\varphi }\langle \Delta \psi ,\psi \rangle \right) +\frac{1}{2}\textrm{Im}\,\langle \Delta \psi ,\overline{\psi }\rangle . \end{aligned}$$

In particular, we have

$$\begin{aligned} \langle \Delta \vec {u},\vec {v}\hspace{0.1em}\rangle =-(\Delta \varphi )-\textrm{Re}\,\left( \nabla \varphi \cdot \langle \nabla \psi ,\overline{\psi }\rangle \right) +\frac{1}{2}\textrm{Im}\,\left( e^{2i\varphi }\langle \Delta \psi ,\psi \rangle \right) -\frac{1}{2}\textrm{Im}\,\langle \Delta \psi ,\psi \rangle \\ \langle \Delta \vec {v},\vec {u}\rangle =(\Delta \varphi )+\textrm{Re}\,\left( \nabla \varphi \cdot \langle \nabla \psi ,\overline{\psi }\rangle \right) +\frac{1}{2}\textrm{Im}\,\left( e^{2i\varphi }\langle \Delta \psi ,\psi \rangle \right) +\frac{1}{2}\textrm{Im}\,\langle \Delta \psi ,\overline{\psi }\rangle . \end{aligned}$$

Summing those equations and substracting the first one to the second one yields the system

4.33 $$\begin{aligned} \left\{ \begin{aligned}&\textrm{Im}\,\left( e^{2i\varphi }\langle \Delta \psi ,\psi \rangle \right) =0\\&2(\Delta \varphi )+2\,\textrm{Re}\,\left( \nabla \varphi \cdot \langle \nabla \psi ,\overline{\psi }\rangle \right) +\textrm{Im}\,\langle \Delta \psi ,\overline{\psi }\rangle =0. \end{aligned} \right. \end{aligned}$$

We will show that for all smooth *real-valued* function $\varphi :\Omega \rightarrow \mathbb {R}$

4.34 $$\begin{aligned}&\textrm{Re}\,\left( \nabla \varphi \cdot \langle \nabla \psi ,\overline{\psi }\rangle \right) =\langle \Delta \psi ,\psi \rangle =\textrm{Im}\,\langle \Delta \psi ,\overline{\psi }\rangle =0, \end{aligned}$$

which will imply that $(\vec {u},\vec {v})$ solves the system (4.33) if and only if $\Delta \varphi =0$, or $\varphi$ is harmonic.

Now, we compute

$$\begin{aligned} \partial _{z}\psi&=-\frac{\overline{z}}{1+|z|^2}\psi +\frac{2}{1+|z|^2}\left( -z,i\,z,1\right) \\ \partial _{\overline{z}}\psi&=-\frac{z}{1+|z|^2}\psi . \end{aligned}$$

We have

$$\begin{aligned} \nabla \varphi \cdot \nabla \psi&=2\,\partial _{z}\varphi \cdot \partial _{\overline{z}}\psi +2\,\partial _{\overline{z}}\varphi \cdot \partial _{z}\psi \\&=-2\frac{\overline{z}\partial _{z}\varphi }{1+|z|^2}\psi +\frac{4}{1+|z|^2}(-z\partial _{z}\varphi ,iz\partial _{z}\varphi ,\partial _{z}\varphi ) -2\frac{z\partial _{\overline{z}}\varphi }{1+|z|^2}\psi \\&=-4\,\textrm{Re}\,\left( \frac{\overline{z}\partial _{z}\varphi }{1+|z|^2}\right) \psi +\frac{4}{1+|z|^2}(-z\partial _{z}\varphi ,iz\partial _{z}\varphi ,\partial _{z}\varphi ). \end{aligned}$$

Then we have

4.35 $$\begin{aligned} \frac{1}{4}\Delta \psi =\partial _{z\overline{z}}^2\psi =\frac{-1+|z|^2}{(1+|z|^2)^2}\psi -\frac{2}{(1+|z|^2)^2}(-z^2,iz^2,z). \end{aligned}$$

Now, notice that

$$\begin{aligned} \left\langle (-z^2,iz^2,z),\psi \right\rangle&=\frac{1}{1+|z|^2}\left\langle (-z^2,iz^2,z),\left( 1-z^2,i(1+z^2),2z\right) \right\rangle \\ {}&=\frac{1}{1+|z|^2}\left( -z^2(1-\overline{z}^2)+z^2(1+\overline{z}^2)+2z^2\right) =0, \end{aligned}$$

which implies as $\langle \psi ,\psi \rangle =0$ and by (4.35) that

4.36 $$\begin{aligned} \langle \Delta \psi ,\psi \rangle =0. \end{aligned}$$

Now, we have

$$\begin{aligned} \langle (-z^2,iz^2,z),\overline{\psi }\rangle =\frac{1}{1+|z|^2}\left( -z^2(1-\overline{z}^2)+z^2(1+\overline{z}^2)+2|z|^2\right) =2|z|^2. \end{aligned}$$

Since $|\psi |^2=\langle \psi ,\overline{\psi }\rangle =2$, we deduce that

4.37 $$\begin{aligned} \langle \Delta \psi ,\overline{\psi }\rangle =4\left( \frac{2(-1+|z|^2)}{(1+|z|^2)^2}-\frac{4|z|^2}{(1+|z|^2)^2}\right) =-\frac{8}{1+|z|^2}\in \mathbb {R}. \end{aligned}$$

We now compute

$$\begin{aligned} \langle (-z,iz,1),\overline{\psi }\rangle =\frac{1}{1+|z|^2}\left( -z(1-\overline{z}^2)+z(1+\overline{z}^2)+2\overline{z}\right) =2\overline{z}. \end{aligned}$$

which shows since $|\psi |^2=2$ that

$$\begin{aligned} \langle \partial _{z}\psi ,\psi \rangle&=-\frac{2\overline{z}}{1+|z|^2}+\frac{4\overline{z}}{1+|z|^2}=\frac{2\overline{z}}{1+|z|^2};\\ \langle \partial _{\overline{z}}\psi ,\psi \rangle&=-\frac{2z}{1+|z|^2}. \end{aligned}$$

Therefore, we have

$$\begin{aligned} \nabla \varphi \cdot \langle \nabla \psi ,\psi \rangle&=2\,\partial _{\overline{z}}\varphi \cdot \langle \partial _{z}\psi ,\psi \rangle +2\,\partial _{z}\varphi \cdot \langle \partial _{\overline{z}}\psi ,\psi \rangle =\frac{4\,\overline{z}\,\partial _{\overline{z}}\varphi }{1+|z|^2}-\frac{4\,z\,\partial _{z}\varphi }{1+|z|^2}\\&=8i\,\textrm{Im}\,\left( \frac{\overline{z}\,\partial _{\overline{z}}\varphi }{1+|z|^2}\right) \in i\mathbb {R}, \end{aligned}$$

and this immediately implies that

4.38 $$\begin{aligned} \textrm{Re}\,\left( \nabla \varphi \cdot \langle \nabla \psi ,\psi \rangle \right) =0. \end{aligned}$$

Finally, we deduce by (4.36), (4.37) and (4.38) that (4.34) holds and that the system (4.33) holds if and only if $\Delta \varphi =0$. If $\vec {u}=g_{\Omega _1}=\Psi _{\Omega _1}(g)$ for some $g:\partial \Omega _1=\Gamma \rightarrow S^1$, then we have

$$\begin{aligned} \lambda _1+i\lambda _2=g, \end{aligned}$$

or

$$\begin{aligned} e^{-i\varphi }=g\qquad \text {on}\;\, \Gamma . \end{aligned}$$

In particular, the function $\vec {u}_0=e^{-i\varphi }:\Omega _1\setminus \left\{ p_1\right\} \rightarrow S^1$ is a harmonic map on $\Omega _1\setminus \left\{ p_1\right\}$ satisfying $\vec {u}_0=h$ on $\Gamma$. Now, notice that provided $\vec {u}\in W^{1,1}(\Omega _1)$, one can rewrite the equation distributionally as

$$\begin{aligned} {{\,\textrm{div}\,}}\left( \vec {u}\times \nabla \vec {u}\hspace{0.1em}\right) =2\langle \nabla \vec {u},\nabla \vec {n}\hspace{0.1em}\rangle \vec {n}\times \vec {u}. \end{aligned}$$

In particular, since $u_0$ is harmonic, we deduce that

$$\begin{aligned} {{\,\textrm{div}\,}}\left( \vec {u}_0\times \nabla \vec {u}_0\right) =\frac{\partial }{\partial x_1}\left( \vec {u}_0\times \frac{\partial \vec {u}_0}{\partial x_1}\right) +\frac{\partial }{\partial x_2}\left( \vec {u}_0\times \frac{\partial \vec {u}_0}{\partial x_2}\right) =0. \end{aligned}$$

By Theorem I.5 and Remark I.1 of [5], we deduce that $\vec {u}_0$ is the unique harmonic function with a singularity at $p_1$ such that $\vec {u}_0=h$ on $\partial \Omega _1$. This concludes the proof of the theorem. $\quad \square$

## Proof of the Main Theorems for Non-smooth Curves

In order to extend Theorem 3.6 to the non-smooth setting, we will obtain another formula for $\mathscr {E}_0$ in terms of conformal maps and that holds true for any closed simple curve of finite Loewner energy. Using this additional formula, the convergence result will be easily obtained.

Under the preceding notations, if $\Gamma \subset S^2$ Weil–Petersson quasicircle, from Remark 3.4, thanks to Theorem 4.3, there exists harmonic moving frames $(\vec {u}_1,\vec {v}_1)$ and $(\vec {u}_2,\vec {v}_2)$ on $\Omega _1$ and $\Omega _2$ with arbitrary singularities $p_1$ and $p_2$, respectively, such that

$$\begin{aligned} \mathscr {E}(\Gamma )&=\int _{\Omega _1}|d\mu _1|^2_{g_0}d\textrm{vol}_{g_0}+\int _{\Omega _2}|d\mu _2|^2_{g_0}d\textrm{vol}_{g_0}\\&\quad +2\int _{\Omega _1}G_{\Omega _1}K_{g_0}d\textrm{vol}_{g_0}+2\int _{\Omega _2}G_{\Omega _2}K_{g_0}d\textrm{vol}_{g_0}+4\pi . \end{aligned}$$

where $\omega _j=\langle \vec {u}_j,d\vec {v}_j\rangle =*\,d(G_{\Omega _j}+\mu _j)$ in $\mathscr {D}'(\Omega _j)$ for $j=1,2$, and $\mu _j$ satisfies (3.10). We saw in Theorem 3.6 that in the case of smooth curves, there exists conformal maps $f_1:\mathbb {D}\rightarrow \Omega _1$ and $f_2:\mathbb {D}\rightarrow \Omega _2$ such that

$$\begin{aligned} I^L(\Gamma )=\frac{1}{\pi }\mathscr {E}(\Gamma )+4\log |\nabla f_1(0)|+4\log |\nabla f_2(0)|-12\log (2)=\frac{1}{\pi }\mathscr {E}_0(\Gamma ). \end{aligned}$$

In this section, we generalise this result to curves of finite Loewner energy. Now, if $\pi :S^2\setminus \left\{ p_2\right\} \rightarrow \mathbb {C}$ is a stereographic projection, since $f_j:\mathbb {D}\rightarrow \Omega _j$ is conformal and $\pi$ is also conformal, we deduce that $\pi \circ f_j:\mathbb {D}\rightarrow \pi (\Omega _j)\subset \mathbb {R}$ is also conformal. Therefore, these maps are biholomorphic or anti-biholomorphic, so up to a complex conjugate (which is an isometry), we can assume that they are holomorphic. Notice that $\Omega =\pi (\Omega _1)$ is bounded, while $\pi (\Omega _2)=\mathbb {C}\setminus \overline{\Omega }$ is unbounded. Therefore, if ${\mathfrak {i}}:\mathbb {C}\setminus \left\{ 0\right\} \rightarrow \mathbb {C}\setminus \left\{ 0\right\}$ is the inversion, we let $g=\pi \circ f_{2} \circ {\mathfrak {i}}:\mathbb {C}\setminus \overline{\mathbb {D}}\rightarrow \mathbb {C}\setminus \overline{\Omega }$ and $f ={\pi \circ f_1}:\mathbb {D}\rightarrow \Omega$. From (1.3) and (1.4), if $\gamma =\pi (\Gamma )$, we have

5.1 $$\begin{aligned} I^L(\Gamma )&=I^L(\gamma )=\int _{\mathbb {D}}\left| \frac{f''(z)}{f'(z)}\right| ^2|dz|^2+\int _{\mathbb {C}\setminus \overline{\mathbb {D}}}\left| \frac{g''(z)}{g'(z)}\right| ^2|dz|^2\nonumber \\&\quad +4\pi \log |f'(0)|-4\pi \log |g'(\infty )|. \end{aligned}$$

Indeed, since $f_2(0)=p_2$, we have $g(\infty )=\infty$, so that the functions *f*, *g* satisfy the needed conditions to apply Theorem 1.1.

Now, with the previous notations, define the functional

$$\begin{aligned} \mathscr {E}_0({\Gamma })=\mathscr {E}({\Gamma })+4\pi \log |\nabla f_1(0)|+4\pi \log |\nabla f_2(0)|-12\pi \log (2). \end{aligned}$$

### Definition 5.1

Let $\gamma$ be a Jordan curve with finite Loewner energy. Let $f:\mathbb {D}\rightarrow \Omega$, $g:\mathbb {C}\setminus \overline{\mathbb {D}}\rightarrow \mathbb {C}\setminus \overline{\Omega }$ be biholomorphic maps such that $g(\infty )=\infty$, we define the third universal Liouville action $S_3$ by

$$\begin{aligned} S_3(\gamma )&=\int _{\mathbb {D}}\left| \frac{f''(z)}{f'(z)}-2\frac{f'(z)}{f(z)}\frac{|f(z)|^2}{1+|f(z)|^2}\right| ^2|dz|^2\\&\quad +\int _{\mathbb {C}\setminus \overline{{\mathbb {D}}}}\left| \frac{g''(z)}{g'(z)}-2\frac{g'(z)}{g(z)}\frac{|g(z)|^2}{1+|g(z)|^2}+\frac{2}{z}\right| ^2|dz|^2\\&\quad +2\int _{\mathbb {D}}\log |z|\frac{4|f'(z)|^2|dz|^2}{(1+|f(z)|^2)^2}-2\int _{\mathbb {C}\setminus \overline{\mathbb {D}}}\log |z|\frac{4|g'(z)|^2|dz|^2}{(1+|g(z)|^2)^2}+4\pi \\&\quad +4\pi \log |f'(0)|-4\pi \log |g'(\infty )|-4\pi \log (1+|f(0)|^2). \end{aligned}$$

### Remark 5.2

1. One may wonder about the origin of this formula. It will be made clear in the proof of the next theorem where we explicitly rewrite $\mathscr {E}_0$ with the help of the conformal maps *f* and *g* defined above.
2. We call this quantity $S_3$ since a functional called $S_2$ was defined in [53] as the log-determinant of the Grunsky operator associated with the curve $\gamma$ (up to a factor $-\frac{1}{12}$).

The goal of this section is to show the identity

5.2 $$\begin{aligned} {\pi } I^L = S_1=S_3=\mathscr {E}_0. \end{aligned}$$

The third equality is straightforward and is proved in Theorem 5.3, and the proof of the whole identity is completed in Theorem 5.5.

### Theorem 5.3

Let $\Gamma \subset S^2$ be a simple curve of finite Loewner energy. Then we have

$$\begin{aligned} \mathscr {E}_0(\Gamma )=S_3(\Gamma ). \end{aligned}$$

### Proof

If $\Gamma \subset S^2$ is a curve of finite Loewner energy $\Omega _1$ and $\Omega _2$ the two connected components of $S^2\setminus \Gamma$, and $f_1:\mathbb {D}\rightarrow \Omega _1$, $f_2:\mathbb {D}\rightarrow \Omega _2$ are the conformal maps associated to $\Gamma$ in the definition of $\mathscr {E}$ with $f_j (0) = p_j$ for $j= 1,2$. Now, recall from (3.6) that

$$\begin{aligned} \log |\nabla f_1|=\frac{1}{2}\log (2)+\mu _1\circ f_1. \end{aligned}$$

We have, by conformal invariance of the Dirichlet energy,

5.3 $$\begin{aligned} \int _{\mathbb {D}}\left| \nabla \log |\nabla f_1|\right| ^2|dz|^2=\int _{\mathbb {D}}\left| \nabla (\mu _1\circ f_1)\right| ^2|dz|^2=\int _{\Omega _1}|d\mu _1|_{g_0}^2d\textrm{vol}_{g_0}. \end{aligned}$$

Since $f_1$ is conformal and $f_1(0) = p_1$, we have

$$\begin{aligned} G_{\Omega _1} \circ f_1(z) = G_{\Omega _1,p_1} \circ f_1 (z) = G_{\mathbb D,0} (z) = \log |z|. \end{aligned}$$

A change of variable gives

5.4 $$\begin{aligned} 2\int _{\Omega _1}G_{\Omega _1}d\textrm{vol}_{g_0}=\int _{\mathbb {D}}\log |z||\nabla f_1|^2|dz|^2. \end{aligned}$$

Finally, we deduce by (5.3) and (5.4) that

5.5 $$\begin{aligned} \mathscr {E}_0({\Gamma })&=\int _{\Omega _1}|d\mu _1|^2_{g_0}d\textrm{vol}_{g_0}+\int _{\Omega _2}|d\mu _2|^2_{g_0}d\textrm{vol}_{g_0}+2\int _{\Omega _1}G_{\Omega _1}K_{g_0}d\textrm{vol}_{g_0}\nonumber \\&\quad +2\int _{\Omega _2}G_{\Omega _2}K_{g_0}d\textrm{vol}_{g_0}+4\pi \nonumber \\&\quad +4\pi \log |\nabla f_1(0)|+4\pi \log |\nabla f_2(0)|-12\pi \log (2)\nonumber \\&=\int _{\mathbb {D}}|\nabla \log |\nabla f_1||^2|dz|^2+\int _{\mathbb {D}}|\nabla \log |\nabla f_2||^2|dz|^2+\int _{\mathbb {D}}\log |z||\nabla f_1|^2|dz|^2\nonumber \\&\quad +\int _{\mathbb {D}}\log |z||\nabla f_2|^2|dz|^2+4\pi \nonumber \\&\quad +4\pi \log |\nabla f_1(0)|+4\pi \log |\nabla f_2(0)|-12\pi \log (2). \end{aligned}$$

Up to a rotation of $S^2$, we can assume that $p_2=N$ and if $\pi :S^2\setminus \left\{ N\right\} \rightarrow \mathbb {C}$ is the standard stereographic projection, let

$$\begin{aligned} f&=\pi \circ f_1:\mathbb {D}\rightarrow \Omega \\ \widetilde{g}&=\pi \circ f_2:\mathbb {D}\rightarrow \mathbb {C}\setminus \overline{\Omega } \end{aligned}$$

which we assume without loss of generality to be biholomorphic (up to a complex conjugation). Now, since

$$\begin{aligned} f_1(z)=\pi ^{-1}(f(z))=\left( \frac{2\,\textrm{Re}\,(f(z))}{1+|f(z)|^2},\frac{2\,\textrm{Im}\,(f(z))}{1+|f(z)|^2},\frac{-1+|f(z)|^2}{1+|f(z)|^2}\right) , \end{aligned}$$

a computation shows that

$$\begin{aligned} \partial _{z}f_1=f'\left( \frac{(1-\overline{f}^2)}{(1+|f|^2)^2},\frac{-i(1+\overline{f}^2)}{(1+|f|^2)^2},\frac{2\overline{f}}{(1+|f|^2)^2}\right) , \end{aligned}$$

which implies that

$$\begin{aligned} |\partial _{z}f_1|^2=\frac{|f'|^2}{(1+|f|^2)^4}\left( |1-f^2|^2+|1+f^2|^2+4|f|^2\right) =\frac{2|f'|^2}{(1+|f|^2)^2}. \end{aligned}$$

We deduce that

5.6 $$\begin{aligned} |\nabla f_1|^2=4|\partial _{z}f_1|^2=\frac{8|f'|^2}{(1+|f|^2)^2}. \end{aligned}$$

Therefore, we have

$$\begin{aligned} \log |\nabla f_1|=\log |f'|-\log (1+|f|^2)+\frac{3}{2}\log (2), \end{aligned}$$

so that

5.7 $$\begin{aligned}&4\pi \log |\nabla f_1(0)|=4\pi \log |f'(0)|-4\pi \log \left( 1+|f(0)|^2\right) +6\pi \log (2) \end{aligned}$$

Since $\Omega =f(\mathbb {D})$ is bounded, we have

5.8 $$\begin{aligned} \int _{\mathbb {D}}|f'(z)|^2\frac{|f(z)|^2|dz|^2}{(1+|f(z)|^2)^2}\le \frac{1}{4}\int _{\mathbb {D}}|f'(z)|^2|dz|^2=\frac{1}{4}\textrm{Area}(\Omega )<\infty . \end{aligned}$$

Therefore, (5.8) implies that $\nabla \log |\nabla f_1|\in L^{2}(\mathbb {D})$ and

5.9 $$\begin{aligned} \int _{\mathbb {D}}|\nabla \log |\nabla f_1||^2|dz|^2=\int _{\mathbb {D}}\left| \frac{f''(z)}{f'(z)}-2\frac{f'(z)}{f(z)}\frac{|f(z)|^2}{1+|f(z)|^2}\right| ^2|dz|^2<\infty , \end{aligned}$$

while (5.6) implies that

5.10 $$\begin{aligned} \left| \int _{\mathbb {D}}\log |z||\nabla f_1|^2|dz|^2\right| =-\int _{\mathbb {D}}\log |z|\frac{8|f'(z)|^2|dz|^2}{(1+|f(z)|^2)^2}<\infty , \end{aligned}$$

which is finite by (5.8) and the smoothness of *f* in $\mathbb {D}$. Since the function $\widetilde{g}:\mathbb {D}\rightarrow \mathbb {C}\setminus \overline{\Omega }$ is unbounded at 0, we need to check that

$$\begin{aligned} \int _{\mathbb {D}}\left| \frac{\widetilde{g}''(z)}{\widetilde{g}'(z)}-2\frac{\widetilde{g}'(z)\overline{\widetilde{g}(z)}}{1+|\widetilde{g}(z)|^2}\right| ^2|dz|^2=\int _{\mathbb {D}}\left| \frac{\widetilde{g}''(z)}{\widetilde{g}'(z)}-2\frac{\widetilde{g}'(z)}{g(z)}\frac{|\widetilde{g}(z)|^2}{1+|\widetilde{g}(z)|^2}\right| ^2|dz|^2<\infty . \end{aligned}$$

For this, as $\widetilde{g}$ is univalent and $\widetilde{g}(0)=\infty$, we deduce that $\widetilde{g}$ admits the following meromorphic expansion at $z=0$ for some $a\in \mathbb {C}\setminus \left\{ 0\right\}$ and $a_0,a_1\in \mathbb {C}$

5.11 $$\begin{aligned} \widetilde{g}(z)=\frac{a}{z}+a_0+a_1z+O(|z|^2). \end{aligned}$$

Therefore, we have, by a direct computation,

5.12 $$\begin{aligned} \frac{\widetilde{g}''(z)}{\widetilde{g}'(z)}-2\frac{\widetilde{g}'(z)}{\widetilde{g}(z)}\frac{|\widetilde{g}(z)|^2}{1+|\widetilde{g}(z)|^2}&=-\frac{a_0}{a}+\left( \frac{a_0^2}{a^2}-\frac{4a_1}{a}\right) z-\frac{\overline{z}}{|a|^2}\nonumber \\&\quad +O(|z|^2)\in L^{\infty }_{\textrm{loc}}(\mathbb {D}). \end{aligned}$$

Since $\Gamma$ is a Weil–Petersson quasicircle, we deduce by estimates similar to (5.8) and (5.9) that $\nabla \log |\widetilde{g}'|\in L^2(\mathbb {D}\setminus \overline{\mathbb {D}}(0,\varepsilon ))$ and ${\widetilde{g}}' \in L^2(\mathbb {D}\setminus \overline{\mathbb {D}}(0,\varepsilon ))$ for all $\varepsilon >0$ and we finally deduce that

$$\begin{aligned} \int _{\mathbb {D}}\left| \frac{\widetilde{g}''(z)}{\widetilde{g}'(z)}-2\frac{\widetilde{g}'(z)}{\widetilde{g}(z)}\frac{|\widetilde{g}(z)|^2}{1+|\widetilde{g}(z)|^2}\right| ^2|dz|^2<\infty . \end{aligned}$$

Now, if ${g}=\widetilde{g}\circ {\mathfrak {i}}:\mathbb {C}\setminus \overline{\mathbb {D}}\rightarrow \mathbb {C}\setminus \overline{\Omega }$, we compute and

$$\begin{aligned} \frac{\widetilde{g}''(z)}{\widetilde{g}'(z)}-2\frac{\widetilde{g}'(z)}{\widetilde{g}(z)}\frac{|\widetilde{g}(z)|^2}{1+|\widetilde{g}(z)|^2}&=-\frac{1}{z^2}\left( \frac{{g}''(1/z)}{{g}'(1/z)}-2\frac{{g}'(1/z)}{{g}(1/z)}\frac{|{g}(1/z)|^2}{1+|{g}(1/z)|^2}+2z\right) . \end{aligned}$$

A change of variable shows that

5.13 $$\begin{aligned}&\int _{\mathbb {D}}\left| \frac{\widetilde{g}''(z)}{\widetilde{g}'(z)}-2\frac{\widetilde{g}'(z)}{\widetilde{g}(z)}\frac{|\widetilde{g}(z)|^2}{1+|\widetilde{g}(z)|^2}\right| ^2|dz|^2\nonumber \\&\quad =\int _{\mathbb {C}\setminus \overline{\mathbb {D}}}\left| \frac{{g}''(z)}{{g}'(z)}-2\frac{{g}'(z)}{{g}(z)}\frac{|{g}(z)|^2}{1+|{g}(z)|^2}+\frac{2}{z}\right| ^2|dz|^2 \end{aligned}$$

Furthermore, we directly get that

5.14 $$\begin{aligned} \int _{\mathbb {D}}\log |z|\frac{|\widetilde{g}'(z)|^2|dz|^2}{(1+|\widetilde{g}(z)|^2)^2}&=\int _{\mathbb {D}}\log |z|\frac{|{g}'(1/z)|^2}{(1+|{g}(1/z)|^2)^2}\frac{|dz|^2}{|z|^4}\nonumber \\&=\int _{\mathbb {C}\setminus \overline{\mathbb {D}}}\log \left( \frac{1}{|z|}\right) \frac{|{g}'(z)|^2|dz|^2}{(1+|{g}(z)|^2)^2}\nonumber \\&=-\int _{\mathbb {C}\setminus \overline{\mathbb {D}}}\log |z|\frac{|g'(z)|^2|dz|^2}{(1+|g(z)|^2)^2}. \end{aligned}$$

Now, notice that

$$\begin{aligned} |\nabla f_2|^2=\frac{8|g'(z)|^2}{(1+|g(z)|^2)^2}=\frac{8}{|a|^2}+O(|z|), \end{aligned}$$

which implies that

$$\begin{aligned} \log |\nabla f_2|=\frac{3}{2}\log (2)-\log |a|. \end{aligned}$$

Furthermore, the expansion (5.11) shows that as $|z|\rightarrow \infty$, we have

$$\begin{aligned} {g}(z)=az+O(1), \end{aligned}$$

so that $|a|=|{g}'(\infty )|$, and

5.15 $$\begin{aligned} 4\pi \log |\nabla f_2(0)|=-4\pi \log |{g}'(\infty )|+6\pi \log (2). \end{aligned}$$

Finally, we deduce, by (5.7) and (5.15), that

5.16 $$\begin{aligned}&4\pi \log |\nabla f_1(0)|+4\pi \log |\nabla f_2(0)|-12\pi \log (2)\nonumber \\ {}&\quad =4\pi \log |f'(0)|-4\pi \log |{g}'(\infty )|-4\pi \log \left( 1+|f(0)|^2\right) . \end{aligned}$$

Gathering (5.5), (5.9), (5.10), (5.13), (5.14), and (5.16), we finally deduce that

$$\begin{aligned} \mathscr {E}_0(\Gamma )&=\int _{\mathbb {D}}|\nabla \log |\nabla f_1||^2|dz|^2+\int _{\mathbb {D}}|\nabla \log |\nabla f_2||^2|dz|^2+\int _{\mathbb {D}}\log |z||\nabla f_1|^2|dz|^2\\&\quad +\int _{\mathbb {D}}\log |z||\nabla f_2|^2|dz|^2+4\pi \nonumber \\&\quad +4\pi \log |\nabla f_1(0)|+4\pi \log |\nabla f_2(0)|-12\pi \log (2)\\&=\int _{\mathbb {D}}\left| \frac{f''(z)}{f'(z)}-2\frac{f'(z)}{f(z)}\frac{|f(z)|^2}{1+|f(z)|^2}\right| ^2|dz|^2\\&\quad +\int _{\mathbb {C}\setminus \overline{{\mathbb {D}}}}\left| \frac{{g}''(z)}{{g}'(z)}-2\frac{{g}'(z)}{{g}(z)}\frac{|{g}(z)|^2}{1+|{g}(z)|^2}+\frac{2}{z}\right| ^2|dz|^2\\&\quad +2\int _{\mathbb {D}}\log |z|\frac{4|f'(z)|^2|dz|^2}{(1+|f(z)|^2)^2}-2\int _{\mathbb {C}\setminus \overline{\mathbb {D}}}\log |z|\frac{4|{g}'(z)|^2|dz|^2}{(1+|{g}(z)|^2)^2}+4\pi \\&\quad +4\pi \log |f'(0)|-4\pi \log |\widetilde{g}'(\infty )|-4\pi \log (1+|f(0)|^2)\\&=S_3(\Gamma ) \end{aligned}$$

which concludes the proof of the theorem. $\quad \square$

### Remark 5.4

If $\Gamma =S^1$, then we can take $f=\textrm{Id}_{\mathbb {D}}$ and $g=\textrm{Id}_{\mathbb {C}\setminus \overline{\mathbb {D}}}$, and we compute

$$\begin{aligned} S_3(\Gamma )&=\int _{\mathbb {D}}\left| \frac{f''(z)}{f'(z)}-2\frac{f'(z)}{f(z)}\frac{|f(z)|^2}{1+|f(z)|^2}\right| ^2|dz|^2\\&\quad +\int _{\mathbb {C}\setminus \overline{{\mathbb {D}}}}\left| \frac{g''(z)}{g'(z)}-2\frac{g'(z)}{g(z)}\frac{|g(z)|^2}{1+|g(z)|^2}+\frac{2}{z}\right| ^2|dz|^2\\&\quad +2\int _{\mathbb {D}}\log |z|\frac{4|f'(z)|^2|dz|^2}{(1+|f(z)|^2)^2}-2\int _{\mathbb {C}\setminus \overline{\mathbb {D}}}\log |z|\frac{4|g'(z)|^2|dz|^2}{(1+|g(z)|^2)^2}+4\pi \\&\quad +4\pi \log |f'(0)|-4\pi \log |g'(\infty )|-4\pi \log (1+|f(0)|^2)\\&=8\int _{\mathbb {D}}\frac{|z|^2|dz|^2}{(1+|z|^2)^2}+16\int _{\mathbb {D}}\log |z|\frac{|z|^2|dz|^2}{(1+|z|^2)^2}+4\pi \\&=16\pi \int _{0}^1\frac{r^3dr}{(1+r^2)^2}+32\pi \int _{0}^1\frac{r\log (r)dr}{(1+r^2)^2}+4\pi \\&=16\pi \left( \frac{1}{4}(2\log (2)-1)\right) +32\pi \left( -\frac{1}{4}\log (2)\right) +4\pi \\&=0 \end{aligned}$$

as expected.

In the next theorem, we finally complete the proof of (5.2) by showing that $\pi I^L(\Gamma )=S_3(\Gamma )$.

### Theorem 5.5

**Fig. 2:**
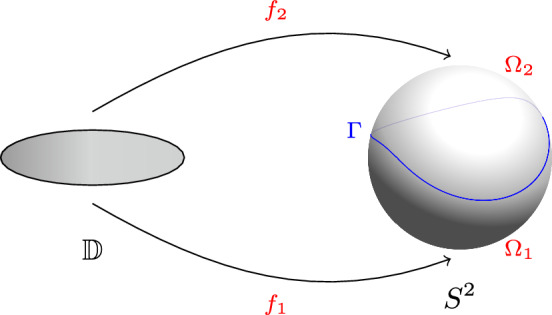
Spherical formula for the Loewner energy with respect to conformal maps

Let $\Gamma \subset S^2$ be a closed simple curve of finite Loewner energy. Then we have

$$\begin{aligned} I^L(\Gamma )=\frac{1}{\pi }\mathscr {E}_0(\Gamma ), \end{aligned}$$

where $\mathscr {E}_0$ is defined in (1.12). Furthermore, if $\Omega _1,\Omega _2\subset S^2\setminus \Gamma$ are the two connected components of $S^2\setminus \Gamma$, for all conformal maps $f_1:\mathbb {D}\rightarrow \Omega _1$ and $f_2:\mathbb {D}\rightarrow \Omega _2$, we have (Fig. 2)

$$\begin{aligned} I^L(\Gamma )&=\frac{1}{\pi }\sum _{j=1}^{2}\left( \int _{\mathbb {D}}|\nabla \log |\nabla f_j||^2|dz|^2+\int _{\mathbb {D}}\log |z||\nabla f_j|^2|dz|^2+\textrm{Area}(\Omega _j) \right. \\&\quad \left. +4\pi \log |\nabla f_j(0)|\right) -12\log (2) \end{aligned}$$

### Proof

By Theorem 3.6, we have the identity $I^L(\Gamma )=\frac{1}{\pi }\mathscr {E}_0(\Gamma )$ for all smooth $\Gamma$, and by the preceding Theorem 5.3, we have $\mathscr {E}_0(\Gamma )=S_3(\Gamma )$ for any Jordan curve $\Gamma$ of finite Loewner energy. Therefore, we will prove that $I^L=\frac{1}{\pi } S_3$ which will imply our result.

We now let $\Omega _1,\Omega _2\subset S^2\setminus {\Gamma }$ be the two connected components of $S^2\setminus \Gamma$, and $f_1:\mathbb {D}\rightarrow \Omega _1$, $f_2:\mathbb {D}\rightarrow \Omega _2$ be the two conformal maps associated to $\Omega _1$ and $\Omega _2$, and let $p_1=f_1(0)$ and $p_2=f_2(0)$. Up to a rotation on $S^2$ (which does not change any of the energies considered), we can assume that $p_2=N$. If $\pi :S^2\setminus \left\{ N\right\} \rightarrow \mathbb {C}$ is the standard stereographic projection, let $\gamma =\pi (\Gamma )$, and $\Omega$ the bounded component of $\mathbb {C}\setminus \gamma$ and define $f=\pi \circ f_1:\mathbb {D}\rightarrow \pi (\Omega _1)=\Omega$ and $g=\pi \circ f_2\circ {\mathfrak {i}}:\mathbb {C}\setminus \overline{\mathbb {D}}\rightarrow \mathbb {C}\setminus \overline{\Omega }$ such that (using Theorem 5.3)

5.17 $$\begin{aligned} \mathscr {E}_0(\Gamma )&=S_3(\gamma )=\int _{\mathbb {D}}\left| \frac{f''(z)}{f'(z)}-2\frac{f'(z)}{f(z)}\frac{|f(z)|^2}{1+|f(z)|^2}\right| ^2|dz|^2\nonumber \\&\quad +\int _{\mathbb {C}\setminus \overline{{\mathbb {D}}}}\left| \frac{g''(z)}{g'(z)}-2\frac{g'(z)}{g(z)}\frac{|g(z)|^2}{1+|g(z)|^2}+\frac{2}{z}\right| ^2|dz|^2\nonumber \\&\quad +2\int _{\mathbb {D}}\log |z|\frac{4|f'(z)|^2|dz|^2}{(1+|f(z)|^2)^2}-2\int _{\mathbb {C}\setminus \overline{\mathbb {D}}}\log |z|\frac{4|g'(z)|^2|dz|^2}{(1+|g(z)|^2)^2}+4\pi \nonumber \\&\quad +4\pi \log |f'(0)|-4\pi \log |g'(\infty )|-4\pi \log (1+|f(0)|^2). \end{aligned}$$

Now, let $\left\{ \gamma _n\right\} _{n\in \mathbb {N}}$ be a sequence of smooth curves converging uniformly to a simple curve $\gamma$, and $f_n:\mathbb {D}\rightarrow \mathbb {C}$ be a sequence of biholomorphic maps such that $f_n(0)=0$, $f_n'(0)=1$ and $f_n(\mathbb {D})=\Omega _n$, where $\Omega _n$ is the bounded component of $\mathbb {C}\setminus \gamma _n$. Thanks to Corollary A.4 of [53] and Theorem 8.1 [59], the following convergence result holds:

$$\begin{aligned} \lim \limits _{n\rightarrow \infty }\int _{\mathbb {D}}\left| \frac{f_n''(z)}{f_n'(z)}-\frac{f''(z)}{f'(z)}\right| ^2|dz|^2=0. \end{aligned}$$

Here $f:\mathbb {D}\rightarrow \Omega$ is a univalent function such that $f(0)=0$ and $f'(0)=1$. Therefore, we deduce that

5.18 $$\begin{aligned} I^L(\Gamma _n)\underset{n\rightarrow \infty }{\longrightarrow }I^L(\Gamma ). \end{aligned}$$

In particular, for any sequence of holomorphic maps $g_n:\mathbb {C}\setminus \overline{\mathbb {D}}\rightarrow \mathbb {C}\setminus \overline{\Omega _n}$ such that $g_n(\infty )=\infty$, since

$$\begin{aligned} S_1(\Gamma _n)&=\pi \, I^L(\Gamma _n)=\int _{\mathbb {D}}\left| \frac{f''_n(z)}{f_n'(z)}\right| ^2|dz|^2+\int _{\mathbb {C}\setminus \overline{\mathbb {D}}}\left| \frac{g_n''(z)}{g_n'(z)}\right| ^2|dz|^2\\&\quad +4\pi \log |f_n'(0)|-4\pi \log |g_n'(\infty )|\\&=\int _{\mathbb {D}}\left| \frac{f''_n(z)}{f_n'(z)}\right| ^2|dz|^2+\int _{\mathbb {C}\setminus \overline{\mathbb {D}}}\left| \frac{g_n''(z)}{g_n'(z)}\right| ^2|dz|^2-4\pi \log |g_n'(\infty )|, \end{aligned}$$

we deduce that

5.19 $$\begin{aligned} \int _{\mathbb {C}\setminus \overline{\mathbb {D}}}\left| \frac{g_n''(z)}{g_n'(z)}\right| ^2|dz|^2-4\pi \log |g_n'(\infty )|\underset{n\rightarrow \infty }{\longrightarrow }\int _{\mathbb {C}\setminus \overline{\mathbb {D}}}\left| \frac{g''(z)}{g'(z)}\right| ^2|dz|^2-4\pi \log |g'(\infty )| \end{aligned}$$

for all univalent function $g:\mathbb {C}\setminus \overline{\mathbb {D}}\rightarrow \mathbb {C}\setminus \overline{\Omega }$ such that $g(\infty )=\infty$. Now, if $\gamma =\pi (\Gamma )\subset \mathbb {C}$, let $\left\{ \varepsilon _n\right\} _{n\in \mathbb {N}}\subset (0,\infty )$ such that $\varepsilon _n\underset{n\rightarrow \infty }{\longrightarrow }0$, and define

$$\begin{aligned} f_n:\mathbb {D}&\rightarrow \mathbb {C}\\ z&\mapsto f((1-\varepsilon _n)z)/{(1-\varepsilon _n)}. \end{aligned}$$

Then $\gamma _n=f_n(S^1)$ is smooth and uniformly converges to $\gamma$. Furthermore, we have

5.20 $$\begin{aligned} \int _{\mathbb {D}}\left| \frac{f''_n(z)}{f'_n(z)}-\frac{f''(z)}{f'(z)}\right| ^2|dz|^2\underset{n\rightarrow \infty }{\longrightarrow }0. \end{aligned}$$

which implies that

$$\begin{aligned} I^L(\gamma _n)=\frac{1}{\pi }S_1(\gamma _n)\underset{n\rightarrow \infty }{\longrightarrow }\frac{1}{\pi }S_1(\gamma )=I^L(\gamma ). \end{aligned}$$

Now, we need to show the result $f_n'\underset{n\rightarrow { \infty }}{\longrightarrow }f'$ in $L^2(\mathbb {D})$ strongly. Notice that since $f'$ is smooth in $\mathbb {D}$, we have by construction $f_n'\underset{n\rightarrow \infty }{\longrightarrow }f'$ almost everywhere. Furthermore, a linear change of variable shows that

$$\begin{aligned} \int _{\mathbb {D}}|f_n'(z)|^2|dz|^2&=\int _{\mathbb {D}}|f'((1-\varepsilon _n)z))|^2|dz|^2\\&=\frac{1}{(1-\varepsilon _n)^2}\int _{\mathbb {D}(0,1-\varepsilon _n)}|f'(w)|^2|dw|^2 \underset{n\rightarrow \infty }{\longrightarrow }\int _{\mathbb {D}}|f'(w)|^2|dw|^2. \end{aligned}$$

By Brezis–Lieb lemma ([11]), since $f_n'\underset{n\rightarrow \infty }{\longrightarrow }f'$ almost everywhere and $\left\| f_n'\right\| _{\textrm{L}^{2}(\mathbb {D})}\underset{n\rightarrow \infty }{\longrightarrow }\left\| f'\right\| _{\textrm{L}^{2}(\mathbb {D})}$, we deduce that

5.21 $$\begin{aligned} f_n'\underset{n\rightarrow \infty }{\longrightarrow } f'\quad \text {strongly in}\;\, L^2(\mathbb {D}). \end{aligned}$$

Therefore, we also get the convergence

$$\begin{aligned} \begin{aligned} \frac{f_n'}{f_n}\frac{|f_n|^2}{1+|f_n|^2}&\underset{n\rightarrow \infty }{\longrightarrow } \frac{f'}{f}\frac{|f|^2}{1+|f|^2}\quad{} & {} \text {in}\;\, L^2(\mathbb {D}) \end{aligned} \end{aligned}$$

which finally shows by (5.20) that

5.22 $$\begin{aligned}&\int _{\mathbb {D}}\left| \frac{f_n''(z)}{f_n'(z)}-2\frac{f_n'(z)}{f_n(z)}\frac{|f_n(z)|^2}{1+|f_n(z)|^2}\right| ^2|dz|^2\nonumber \\&\quad \underset{n\rightarrow \infty }{\longrightarrow }\int _{\mathbb {D}}\left| \frac{f''(z)}{f'(z)}-2\frac{f'(z)}{f(z)}\frac{|f(z)|^2}{1+|f(z)|^2}\right| ^2|dz|^2\nonumber \\&\int _{\mathbb {D}}\log |z|\frac{4|f_n'(z)|^2|dz|^2}{(1+|f_n(z)|^2)^2}\underset{n\rightarrow \infty }{\longrightarrow }\int _{\mathbb {D}}\log |z|\frac{4|f'(z)|^2|dz|^2}{(1+|f(z)|^2)^2}. \end{aligned}$$

Finally, we also have $f_n(0)=f(0)$ and

5.23 $$\begin{aligned} 4\pi \log |f_n'(0)|=4\pi \log |f'(0)|+4\pi \log (1-\varepsilon _n)\underset{n\rightarrow \infty }{\longrightarrow }4\pi \log |f'(0)|. \end{aligned}$$

Therefore, if $\Omega _n=f_n(\mathbb {D})$, and $g_n:\mathbb {C}\setminus \overline{\mathbb {D}}\rightarrow \mathbb {C}\setminus \overline{\Omega _n}$ is any univalent map such that $g_n(\infty )=\infty$, since $\gamma _n\underset{n\rightarrow \infty }{\longrightarrow }\gamma$ uniformly, we can assume without loss of generality that $g_n'(\infty )\underset{n\rightarrow \infty }{\longrightarrow }g'(\infty )$. Furthermore, by Corollary A.4 of [53], we also get

5.24 $$\begin{aligned}&\lim \limits _{n\rightarrow \infty }\int _{\mathbb {C}\setminus \overline{\mathbb {D}}}\left| \frac{g_n''(z)}{g_n'(z)}-\frac{g''(z)}{g(z)}\right| ^2=0 \end{aligned}$$

5.25 $$\begin{aligned}&\lim \limits _{n\rightarrow \infty }\int _{\mathbb {C}\setminus \overline{\mathbb {D}}}\left| \left( \frac{g_n''(z)}{g_n'(z)}-2\frac{g_n'(z)}{g_n(z)}+\frac{2}{z}\right) -\left( \frac{g''(z)}{g'(z)}-2\frac{g'(z)}{g(z)}+\frac{2}{z}\right) \right| ^2|dz|^2=0. \end{aligned}$$

As previously, we have

5.26 $$\begin{aligned} \frac{g_n'}{g_n}\frac{1}{1+|g_n|^2}&\underset{n\rightarrow \infty }{\longrightarrow }\frac{g'}{g}\frac{1}{1+|g|^2}\quad \text {in}\;\, L^2(\mathbb {C}\setminus \overline{\mathbb {D}})\nonumber \\ {g_n'}\frac{1}{1+|g_n|^2}&\underset{n\rightarrow \infty }{\longrightarrow }{g'}\frac{1}{1+|g|^2}\quad \text {in}\;\, L^2(\mathbb {C}\setminus \overline{\mathbb {D}}) \end{aligned}$$

Therefore, (5.24) and (5.26) imply that

5.27 $$\begin{aligned}&\int _{\mathbb {C}\setminus \overline{{\mathbb {D}}}}\left| \frac{g_n''(z)}{g_n'(z)}-2\frac{g_n'(z)}{g_n(z)}\frac{|g_n(z)|^2}{1+|g_n(z)|^2}+\frac{2}{z}\right| ^2|dz|^2\nonumber \\&\quad \underset{n\rightarrow \infty }{\longrightarrow }\int _{\mathbb {C}\setminus \overline{{\mathbb {D}}}}\left| \frac{g''(z)}{g'(z)}-2\frac{g'(z)}{g(z)}\frac{|g(z)|^2}{1+|g(z)|^2}+\frac{2}{z}\right| ^2|dz|^2\nonumber \\&\int _{\mathbb {C}\setminus \overline{{\mathbb {D}}}}\log |z|\frac{4|g_n'(z)|^2|dz|^2}{(1+|g_n(z)|^2)^2}\nonumber \\&\quad \underset{n\rightarrow \infty }{\longrightarrow }\int _{\mathbb {C}\setminus \overline{{\mathbb {D}}}}\log |z|\frac{4|g'(z)|^2|dz|^2}{(1+|g(z)|^2)^2}. \end{aligned}$$

Finally, we deduce by (5.17), (5.22), (5.23) and (5.27) that

$$\begin{aligned} S_3(\gamma _n)\underset{n\rightarrow \infty }{\longrightarrow }S_3(\gamma ) \end{aligned}$$

which concludes the proof of the theorem by (5.18), Theorem 3.6 and Theorem 5.3. $\quad \square$

### Remarks 5.6

Notice that we can also directly express the Loewner energy using moving frames. First, we trivially have

$$\begin{aligned} I^L(\Gamma )&=\frac{1}{\pi }\left\{ \sum _{i=1}^2\int _{\Omega _i}|\omega _i-*\,dG_{\Omega _i}|_{g_0}^2+2\int _{\Omega _i}G_{\Omega _i}K_{g_0}d\textrm{vol}_{g_0}+\textrm{Area}(\Omega _i)\right\} \\&\quad +4\log |\nabla f_1(0)|+4\log |\nabla f_2(0)|-12\,\log (2). \end{aligned}$$

Alternatively, we have

$$\begin{aligned} I^L(\Gamma )&=\frac{1}{2 { \pi }}\int _{\Omega _1}\left( |d\vec {u}_1|^2_{g_0}+|d\vec {v}_1|^2_{g_0}-2|dG_{\Omega _1}|^2_{g_0}\right) d\textrm{vol}_{g_0}\\&\quad +\frac{1}{2 {\pi }}\int _{\Omega _2}\left( |d\vec {u}_2|^2_{g_0}+|d\vec {v}_2|^2_{g_0}-2|dG_{\Omega _2}|^2_{g_0}\right) d\textrm{vol}_{g_0}\nonumber \\&\quad +4\log |\nabla f_1(0)|+4\log |\nabla f_2(0)|-12\log (2), \end{aligned}$$

which is (up to the second line involving the conformal maps $f_1$ and $f_2$) very reminiscent of the Ginzburg–Landau renormalised energy ([5, Chapter VIII]).

To see this equality, since $\vec {u}_1$, $\vec {v}_1$ and $\vec {n}$ are unitary, we have

$$\begin{aligned} |d\vec {u}_1|^2_{g_0}&=|\langle d\vec {u}_1,\vec {v}_1\rangle |_{g_0}^2+|\langle d\vec {u}_1,\vec {n}\hspace{0.1em}\rangle |_{g_0}^2=|\omega _1|^2_{g_0}+|\langle d\vec {n}\hspace{0.1em},\vec {u}_1\rangle |_{g_0}^2\\ |d\vec {v}_1|^2_{g_0}&=|\omega _1|^2_{g_0}+|\langle d\vec {n},\vec {v}_1\hspace{0.1em}\rangle |_{g_0}^2\\ |d\vec {u}_1|^2_{g_0}+|d\vec {v}_1|_{g_0}^2&=2|\omega _1|^{2}_{g_0}+|d\vec {n}\hspace{0.1em}|_{g_0}^2=2|\omega _1|_{g_0}^2+2. \end{aligned}$$

Then, integrating by parts and using that $G_{\Omega _1}=0$ on $\partial \Omega _1$, we deduce by Stokes theorem—and the equation (that follows from (4.10))

$$\begin{aligned} d\left( \omega _1-*\,dG_{\Omega _1}\right) =-K_{g_0}d\textrm{vol}_{g_0}, \end{aligned}$$

where $K_{g_0}=1$ is the Gauss curvature of the sphere—that

$$\begin{aligned}&\frac{1}{2}\int _{\Omega _1}\left( |d\vec {u}_1|^2_{g_0}+|d\vec {v}_1|_{g_0}^2-2|dG_{\Omega _1}|_{g_0}^2\right) d\textrm{vol}_{g_0}\\&\quad =\int _{\Omega _1}\left( |\omega _1|_{g_0}^2-|dG_{\Omega _1}|^2_{g_0}\right) d\textrm{vol}_{g_0}+\textrm{Area}_{g_0}(\Omega _1)\\&\quad =\int _{\Omega _1}\langle \omega _1-*\, dG_{\Omega _1},\omega _1+*\, dG_{\Omega _1}\rangle _{g_0}d\textrm{vol}_{g_0}+\textrm{Area}_{g_0}(\Omega _1)\\&\quad =\int _{\Omega _1}|\omega _1-*\, dG_{\Omega _1}|_{g_0}^2d\textrm{vol}_{g_0}+2\int _{\Omega _1}(\omega _1-*\, dG_{\Omega _1})\wedge dG_{\Omega _1}+\textrm{Area}_{g_0}(\Omega _1)\\&\quad =\int _{\Omega _1}|\omega _1-*\,dG_{\Omega _1}|_{g_0}^2 {d\textrm{vol}_{g_0}}-2\int _{\Omega _1}G_{\Omega _1}d(\omega _1-*\,dG_{\Omega _1})+\textrm{Area}_{g_0}(\Omega _1)\\&\quad =\int _{\Omega _1}|d\mu _1|_{g_0}^2d\textrm{vol}_{g_0}+2\int _{\Omega _1}G_{\Omega _1}K_{g_0}d\textrm{vol}_{g_0}+\textrm{Area}_{g_0}(\Omega _1) \end{aligned}$$

which implies since $\textrm{Area}_{g_0}(S^2)=4\pi$ that

$$\begin{aligned}&\frac{1}{2}\int _{\Omega _1}\left( |d\vec {u}_1|^2_{g_0}+|d\vec {v}_1|^2_{g_0}-2|dG_{\Omega _1}|^2_{g_0}\right) d\textrm{vol}_{g_0}\\&\qquad +\frac{1}{2}\int _{\Omega _1}\left( |d\vec {u}_2|^2_{g_0}+|d\vec {v}_2|^2_{g_0}-2|dG_{\Omega _2}|^2_{g_0}\right) d\textrm{vol}_{g_0}\\&\quad =\int _{\Omega _1}|d\mu _1|_{g_0}^2d\textrm{vol}_{g_0}+\int _{\Omega _2}|d\mu _2|_{g_0}^2+2\int _{\Omega _1}G_{\Omega _1}K_{g_0}d\textrm{vol}_{g_0}\\&\qquad +2\int _{\Omega _2}G_{\Omega _2}K_{g_0}d\textrm{vol}_{g_0}+4\pi . \end{aligned}$$

Notice that it gives another explanation for the factor $4\pi$ in the definition of $\mathscr {E}$.

## Appendix

In this appendix, we provide more details on the geodesic curvature for Weil–Petersson quasicircles and show a consequence of Theorem 5.5 which is an identity on univalent functions associated to a Weil–Petersson quasicircle.

### Properties of the Geodesic Curvature for Weil–Petersson Quasicircles

#### Lemma 6.1

Let $\mathbb {H}=\mathbb {C}\cap \left\{ z:\textrm{Im}\,(z)>0\right\}$ be the Poincaré half-plane, and $f:\mathbb {H}\rightarrow \mathbb {C}$ be a univalent holomorphic map, $\Omega =f(\mathbb {H})$, and assume that $\gamma =\partial \Omega$ is a simple curve of finite Loewner energy. Then the geodesic curvature $k_{g_0}$ of $\gamma$ is given in the distributional sense by

6.1 $$\begin{aligned} k_{g_0}=\textrm{Im}\,\left( \frac{f''(z)}{f'(z)}\right) \qquad \text {for all}\;\, z\in \partial _{\infty }\mathbb {H}=\mathbb {R}. \end{aligned}$$

#### Proof

The geodesic curvature is given by

$$\begin{aligned} k_{g_0}=\langle \partial _{x}\vec {u},\vec {v}\rangle , \end{aligned}$$

if $(\vec {u},\vec {v})$ is the Cartesian frame given by (in the following formulae, *f* is seen as a $\mathbb {R}^2$-valued function)

$$\begin{aligned} \left\{ \begin{aligned} \vec {u}&=\frac{\partial _{x}f}{|\partial _{x}f|}=\left( \textrm{Re}\,\left( \frac{f'(z)}{|f'(z)|}\right) ,\textrm{Im}\,\left( \frac{f'(z)}{|f'(z)|}\right) \right) =\frac{f'(z)}{|f'(z)|}\\ \vec {v}&=\frac{\partial _{y}f}{|\partial _{y}f|}=\left( -\textrm{Im}\,\left( \frac{f'(z)}{|f'(z)|}\right) ,\textrm{Re}\,\left( \frac{f'(z)}{|f'(z)|}\right) \right) =i\,\frac{f'(z)}{|f'(z)|}. \end{aligned}\right. \end{aligned}$$

Define $\vec {u}_z=\dfrac{f'(z)}{|f'(z)|}$. Then we have

$$\begin{aligned} \partial _{z}\vec {u}_z&=\partial _{z}\left( \frac{f'(z)}{|f'(z)|}\right) =\frac{f''(z)}{|f'(z)|}-\frac{1}{2}\frac{f''(z)}{|f'(z)|}=\frac{1}{2}\frac{f''(z)}{|f'(z)|}=\frac{1}{2}\frac{f''(z)}{f'(z)}\vec {u}_z\\ \partial _{\overline{z}}\vec {u}_z&=\partial _{\overline{z}}\left( \frac{f'(z)}{|f'(z)|}\right) =-\frac{1}{2}\frac{f'(z)^2\overline{f''(z)}}{|f'(z)|^3}=-\frac{1}{2}\overline{\left( \frac{f''(z)}{f'(z)}\right) }\vec {u}_z. \end{aligned}$$

Therefore, we deduce that

$$\begin{aligned} \partial _{z}\textrm{Re}\,\left( \frac{f'(z)}{|f'(z)|}\right)&=\frac{1}{2}\left( \partial _{z}\left( \frac{f'(z)}{|f'(z)|}\right) +\overline{\partial _{\overline{z}}\left( \frac{{f'(z)}}{|f'(z)|}\right) }\right) =\frac{1}{4}\frac{f''(z)}{f'(z)}\left( \vec {u}_z-\vec {u}_{\overline{z}}\right) \\&=\frac{i}{2}\frac{f''(z)}{f'(z)}\textrm{Im}\,\left( \frac{f'(z)}{|f'(z)|}\right) \\ \partial _{z}\textrm{Im}\,\left( \frac{f'(z)}{|f'(z)|}\right)&=-\frac{i}{2}\left( \frac{1}{2}\frac{f''(z)}{|f'(z)|}+\frac{1}{2} \overline{\frac{f''(z)}{|f'(z)|}}\right) =-\frac{i}{4}\frac{f''(z)}{f'(z)}\left( \vec {u}_z+\vec {u}_{\overline{z}}\right) \\&=-\frac{i}{2}\frac{f''(z)}{f'(z)}\textrm{Re}\,\left( \frac{f'(z)}{|f'(z)|}\right) . \end{aligned}$$

Therefore, we have

$$\begin{aligned} \partial _{x}\textrm{Re}\,\left( \frac{f'(z)}{|f'(z)|}\right)&=2\,\textrm{Re}\,\left( \partial _{z}\textrm{Re}\,\left( \frac{f'(z)}{|f'(z)|}\right) \right) =-\textrm{Im}\,\left( \frac{f''(z)}{f'(z)}\right) \textrm{Im}\,\left( \frac{f'(z)}{|f'(z)|}\right) \\ \partial _{x}\textrm{Im}\,\left( \frac{f'(z)}{|f'(z)|}\right)&=\textrm{Im}\,\left( \frac{f''(z)}{f'(z)}\right) \textrm{Re}\,\left( \frac{f'(z)}{f'(z)}\right) , \end{aligned}$$

so that $\partial _{x}\vec {u}=\textrm{Im}\,\left( \dfrac{f''(z)}{f'(z)}\right) \vec {v},$ and

$$\begin{aligned} k_{g_0}=\langle \partial _{x}\vec {u},\vec {v}\rangle =\textrm{Im}\,\left( \frac{f''(z)}{f'(z)}\right) , \end{aligned}$$

which concludes the proof of the lemma. $\quad \square$

#### Lemma 6.2

Let $\gamma \subset \mathbb {C}$ be a Weil–Petersson quasicircle. Then the geodesic curvature $k_{g_0}:S^1\rightarrow \mathbb {R}$ is a tempered distribution of order at most 2. More precisely, we have $k_{g_0}\in H^{-1/2}(S^1)$.

#### Proof

Either using the Poincaré half-plane $\mathbb {H}$ and the formula (6.1) or (4.19), we get

6.2 $$\begin{aligned} k_{g_0}=\textrm{Re}\,\left( z\frac{f''(z)}{f'(z)}\right) +1. \end{aligned}$$

Now, if $0<\varepsilon <1$ and $f_{\varepsilon }:\mathbb {D}\rightarrow \Omega$ is defined by

$$\begin{aligned} f_{\varepsilon }(z)=\frac{1}{1-\varepsilon }f((1-\varepsilon )z)\qquad z\in \mathbb {D}, \end{aligned}$$

we have (see [59], Lemma 8.2)

$$\begin{aligned} \lim \limits _{\varepsilon \rightarrow 0}\int _{\mathbb {D}}\left| \frac{f_{\varepsilon }''(z)}{f_{\varepsilon }'(z)}-\frac{f''(z)}{f'(z)}\right| ^2|dz|^2=0, \end{aligned}$$

which is equivalent by trace theory to

$$\begin{aligned} \lim \limits _{\varepsilon \rightarrow 0}\left\| \log |f'_{\varepsilon }|-\log |f'|\right\| _{\textrm{H}^{{1}/{2}}(S^1)}=0,\\ \lim \limits _{\varepsilon \rightarrow 0}\left\| \arg (f'_{\varepsilon })-\arg (f')\right\| _{\textrm{H}^{{1}/{2}}(S^1)}=0. \end{aligned}$$

Using the equivalent semi-norm for $H^s$ spaces given by

$$\begin{aligned} \left\| u\right\| _{\text {H}^{s}(S^1)}=\left( \sum _{n\in \mathbb {Z}}^{}|n|^{2s}|a_n|^2\right) ^{\frac{1}{2}},\qquad \text{ if }\;\, u(z)=\sum _{n\in \mathbb {Z}}a_nz^n, \end{aligned}$$

we deduce that

$$\begin{aligned} \lim \limits _{\varepsilon \rightarrow 0}\left\| \partial _{\theta }\log (f_{\varepsilon }')-\partial _{\theta }\log (f')\right\| _{\textrm{H}^{-{1}/{2}}(S^1)}=0. \end{aligned}$$

Since

$$\begin{aligned} \frac{f''(z)}{f'(z)}=\frac{1}{ie^{i\theta }}\partial _{\theta }\log (f'(z)), \end{aligned}$$

we deduce that $\dfrac{f''}{f'}\in H^{-1/2}(S^1)$, which concludes the proof of the lemma by (6.2).

$$\square $$

#### Remark 6.3

For other considerations related to trace spaces, see [7] and Definition 5 of [9].

#### Proposition 6.4

(See also [6]) For all $\varepsilon >0$, let $\mathbb {D}_+(0,\varepsilon )=\mathbb {H}\cap \left\{ z:|z|<\varepsilon \right\}$, where $\mathbb {H}=\mathbb {C}\cap \left\{ z:\textrm{Im}\,(z)>0\right\}$ is the Poincaré half-plane. For $\varepsilon >0$ small enough, the map $f:\mathbb {D}_+(0,\varepsilon )\rightarrow \mathbb {C}$ defined by

$$\begin{aligned} f(z)=z\,e^{i\log \log (z)}, \end{aligned}$$

where $\log (z)$ is the principal value of the logarithm on $\mathbb {H}$, is an immersion and $\log |f'|\in W^{1,2}(\mathbb {D}_+(0,\varepsilon ))$. In particular, the curve $\gamma :(-\varepsilon ,\varepsilon )\rightarrow \mathbb {C}$ such that $\gamma (t)=te^{i\log \log (t)}$ for all $t\in (-\varepsilon ,\varepsilon )$ is a part of a Weil–Petersson quasicircle.^2^ Furthermore, its geodesic curvature $k_{g_0}$ is given by

$$\begin{aligned} k_{g_0}=-\frac{1}{t(1+\log ^2(t))}+\mathrm {p.v.}\int _{-\varepsilon }^{\varepsilon }\frac{dt}{t\log (t)}. \end{aligned}$$

#### Proof

We compute

$$\begin{aligned} f'(z)&=e^{i\log \log (z)}+\frac{i}{\log (z)}e^{i\log \log (z)}=\left( 1+\frac{i}{\log (z)}\right) e^{i\log \log (z)}\\ f''(z)&=-\frac{i}{z\log ^2(z)}e^{i\log \log (z)}+\frac{i}{z\log (z)}\left( 1+\frac{i}{\log (z)}\right) e^{i\log \log (z)}. \end{aligned}$$

Therefore, we have

$$\begin{aligned} \frac{f''(z)}{f'(z)}=-\frac{i}{z\log (z)(i+\log (z))}+\frac{i}{z\log (z)}=i\frac{-1+i+\log (z)}{z\log (z)(i+\log (z))}. \end{aligned}$$

Notice that the following identity holds:

$$\begin{aligned} |i+\log (z)|^2=|\log |z|+i(1+\arg (z))|^2=\log ^2|z|+(1+\arg (z))^2. \end{aligned}$$

Therefore, we deduce that

$$\begin{aligned} \left| \frac{f''(z)}{f'(z)}\right| ^2&=\frac{(\log |z|-1)^2+(1+\arg (z))^2}{|z|^2\log ^2|z|(\log ^2|z|+(1+\arg (z))^2}\\&\le \frac{1}{\log ^2|z|}+\frac{-2\log |z|+1}{\log ^4|z|} \le \frac{2}{|z|^2\log ^4|z|}+\frac{2}{|z|^2\log ^2|z|}, \end{aligned}$$

and

$$\begin{aligned} \int _{\mathbb {D}(0,\frac{1}{2})}\left| \frac{f''(z)}{f'(z)}\right| ^2|dz|^2&\le 4\pi \int _{0}^{\frac{1}{2}}\frac{dr}{r\log ^4(r)}+4\pi \int _{0}^{\frac{1}{2}}\frac{dr}{r\log ^2(r)}\\&=\frac{4\pi }{\log (2)}+\frac{4\pi }{3\log ^2(2)}<\infty , \end{aligned}$$

which shows that $\gamma$ is a Weil–Petersson quasicircle. Then, we have by Lemma 6.1 for $z\in \mathbb {R}$

$$\begin{aligned} k_{g_0}=\textrm{Im}\,\left( \frac{f''(z)}{f'(z)}\right) =\frac{1-\log |z|+\log ^2|z|}{|z|\log |z|(1+\log ^2|z|)}=-\frac{1}{|z|(1+\log ^2|z|)}+\frac{1}{|z|\log |z|}, \end{aligned}$$

which concludes the proof of the proposition. $\quad \square$

#### Remark 6.5

In particular, we see that there exists curves whose geodesic curvature is a distribution of order 1. This curve is an example of spiral mentioned earlier in the introduction.

### A Consequence of Theorem 5.5

The new identity of $\pi \,I^L=S_3$ from Theorem 5.3 and Theorem 5.5 provides a new identity about holomorphic univalent maps of the plane.

#### Lemma 6.6

Let $\gamma \subset \mathbb {C}$ be a closed simple curve with finite Loewner energy. We have

$$\begin{aligned}&4\,\textrm{Re}\,\int _{\mathbb {D}}\left( \frac{f''(z)}{f'(z)}-2\frac{f'(z)}{f(z)}+\frac{2}{z}\right) \overline{\left( \frac{f'(z)}{f(z)}\frac{1}{1+|f(z)|^2}-\frac{2}{z}\right) }|dz|^2\\&\quad +4\int _{\mathbb {D}}\left| \frac{f'(z)}{f(z)}\frac{1}{1+|f(z)|^2}-\frac{1}{z}\right| ^2|dz|^2\\&\quad +4\,\textrm{Re}\,\int _{\mathbb {C}\setminus \overline{\mathbb {D}}}\left( \frac{g''(z)}{g'(z)}-2\frac{g'(z)}{g(z)}+\frac{2}{z}\right) \overline{\left( \frac{g'(z)}{g(z)}\frac{1}{1+|g(z)|^2}\right) }|dz|^2\\&\quad +4\int _{\mathbb {C}\setminus \overline{\mathbb {D}}}\left| \frac{g'(z)}{g(z)}\frac{1}{1+|g(z)|^2}\right| ^2|dz|^2\\&\quad +2\int _{\mathbb {D}}\log |z|\frac{4|f'(z)|^2|dz|^2}{(1+|f(z)|^2)^2}-2\int _{\mathbb {C}\setminus \overline{\mathbb {D}}}\log |z|\frac{4|g'(z)|^2|dz|^2}{(1+|g(z)|^2)^2}+4\pi =0, \end{aligned}$$

where *f* and *g* are univalent maps as in Definition 5.1.

#### Proof

Recall the definition of $S_3(\gamma )$ in Definition 5.1:

6.3 $$\begin{aligned} S_3(\gamma )&=\int _{\mathbb {D}}\left| \frac{f''(z)}{f'(z)}-2\frac{f'(z)}{f(z)}\frac{|f(z)|^2}{1+|f(z)|^2}\right| ^2|dz|^2\nonumber \\&\quad +\int _{\mathbb {C}\setminus \overline{{\mathbb {D}}}}\left| \frac{g''(z)}{g'(z)}-2\frac{g'(z)}{g(z)}\frac{|g(z)|^2}{1+|g(z)|^2}+\frac{2}{z}\right| ^2|dz|^2\nonumber \\&\quad +2\int _{\mathbb {D}}\log |z|\frac{4|f'(z)|^2|dz|^2}{(1+|f(z)|^2)^2}-2\int _{\mathbb {C}\setminus \overline{\mathbb {D}}}\log |z|\frac{4|g'(z)|^2|dz|^2}{(1+|g(z)|^2)^2}+4\pi \nonumber \\&\quad +4\pi \log |f'(0)|-4\pi \log |g'(\infty )| \end{aligned}$$

and that $\pi I^L(\gamma )=S_1(\gamma )=S_3(\gamma )$ from Theorem 5.5. Using the identity (1.5), we obtain

6.4 $$\begin{aligned} S_3(\gamma )&= S_3 (\mathfrak {i}(\gamma ))\nonumber \\&=\int _{\mathbb {D}}\left| \frac{f''(z)}{f'(z)}-2\frac{f'(z)}{f(z)}+\frac{2}{z}+2\frac{f'(z)}{f(z)}\frac{1}{1+|f(z)|^2}-\frac{2}{z}\right| ^2|dz|^2\nonumber \\&\quad +\int _{\mathbb {C}\setminus \overline{\mathbb {D}}}\left| \frac{g''(z)}{g'(z)}-2\frac{g'(z)}{g(z)}+\frac{2}{z}+2\frac{g'(z)}{g(z)}\frac{1}{1+|g(z)|^2}\right| ^2|dz|^2+4\pi \log \left|\frac{f'(0)}{g'(\infty )} \right|\nonumber \\&\quad +2\int _{\mathbb {D}}\log |z|\frac{4|f'(z)|^2|dz|^2}{(1+|f(z)|^2)^2}-2\int _{\mathbb {C}\setminus \overline{\mathbb {D}}}\log |z|\frac{4|g'(z)|^2|dz|^2}{(1+|g(z)|^2)^2}+4\pi \nonumber \\&=\int _{\mathbb {D}}\left| \frac{f''(z)}{f'(z)}-2\frac{f'(z)}{f(z)}+\frac{2}{z}\right| ^2|dz|^2+\int _{\mathbb {C}\setminus \overline{\mathbb {D}}}\left| \frac{g''(z)}{g'(z)}-2\frac{g'(z)}{g(z)}+\frac{2}{z}\right| ^2 |dz|^2 \nonumber \\&\quad +4\pi \log \left|\frac{f'(0)}{g'(\infty )} \right|\nonumber \\&\quad +4\,\textrm{Re}\,\int _{\mathbb {D}}\left( \frac{f''(z)}{f'(z)}-2\frac{f'(z)}{f(z)}+\frac{2}{z}\right) \overline{\left( \frac{f'(z)}{f(z)}\frac{1}{1+|f(z)|^2}-\frac{2}{z}\right) }|dz|^2\nonumber \\&\quad +4\,\textrm{Re}\,\int _{\mathbb {C}\setminus \overline{\mathbb {D}}}\left( \frac{g''(z)}{g'(z)}-2\frac{g'(z)}{g(z)}+\frac{2}{z}\right) \overline{\left( \frac{g'(z)}{g(z)}\frac{1}{1+|g(z)|^2}\right) }|dz|^2\nonumber \\&\quad +4\int _{\mathbb {D}}\left| \frac{f'(z)}{f(z)}\frac{1}{1+|f(z)|^2}-\frac{1}{z}\right| ^2 |dz|^2 +4\int _{\mathbb {C}\setminus \overline{\mathbb {D}}}\left| \frac{g'(z)}{g(z)}\frac{1}{1+|g(z)|^2}\right| ^2|dz|^2\nonumber \\&\quad +2\int _{\mathbb {D}}\log |z|\frac{4|f'(z)|^2|dz|^2}{(1+|f(z)|^2)^2}|dz|^2-2\int _{\mathbb {C}\setminus \overline{\mathbb {D}}}\log |z|\frac{4|g'(z)|^2|dz|^2}{(1+|g(z)|^2)^2}+4\pi . \end{aligned}$$

Comparing (6.3) and (6.4), we get the claimed identity. $\quad \square$

Let us check the formula in the case $\gamma =S^1$. In this case, we have $f(z)=z$, and $g(z)=z$, and the sum in Lemma 6.6 simplifies to

6.5 $$\begin{aligned}&4\int _{\mathbb {D}}\left| \frac{1}{z}\frac{1}{1+|z|^2}-\frac{1}{z}\right| ^2|dz|^2+4\int _{\mathbb {C}\setminus \overline{\mathbb {D}}}\left| \frac{1}{z}\frac{1}{1+|z|^2}\right| ^2|dz|^2+2\int _{\mathbb {D}}\frac{4\log |z||dz|^2}{(1+|z|^2)^2}\nonumber \\&\quad -2\int _{\mathbb {C}\setminus \overline{\mathbb {D}}}\frac{4\log |z||dz|^2}{(1+|z|^2)^2}+4\pi . \end{aligned}$$

First, we have

6.6 $$\begin{aligned} \int _{\mathbb {D}}\left| \frac{1}{z}\frac{1}{1+|z|^2}-\frac{1}{z}\right| ^2|dz|^2=\int _{\mathbb {D}}\left| \frac{\overline{z}}{1+|z|^2}\right| ^2|dz|^2=\int _{\mathbb {D}}\frac{|z|^2|dz|^2}{(1+|z|^2)^2}, \end{aligned}$$

and an immediate change of variable $z\mapsto \dfrac{1}{z}$ shows that

6.7 $$\begin{aligned} \left\{ \begin{aligned}&\int _{\mathbb {C}\setminus \overline{\mathbb {D}}}\left| \frac{1}{z}\frac{1}{1+|z|^2}\right| ^2|dz|^2=\int _{\mathbb {D}}\frac{|z|^2|dz|^2}{(1+|z|^2)^2}\\&\int _{\mathbb {C}\setminus \overline{\mathbb {D}}}\frac{\log |z||dz|^2}{(1+|z|^2)^2}=-\int _{\mathbb {D}}\frac{\log |z||dz|^2}{(1+|z|^2)^2}. \end{aligned}\right. \end{aligned}$$

By previous computations (Remark 3.8), we have

6.8 $$\begin{aligned} \left\{ \begin{aligned} \int _{\mathbb {D}}\frac{4|z|^2|dz|^2}{(1+|z|^2)^2}&=4\pi \log (2)-2\pi \\ \int _{\mathbb {D}}\frac{4\log |z||dz|^2}{(1+|z|^2)^2}&=-2\pi \log (2), \end{aligned}\right. \end{aligned}$$

which shows, by (6.6), (6.7) and (6.8), that the sum (6.5) is equal to

$$\begin{aligned}&2\int _{\mathbb {D}}\frac{ 4|z|^2|dz|^2}{(1+|z|^2)^2}+4\int _{\mathbb {D}}\frac{4\log |z||dz|^2}{(1+|z|^2)^2}+4\pi \\&\quad =2\left( 4\pi \log (2)-2\pi \right) +4\left( -2\pi \log (2)\right) +4\pi =0, \end{aligned}$$

as expected.

## Footnotes

1 It is necessary to assume that the curve is smooth for one will need to recurse to the Froebenius theorem below. Furthermore, the formula for the Loewner energy using the zeta-regularised determinants [59] only works for smooth (or at least $C^3$) curves ([53], Corollary 3.12).
